# Phase Transitions and Dynamics in Mixed Three- and Low-Dimensional Lead Halide Perovskites

**DOI:** 10.1021/acs.chemrev.3c00532

**Published:** 2024-02-29

**Authors:** Mantas Simenas, Anna Gagor, Juras Banys, Miroslaw Maczka

**Affiliations:** † Faculty of Physics, Vilnius University, Sauletekio 3, LT-10257 Vilnius, Lithuania; ‡ Institute of Low Temperature and Structure Research, Polish Academy of Sciences, Okólna 2, PL-50-422 Wroclaw, Poland

## Abstract

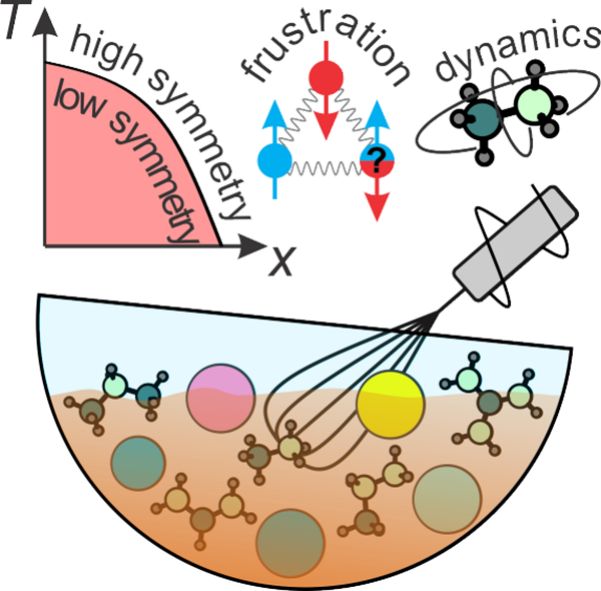

Lead halide perovskites are extensively investigated as efficient solution-processable materials for photovoltaic applications. The greatest stability and performance of these compounds are achieved by mixing different ions at all three sites of the APbX_3_ structure. Despite the extensive use of mixed lead halide perovskites in photovoltaic devices, a detailed and systematic understanding of the mixing-induced effects on the structural and dynamic aspects of these materials is still lacking. The goal of this review is to summarize the current state of knowledge on mixing effects on the structural phase transitions, crystal symmetry, cation and lattice dynamics, and phase diagrams of three- and low-dimensional lead halide perovskites. This review analyzes different mixing recipes and ingredients providing a comprehensive picture of mixing effects and their relation to the attractive properties of these materials.

## 1 Introduction

In the past decade, lead halide perovskites APbX_3_ (A = molecular or inorganic cation, X = halide anion) and related materials have received unprecedented attention due to their potential applications for photovoltaic devices such as efficient solar cells and light-emitting diodes (LEDs).^1−7^ The power conversion efficiency of solar cells based on these solution-processable compounds experienced an extraordinary boost and currently exceeds 25%,^8,9^ with the best performance and stability achieved using perovskites with mixed compositions.^10,11^

Depending on the type and relative size of the constituent ions, lead halide perovskites can form either three-dimensional (3D) or lower-dimensional structures.^12,13^ The stability of the 3D perovskite structure is determined by the tolerance factor first introduced by Goldschmidt for inorganic oxides^14^ and later modified for organic–inorganic perovskites by introduction of the effective ionic radius *r*_eff_ of the molecular cations.^15,16^ This semiempirical approach is based on the assumption of rotational freedom of the molecular cations around their center of mass and work surprisingly well for organic–inorganic perovskites, thus confirming the highly dynamical nature of the these cations within the perovskite cavities. The anisotropic size of the molecular cations, together with the number and the type of hydrogen donors for N–H···X hydrogen bonding, are important in shaping the distortions of the ordered or partially ordered low-temperature phases.^17^ When the tolerance factor falls in the range of 0.9 to 1.0, 3D perovskites of cubic symmetry are formed, while a tolerance factor of 0.80 to 0.89 typically leads to lower symmetry. Low-dimensional structures typically emerge, when the tolerance factor is higher than 1 or lower than 0.7.

The low-dimensional lead halides can crystallize in several stoichiometries and can be regarded as two-, one-, or zero-dimensional (2D, 1D, and 0D) structures. The 2D hybrid organic–inorganic lead halides are derived from 3D perovskites by removing inorganic layers along the (100), (110), or (111) directions, and the most common stoichiometry is A′_2_A_*n*–1_Pb_*n*_X_3*n*+1_ or A′′A_*n*–1_Pb_*n*_X_3*n*+1_, where A, A′, and A*″* are a small monovalent, large monovalent and large divalent cation, respectively.^18^ 1D structures have various stoichiometries, and they contain chains composed of corner-, edge-, or face-shared PbX_6_ octahedra separated by organic cations.^19^ 0D structures also have various stoichiometries, for instance, A_4_PbX_6_, and they consist of completely isolated PbX_6_ octahedra or lead halide clusters.^20,21^ In most cases, the isolation is achieved by employment of large organic cations. As discussed by Akkerman and Manna, these types of lead halides, especially 1D and 0D, have little in common with the 3D perovskite structure, and therefore they should not be identified as perovskites.^22^ However, the term “perovskite” for these low-dimensional structures has been widely accepted in the literature, and in this review we will call such structures perovskites.

**Figure 1:**
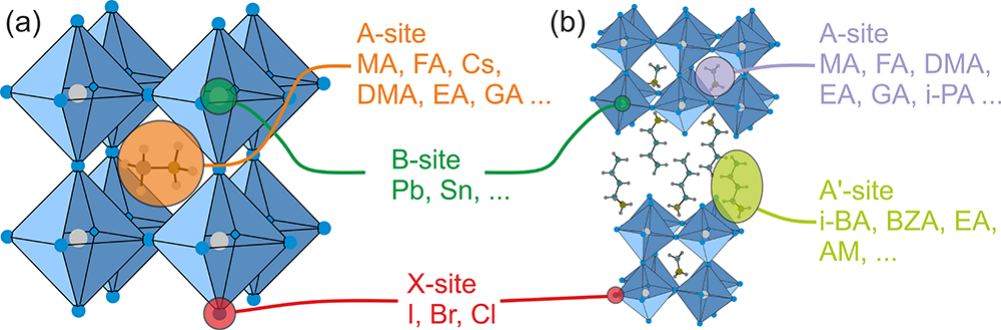
Mixing in the (a) 3D and (b) lower-dimensional (Ruddlesden–Popper) lead halide perovskites.

The 3D structures of lead halide perovskites have a typical APbX_3_ topology, where the A-site cation is enclosed in an inorganic lead-halide cage formed out of the corner-sharing PbX_6_ octahedra (Figure 1a). Currently, there are five known A-site cations (methylammonium (MA), formamidinium (FA), methylhydrazinium (MHy), aziridinium (AZR), and Cs^+^), which stabilize the 3D perovskite structure (Table 1). Among these, hybrid organic–inorganic MAPbI_3_ and FAPbI_3_, and all-inorganic CsPbX_3_ perovskites are the most promising for solar cell and related applications.^8,9^

**Table 1:** The Most Popular A-site Cations Used for Mixing and Formation of 3D Lead Halide Perovskites^a^

A-site cation	*r*_eff_ (pm)	tolerance factor	dipole moment (D)
Methylammonium (MA) CH_3_NH_3_^+^	217	0.91	2.26
Hydrazinium (HY) H_3_N-NH_2_^+^	217	0.91	3.24
Formamidinium (FA) HC(NH_2_)_2_^+^	253	0.99	0.22
Imidazolium (IM) C_3_N_2_H_5_^+^	258	1.00	1.42
Methylhydrazinium (MHy) CH_3_NH_2_NH_2_^+^	264	1.01	2.80
Dimethylammonium (DMA) (CH_3_)_2_NH_2_^+^	272	1.03	1.52
Ethylammonium (EA) (C_2_H_5_)NH_3_^+^	274	1.03	3.95
Acetamidinium (ACE) H_3_C(NH_2_)_2_^+^	277	1.04	1.33
Guanidinium (GA) C(NH_2_)_3_^+^	278	1.04	0
Cs^+^	167	0.81	-
Rb^+^	152	0.78	-

a The effective cation radius *r*_eff_ and the tolerance factor (in the lead iodide framework) were calculated by following the procedure described in ref (15). The electric dipole moment was calculated for the isolated cation using DFT (B3LYP/6-31G*).

Mixing in 3D perovskites can be performed in all three sites of the lattice (Figure 1a),^10,11^ although the B-site (metal) mixing is significantly less frequent. The choice of organic and inorganic cations for the A-site mixing is rather broad with the most popular cations listed in Table 1 together with their corresponding effective ionic radii, tolerance factors, and dipole moments. The mixing ingredients at the X-site are limited to I^–^, Br^–^, and Cl^–^ anions, which have different ionic radii and affinities to form H-bonds with the molecular cations.

Upon mixing with high amounts of large molecular cations, the 3D perovskite structure becomes unstable due to the increase of the tolerance factor.^15,16^ In this limit, typically a phase separation occurs in the 3D perovskite and low-dimensional perovskite-like structures. Currently, the low-dimensional perovskites are widely applied in fabrication of solar cells,^23−25^ LEDs,^4−7,19,25−28^ photodetectors,^26,29^ piezoelectric sensors,^26,30^ scintillators,^19,31^ nonlinear optical (NLO) switches,^32,33^ and dielectric switches.^34,35^ These compounds occur in various topologies comprising one or two organic cations, where examples of the former compounds are A_2_′PbX_4_ and A″PbX_4_ layered perovskites (A′ = monovalent cation, A″ = divalent cation).^36−41^ There are many reports on the formation of lead halide perovskites comprising two organic cations. One of these groups constitutes an alternating cation in the interlayer space (ACI) perovskites with the general formula A′A′′_*n*_Pb_*n*_X_3*n*+1_ (*n* ≥ 1).^42−44^ Another and very large group constitutes quasi-layered Dion-Jacobson (DJ, A′′A_*n*–1_Pb_*n*_X_3*n*+1_) and Ruddlesden–Popper (RP, A′_2_A_*n*–1_Pb_*n*_X_3*n*+1_) phases with *n* ≥ 2, where A, A′, and A″ is a small “perovskitizer” cation, large spacer monovalent cation. and large spacer divalent cation, respectively (Figure 1b).^23,45−47^ Most of the small cations are the same as those used in the synthesis of pure and mixed-cation 3D perovskites, e.g., MA, FA, DMA, GA, etc., but the relaxed tolerance factor in such low-dimensional perovskites also allows the incorporation of larger cations such as isopropylammonium (i-PA) and 1,2,4-triazolium (TZ) into the perovskite cavities.^48,49^

There are numerous studies and reviews on the mixing effects on the photovoltaic performance, optical properties, and stability of lead halide perovskites (see e.g. refs (11, 50−52)). However, despite these properties highly depending on the atomistic picture of the mixed compositions,^53−57^ a systematic understanding of how mixing affects the structural and dynamics phenomena in these materials is still lacking. This is especially important in the context of classical inorganic perovskites, where mixing is known to drastically alter the long-range order and lattice dynamics, causing formation of exotic frustrated phases such as relaxors and electric dipole glasses.^58−62^

The main goal of this review is to provide the first comprehensive and systematic understanding and highlight common aspects of mixing effects on the structural and dynamic properties of lead halide perovskites. The introductory section of the review is structured to briefly cover the most important photovoltaic aspects of mixing followed by a brief summary of ion substitution effects in classical inorganic compounds and the most common tools employed to study the structural and dynamic properties of mixed lead halide perovskites. The subsequent sections provide an in-depth analysis of the structural phase transitions in nonmixed parent compounds forming a basis for the subsequent chapters, which deal with the A-, X-, and B-site mixing in 3D perovskites. Afterward, the mixing effects in the low-dimensional lead halide systems are discussed. The review ends with the generalization of the common aspects observed in different mixed systems followed by an outlook, which draws a roadmap for further studies of these intricate systems.

### 1.1 Benefits of Mixing in Lead Halide Perovskites and Related Compounds

It is well-known that one of the main limiting factors of the most popular 3D perovskites MAPbI_3_ and FAPbI_3_ is their low chemical stability in a humid environment and under exposure to light as well as low thermal stability. One of the main strategies to increase stability is the A-site mixing with larger organic cations like GA, DMA, IM, EA, HY, ACE, hydroxyethylammonium (HEA), or thioethylammonium (TEA)^63−74^ (see Table 1 for the most popular A-site cations). For instance, mixing of MAPbI_3_ with 5% of GA led to a 62% decrease in the degradation rate under illumination.^63^ Another very important benefit of mixing at the A-sites is a better photovoltaic,^63−67,69−71,75−80^ photodetecting,^68,73,74,81^ and photoluminescence (PL) performance^63^ as well as tunability of the NLO properties.^82^ For example, mixing of MAPbI_3_ with GA increased PL lifetimes and enhanced efficiency of solar cell devices.^63^

There are numerous experimental and theoretical reports showing that the A-site mixing affects the electronic structure including band gap and electron–phonon coupling.^69,80,81,83−87^ Studies of MA_1–*x*_Cs_*x*_PbI_3_ and FA_1–*x*_Cs_*x*_PbI_3_ showed that the band gap gradually increased with increasing Cs content, and this effect was attributed to shortening of the Pb–I bond lengths, when the larger MA cation is replaced by the smaller Cs^+^.^83,87^ Mixing of MAPbX_3_ compounds with larger cations also revealed a gradual increase of the band gap with increasing level of mixing. For instance, in the MA_1–*x*_DMA_*x*_PbBr_3_ solid solution, the band gap showed a change from 2.17 eV for *x* = 0 to 2.23 eV for *x* = 0.3,^86^ while in the MA_1–*x*_GA_*x*_PbI_3_ system the change was from 1.49 eV (*x* = 0) to 1.53 eV *x* = 0.22.^80^ This behavior was attributed to local distortions of the inorganic sublattice, especially alteration of the X–Pb–X bond angles mediated by the new H-bonds.^69,84^ Computations performed for the MA_1–*x*_DMA_*x*_PbI_3_ and MA_1–*x*_GA_*x*_PbI_3_ solid solutions suggested that the A-site mixing effectively suppressed the strength of the electron–phonon coupling and that this effect is stronger for the GA-based system.^81^ It was suggested that the reduced strength of the electron–phonon coupling may also contribute to the longer carrier lifetimes in the mixed perovskite.^81^ Latter temperature-dependent studies of the PL properties of MA_1–*x*_FA_*x*_PbI_3_ solid solutions showed that, while the effective phonon energy is almost independent of the FA content with an average value of about 5 meV, the electron–phonon coupling strength adopts two different values: about 10 meV for the end members and about 17 meV for the intermediate compositions.^88^ Thus, for this system the mixing led to the increase of the electron–phonon coupling.

Improved photovoltaic performance, stability, and tunability of PL color and intensity can also be realized by doping at the B-sites with Sn^2+^^71,89−97^ or other metal cations.^98−101^ The improved properties after mixing at the B-site have been attributed to change of the band gap, decrease of the electron–phonon coupling strength, increase in the material defect formation energy, and increased charge carrier lifetimes due to changes in the cation dynamics.^67,84,89,91,93−97,102^ It is worth adding that band gaps of tin-based perovskites are narrower compared to lead-based analogues. Consequently, for many B-site mixed perovskites comprising divalent lead and tin, for instance CsPb_1–*x*_Sn_*x*_Br_3_ and FA_0.75_Cs_0.25_Pb_1–*x*_Sn_*x*_I_3_, the band gap exhibits gradual narrowing with increasing Sn content.^103,104^ Interestingly, in some cases, the mixed lead–tin perovskites possess narrower band gaps compared to the end members, making these mixed system very attractive for photovoltaic applications. This behavior (band gap bowing) was reported for MAPb_1–*x*_Sn_*x*_I_3_ and FAPb_1–*x*_Sn_*x*_I_3_ solid solutions, for which the intermediate compounds showed corresponding band gaps of 1.17 eV and near 1.3 eV, whereas the band gap of MAPbI_3_, MASnI_3_, FAPbI_3_, and FASnI_3_ was 1.55, 1.30, 1.55, and 1.39 eV, respectively.^89,94^ It was argued that this anomalous behavior emerges from the nonlinear mixing of Pb and Sn orbitals in the band edges.^105^ In addition to tuning of the optoelectronic properties, the B-site doping is also highly desirable due to high lead toxicity.^94,98^

It is important to note that, although the photoactive black phases of FAPbI_3_ and CsPbI_3_ have great application potential, they are thermodynamically unstable and transform into the nonphotoactive yellow phases in an ambient humid atmosphere.^106,107^ The black phases can be stabilized by preparing FA/Cs, FA/MA, or FA/Rb solid solutions,^76,108−110^ by mixing FAPbI_3_ with large organic cations,^72,79^ or by mixing both the A- and X-sites.^95,111−115^ In the latter case, Br^–^ anions are usually used at the X-sites.^111−113^ Mixing at this site is also widely used to tune optical band gaps and improve photovoltaic properties.^9,116−118^ The effect of the halide exchange on the band gap is much more pronounced compared to the A- or B-site mixing. In general, the band gap in Br/Cl and Br/I systems strongly narrows with increasing Br and I content, respectively, due to the increased Pb–X bond length.^85,119^ As a result, the PL color is altered from UV for chlorides to near-IR for iodides.^9,116−119^ Mixing at the X-site also tunes the NLO and dielectric properties.^118,120,121^

Another very important aspect of mixing in MAPbI_3_ is the lattice symmetrization and cubic phase stabilization achieved by suppression of the structural phase transitions. In addition to lower defect density, longer carrier lifetime, larger wide absorbance range, and enhanced stability,^68,78,122^ stabilization of the cubic phase below room temperature is desirable, as it prevents the phase-transition-induced accumulation of the lattice fatigue and strain, which are harmful for device operation.^123^

In the case of lower-dimensional perovskites, a partial mixing was reported only at the B- and X-sites and mainly for the A′_2_PbX_4_ and A″PbX_4_ layered perovskites. Similarly to the 3D analogues, the mixing at the X-sites strongly modifies the band gap, and therefore this mixing was chosen as a strategy to tune the PL color and to obtain materials exhibiting white-light emission.^36−41^ The X-site mixing may also lead to tunable dielectric properties.^124^ Mixing at the B-site is less common, but it was shown that substitution of lead with tin narrows the band gap and induces broadband PL.^125−127^ Replacing of lead with other metal cations like Mn^2+^ or Sb^3+^ is also a way to tailor the color and PL quantum yield.^128,129^ Although partial mixing at the A′- and A″-sites of A′_2_PbX_4_ and A″PbX_4_ perovskites (RP and DJ phases with *n* = 1) has not been reported, there are many reports on the formation of lead halide perovskites comprising two organic cations. For the ACI perovskites, employment of two different organic cations offers a potential way to tailor the spin-based and PL properties.^42−44^ Employment of two organic cations in the DJ and RP phases has many benefits. First of all, it allows controlling the optoelectronic and PL properties,^23,46,47^ but it may also induce other functional responses like ferroelectricity,^130−132^ antiferroelectricity,^133^ pyroelectricity,^134^ or NLO properties.^133,135^ Properties of DJ and RP phases with *n* ≥ 2 can be further fine-tuned by mixing at the A′-,^136,137^ A″-,^138,139^ A-,^138,140,141^ and B-sites.^142,143^ Although many works reported preparation of these phases with different halide anions,^144^ recent studies showed that this is not a real mixing since halide anions preferentially occupy different sites.^145^

Despite a vast number of works concentrating on the exploitation of the discussed improvements for device fabrication, mixing effects on the structural and dynamic properties of lead halide perovskites are significantly less studied and understood. It is reasonable to assume that mixing in these compounds would result in a similar behavior as observed in classical inorganic solid solutions, where the substitution effects are significantly better comprehended.

### 1.2 Mixing Effects in Classical Inorganic Compounds

**Figure 2:**
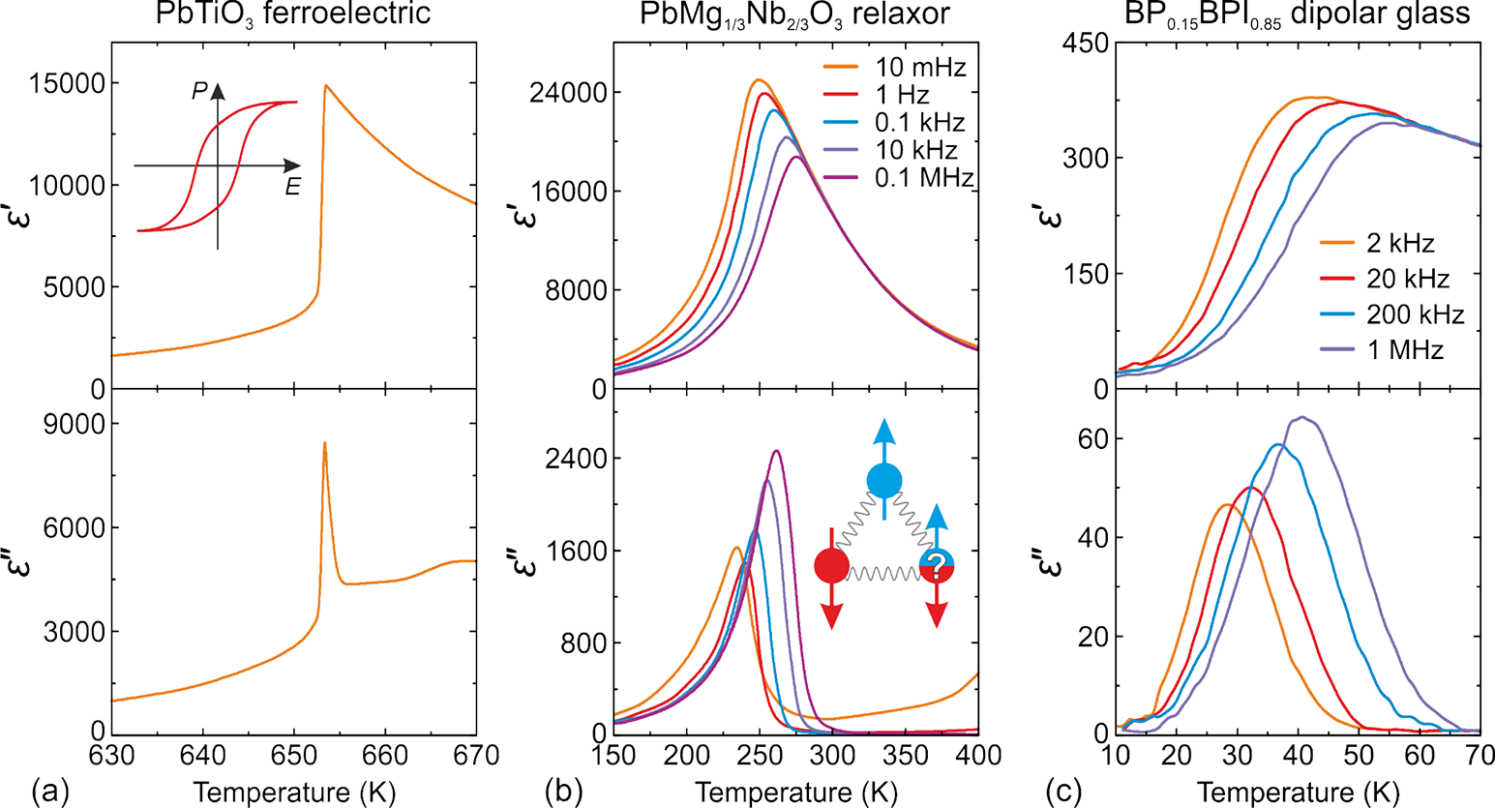
Temperature dependence of the complex dielectric permittivity of (a) PbTiO_3_ perovskite ferroelectric, (b) canonical relaxor PbMg_1/3_Nb_2/3_O_3_, and (c) dipolar glass (betaine–phosphate)_0.15_(betaine–phosphite)_0.85_. A typical ferroelectric hysteresis loop is presented in the inset in (a). Inset in (b) shows a classical example of dipole frustration in the antiferrommagnetic Ising model on a 2D triangular lattice. (a) Adapted with permission from ref (154). Copyright 1971 Taylor & Francis. (b) Adapted with permission from ref (62). Copyright 2006 Springer Nature. (c) Adapted with permission from ref (153). Copyright 1996 IOP Publishing.

It is well established that compositional disorder may significantly change the structural and dynamic properties of classical inorganic ferroelectrics and related materials.^58−62^ A ferroelectric compound exhibits spontaneous electric polarization, which can be flipped (switched) by the external electric field, as evident from a ferroelectric hysteresis loop measurements (see inset in Figure 2a). Typically, these compounds also exhibit structural phase transitions from the paraelectric (disordered in order–disorder ferroelectrics) to the ferroelectric (ordered) state with changing temperature or pressure.^146^

In some of ferroelectric and related compounds, ion substitution introduces competing interactions, which cause a complete suppression of the structural phase transitions and frustration of electric dipoles or orientational degrees of freedom (inset in Figure 2b). As a result, the long-range dipole order is replaced by the frustrated phases such as dipolar or orientational glass (e.g., (KBr)_1–*x*_(KCN)_*x*_),^59−61,147,148^ strain glass (e.g., Ti_50–*x*_Ni_50+_*_x_*),^149^ or relaxor (e.g., PbMg_1/3_Nb_2/3_O_3_).^58,62,150,151^ The dipolar glass phase does not exhibit structural phase transitions and instead is characterized by freezing of electric dipoles at low temperature into structures, which lack long-range order.^59^ The relaxors also do not show structural phase transitions, though they are more related to ferroelectrics, as in relaxor compounds, the electric polarization may be induced by application of a sufficiently strong external electric field.^62^ The relaxor phenomena occurs due to random charge or bond disorder in the system, which also leads to formation of polar nanoregions, which grow and freeze with decreasing temperature, as confirmed by neutron diffraction.^152^

The frustration of electric dipoles in mixed compounds can significantly alter their dielectric response, which is typically described by the complex dielectric permittivity ε* = ε′ – *iε*″. Here, the real part ε′ corresponds to the dielectric permittivity (dielectric constant), while the dielectric losses (dissipation) are described by the imaginary part ε″. The dielectric response associated with dipolar glass and relaxor phases is usually very broad with respect to both probing frequency and temperature,^58,60^ which is caused by a broad distribution of the dipolar relaxation times. This behavior is in sharp contrast to compounds exhibiting structural phase transitions, which are typically accompanied by sharp anomalies in the material properties (Figure 2a). The temperature dependence of ε* for a canonical relaxor PbMg_1/3_Nb_2/3_O_3_ (PMN) is presented in Figure 2b, showing a highly frequency-dependent peak in both real ε′ and imaginary ε″ parts of the dielectric permittivity.^62^ As the frequency decreases, the ε′ peak becomes higher, and its position shifts toward a lower temperature. This dispersion of the dielectric permittivity covers a very wide range of frequency from mHz to GHz.^58^ A dielectric response of a typical dipolar glass (betaine–phosphate)_0.15_(betaine–phosphite)_0.85_ (BP_0.15_PBI_0.85_) is presented in Figure 2c also exhibiting a broad dispersion of ε* with a less pronounced maximum of ε′.^153^ Note that the dielectric responses of ferroelectrics, relaxors, and dipolar glasses exhibit the Curie–Weiss law (ε′ ∼ 1/*T*) at high temperatures away from the anomalies.^62,153^ Hence, to distinguish these compounds, the temperature-dependent dielectric measurements in a wide range of temperature and probing frequency are essential.

Another signature of dipolar glass and relaxor phases is divergence of the dipolar relaxation time due to freezing of electric dipoles or dipolar clusters. In such a case, the dipolar dynamics are described by the Vogel–Fulcher law^60,62,155,156^ instead of the typical Arrhenius behavior. In addition, due to the frustrated nature, these phases exhibit higher entropy at low temperature, which results in excess heat capacity *C*_*p*_ best visible as a maximum in the *C*_*p*_/*T*^3^ representation.^148,157^ In analogy with the classical inorganic compounds, similar substitution effects can be expected in the mixed lead halide perovskites and related compounds.

In addition to the fundamental interest, the frustrated phases of classical inorganic compounds have a wide range of applications.^58^ For example, due to large value, slow variation with temperature, and low-loss dielectric permittivity, relaxors are used as dielectric media in ceramic capacitors and tunable microwave devices.^158^ Some solid solutions of relaxors with ferroelectrics also exhibit a very large electromechanical coupling and piezoelectric response making them widely applicable in piezoelectric devices in transducers and actuators.^159^ Some relaxor materials also exhibit a large electrocaloric effect that is promising for novel cooling applications.^160^

### 1.3 Methods to Study Mixing Effects in Lead Halide Perovskites

Here, we briefly discuss the main techniques used to study how mixing affects the structural properties and dynamics of lead halide perovskites and related compounds. Some of these techniques are summarized in detail in our recent review on molecular spectroscopy of hybrid perovskites.^161^

#### 1.3.1 Diffraction Techniques

The conventional diffraction-based methods provide information on the long-range crystal structure, evolution of the crystal symmetry, and lattice parameters upon temperature change and mixing and thus are essential for construction of the concentration–temperature phase diagrams (see e.g. refs (162−166)). The presence of complex twinning in the low-temperature phases of lead halide perovskites makes it challenging to analyze partially or completely overlapped patterns obtained from single-crystal X-ray diffraction (SCXRD) methods, resulting in a multidomain diffraction. Furthermore, both single-crystal and powder XRD (PXRD) intensities are primarily dominated by the anionic sublattice due to the large scattering factors of lead and halide atoms in comparison to carbon and nitrogen, which makes it difficult to determine the positions of organic cations. Moreover, even in the case of pure lead halide perovskites, both methods frequently lack the requisite sensitivity to identify subtle changes in crystal symmetry, leading to a narrow separation of the diffracted peaks. The introduction of mixing introduces an additional layer of complexity. Therefore, significantly more advanced diffraction techniques that rely on highly monochromatic, bright synchrotron, and neutron radiation sources are often utilized to obtain the structural information.^74,167−172^

In general, disordered systems pose a significant challenge to crystal structure solution.^173,174^ In mixed organic–inorganic materials, frustration can affect either the molecular or inorganic sublattices or both. The organic components in lead halide hybrids are anisotropic and display strong temperature-dependent dynamics. Additionally, they tend to be anchored in the inorganic framework through H-bond interactions at low temperatures experiencing off-center displacements. In these instances, the ordering of the molecular part induces distortion in the inorganic component. Similarly to dipolar glasses, the freezing of molecular motions can result in the random distribution of molecular dipole moments. It may also lead to correlated arrangements and formation of ordered nanodomains, which are somewhat similar to the relaxor behavior. Thus, another important aspect of structural analysis of hybrid lead halide perovskites concerns local order and short-range interactions.

The XRD methods that rely on the periodicity of the structure give only the time-averaged positions of molecular cations and inorganic framework over the measured sample volume. The elastic scattering results in sharp Bragg peaks, whereas static or dynamic deviations from the average long-range structure manifest as additional intensities in-between or around the main peaks and are called diffuse scattering. The diffuse scattering may originate both from static local atomic correlations or from dynamic displacements due to the lattice dynamics (thermal diffuse scattering). Insight into short-range interactions and local structural distortions can be probed via total X-ray scattering, which has recently become more viable due to the advancement of high-flux, single-crystal X-ray diffuse scattering, synchrotron wide-angle X-ray total scattering, and neutron diffuse scattering from single crystals and powders.^175−177^

The importance of the advanced scattering techniques is evident from the recent diffuse scattering experiments probing short-range order in the average high-temperature cubic phase of MAPbI_3_, where the local structure was found to be of lower symmetry.^178^ Dynamical instabilities in MAPbI_3_ were also evidenced by high energy resolution inelastic X-ray (HERIX) scattering,^179^ while total X-ray scattering revealed dynamic nanodomains of noncentrosymmetric local structure. In CsPbI_3_ nanoparticles, the combined Debye scattering equation and pair distribution function approach based on total X-ray scattering evidenced the formation of orthorhombic subdomains in the room- and high-temperature phases,^180^ whereas two-dimensional dynamical correlations in real space arising from the peculiar CsPbBr_3_ lattice dynamics were found in the high temperature cubic structure of bulk single crystals.^181^

#### 1.3.2 Calorimetric Techniques

Calorimetric methods such as differential scanning calorimetry (DSC) and heat capacity *C*_*p*_ measurements are frequently employed to detect the thermodynamic anomalies occurring due to the structural phase transitions in pure and mixed lead halide perovskites.^164−166,182−186^ These experiments also yield the entropy change associated with the phase transition, which is used to characterize the degree of ordering and character (order–disorder, displacive) of the transition. In addition, the low-temperature *C*_*p*_ measurements of the mixed compounds may provide information on the higher entropy states originating from the dipolar glass or relaxor phase, as revealed in the *C*_*p*_/*T*^3^ representation.^148,157,163,164^

#### 1.3.3 Spectroscopic Techniques

Various spectroscopic techniques probing vastly different time scales can be employed to study the dynamics and structural properties of mixed lead halide perovskites.^161^

The broadband dielectric spectroscopy (DS) is a method of choice to study the dynamics and ordering of electric dipoles, phase transitions, and correlated ordered (e.g., ferroelectric) and disordered (dipolar glass) phases.^59,62,148,150^ These phenomena are typically imprinted in the measured frequency and temperature responses of the complex dielectric permittivity ε* = ε′ – *iε*″ of the studied material (see e.g. Figure 2). The strength of the DS stems from the ability to probe various processes, which involve change of electric polarization. These include charge transport and accumulation, polar (e.g., ferroelectric) domain dynamics, molecular rotation, dipole relaxation, lattice (phonon) dynamics, and electronic polarization.^187^ As these processes occur on vastly different time scales ranging from minutes to fs, the DS experiments typically require measurements using a broad range of probing frequency of the applied electric field. In a typical frequency spectrum for a dipolar relaxation, the real value of ε* exhibits a decrease, while the imaginary part shows a peak, as the probing frequency crosses the frequency associated with the studied dipolar dynamics.

To probe different dipolar processes occurring in lead halide perovskites, ultra broadband dielectric experiments extending to the GHz frequency range may be necessary because of the fast reorientation of organic cations on the ns and ps time scales. This poses significant experimental challenges, as simple capacitor-type measurements are no longer suitable, and instead conceptually different and more complicated coaxial and waveguide setups must be employed.^188,189^ In addition to the information on the phase transitions and dipole dynamics, the DS provides the dielectric permittivity value, which directly affects the exciton binding energy and consequently the performance of the photovoltaic devices.^53,55,56,183,190−192^

Raman and infrared (IR) spectroscopy is another versatile tool well-suited for investigation of the mixing effect in lead halide perovskites and related compounds.^69,88,118,165,193^ These techniques can probe the local molecular environment, degree of disorder, symmetry, molecular vibrations, and lattice phonons,^194−199^ all of which to some extent can be affected by mixing.^69,88,118,165,193,200^ IR spectroscopy can also provide information on the frequency-dependent dielectric permittivity, electron–phonon coupling strength, and polaron masses.^196,201^ The temperature-dependent Raman and IR measurements of the molecular cation and lattice modes typically are highly sensitive to the structural phase transitions,^88,165,185,194−199,202^ complementing other techniques for construction of the phase diagrams.

Nuclear magnetic resonance (NMR) is another well-established spectroscopic technique used to probe the dynamics and phase transition in (mixed) lead halide perovskites and related compounds.^75,102,172,203−206^ It is also frequently employed to determine the stoichiometry and solubility limits of the mixed compounds.^166,207,208^ The basis of this method relies on the nuclear Zeeman interaction, which results in a splitting of the nuclear spin energy levels in the external magnetic field. The splitting is also affected by the local nuclear spin environment, which may reflect the local symmetry and degree of disorder introduced by mixing. Information about the molecular cation dynamics on a time scale ranging from ps to hours can be obtained from the spectra of the quadrupolar nuclei and relaxation time measurements.^102,204,209,210^ A related technique used to study mixing effects in lead halide perovskites is a nuclear quadrupole resonance, which is performed in the absence of the external magnetic field.^172,203^ These experiments probe the interaction of the electric field gradient with the nuclear quadrupole moment, which is very sensitivity to the changes of the local symmetry and environment of the studied nucleus, providing information on the local dynamics and structural phase transitions.

Molecular dynamics can be also studied using a quasielastic neutron scattering (QENS) technique. QENS is a limiting case of inelastic neutron scattering, in which broadening of the elastic line is due to a small energy transfer between the neutron and the atoms in the sample. QENS probes the energetically broadened signal around the elastic line and measures energy transfer down to the sub-μeV regime. This allows access to low energy collective motions, diffusional motions, relaxation processes, and molecular reorientations.^211,212^ Thus, QENS is often used to provide information about the dynamics (vibrational, rotational, and translational diffusive processes at the molecular scale) on time scales from ps to μs and on length scales from 1 to 500 Å.^211−213^ This technique finds application in many scientific topics, including polymers, dynamics of proteins, hydrogen dynamics, etc.^211,212^ QENS proved to be a very powerful method to determine the modes, characteristic correlation times, and activation energies of the molecular cation motion in lead halide perovskites.^213−217^

#### 1.3.4 Computational Techniques

Computational techniques such as the density functional theory (DFT), Monte Carlo (MC), and molecular dynamics (MD) simulations provide information on the microscopic origins of the molecular cation and lattice dynamics, structural phase transitions, and mixing effects.^218^ The DFT calculations can yield the atomistic picture of the ordered phases^164,166,219−221^ but are constrained to small system sizes and poor ability to probe entropic effects. The temperature effects and dynamics can be probed using MD,^222−226^ but such simulations may also suffer from the pronounced finite-size effects, especially if *ab initio* MD is used. The MC simulations are capable of simulating large many-particle systems and temperature effects, but usually the atomistic picture must be simplified to a coarse-grained model based on the effective model Hamiltonian formalism.^164,213,227−229^ Recently, significant attention was also concentrated on the machine-learning-augmented calculations, which are capable of predicting new structures, phase transitions, and properties of (mixed) lead halide perovskites, while overcoming the need for large-scale simulations.^205,206,230−237^

## 2 Structural Phase Transitions in Nonmixed 3D Lead Halide Perovskites

**Figure 3:**
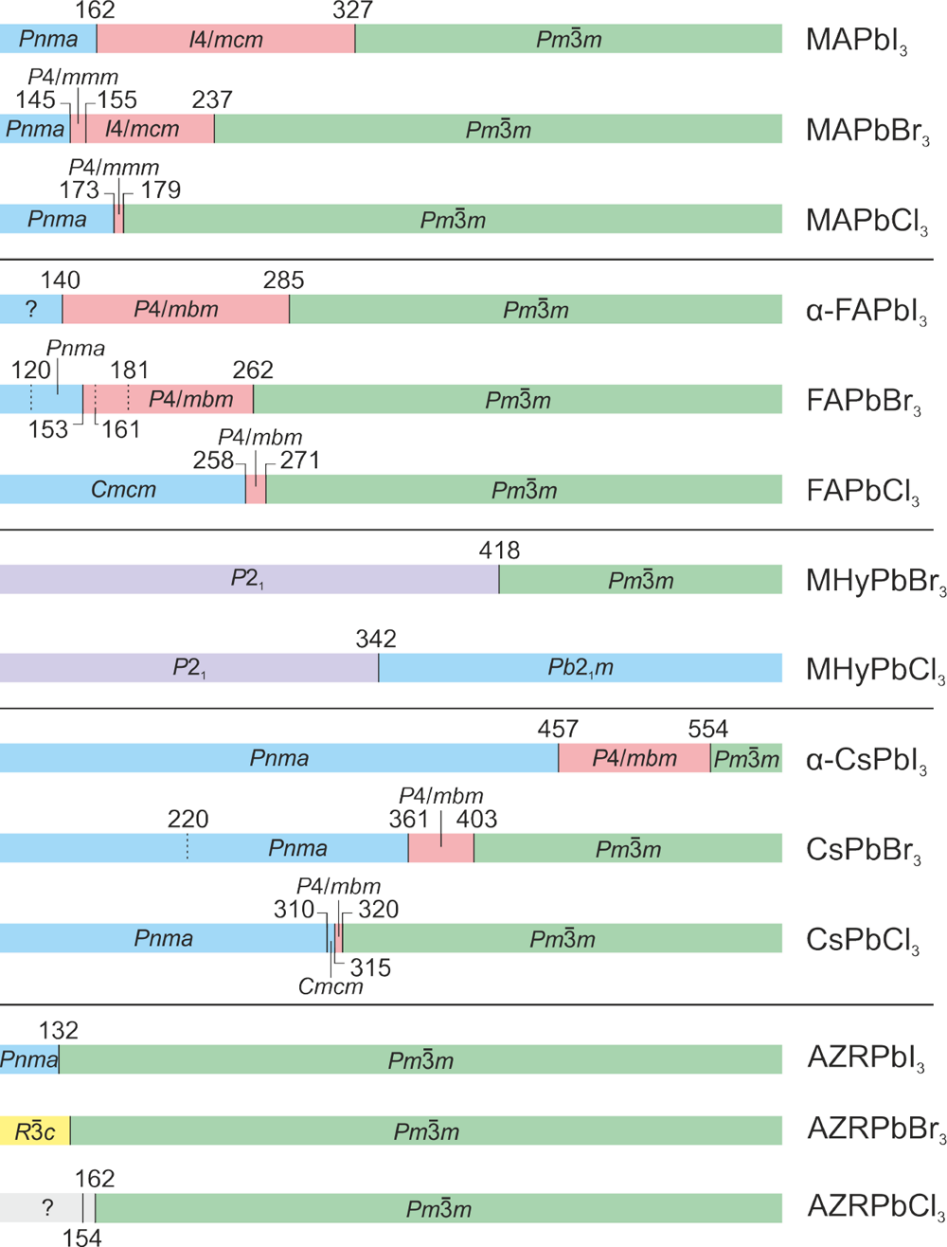
Summary of the structural phase transitions and symmetries of different 3D lead halide perovskites. Color coding: green - cubic, pink - tetragonal, blue - orthorhombic, yellow - trigonal, violet - monoclinic, gray - unknown.

Here, we discuss the structural phase transitions and phase symmetries of MAPbX_3_, FAPbX_3_, CsPbX_3_, and MHyPbX_3_ lead halide perovskites forming 3D structures. The commonly accepted phase diagrams of these compounds are summarized in Figure 3.

### 2.1 **MAPbI**_**3**_

MAPbI_3_ exhibits two structural phase transitions at 327 and 162 K associated with the symmetry lowering from the cubic  phase to tetragonal and orthorhombic symmetries. The former transition is similar to inorganic SrTiO_3_^238^ and is being driven by out of phase octahedral rotation around the cubic *c*-axis (Glazer notation:^239^*a*^0^*a*^0^*c*^–^) followed by a partial MA cation ordering and transformation of the unit cell to body centered **b** – **a**, **a** + **b**, 2**c** superstructure. The second phase transition results in a complete ordering and orthorhombic phase with more complex tilting and distortion of the PbI_6_ octahedra. Consensus has been reached in the literature regarding the change of the crystallographic system of MAPbI_3_, yet the assignment of the tetragonal and orthorhombic space group remains a topic of debate. Initially, the orthorhombic phase was modeled in the *Pna*2_1_ polar space group based on low-temperature Guinier data.^240^ However, more recent powder neutron diffraction experiments have led to its reclassification to centrosymmetric *Pnma*.^167,241^ Similarly, the tetragonal phase was originally discovered in the polar *I*4*cm* space group^93^ and later recalculated in the centrosymmetric *I*4/*mcm* space group.^242,243^ The commonly accepted centrosymmetric models of the room-temperature tetragonal and low-temperature orthorhombic phases have been called into question by numerous studies claiming ferroelectric behavior of MAPbI_3_,^53,244−248^ although no solid proof of ferroelectricity (and thus absence of centrosymmetry) was observed. Contrary, many studies claim nonferroelectric ordering in this compound,^183,227,249−252^ supporting the centrosymmetric symmetry.

Several studies also revealed the presence of octahedral rotational instabilities in the cubic phase of MAPbI_3_, which correlate with the MA cation motion and cause a local dynamic symmetry breaking.^178,179,253−258^ Beecher et al.^179^ estimated that the phonon lifetimes associated with such instabilities is on the ps time scale, and the size of such correlated domains is a few nm. Recently, Weadock et al.^178^ observed that such local structures are two-dimensional circular regions of dynamically tilted PbI_6_ octahedra that induce longer-range intermolecular MA correlations. Such lattice fluctuations cannot be observed using standard XRD techniques, which are sensitive to long-range order and thus provide an averaged-out cubic symmetry. The presence of such low-symmetry correlated octahedral distortions on the nanoscale may be related to the observed transient ferroelectric-like effects in this compound.^245−248^ Note, however, that such a dynamic picture of the tetragonal domains in the cubic phase of MAPbI_3_ was questioned in a high-resolution neutron inelastic scattering study, which instead found that the phonon scattering is consistent with the central peak phenomenon observed in classical inorganic perovskites.^259^

### 2.2 **MAPbBr_3_**

The bromide analogue MAPbBr_3_ exhibits an even more intricate phase situation than MAPbI_3_. Poglitsch and Weber^240^ reported four different phases, starting from the cubic *Pm*3̅*m*, tetragonal, *I*4/*mcm*, at 234 K, tetragonal, *P*4/*mmm*, at 155 K and finally orthorhombic with polar *Pna*2_1_ space group below 145 K. This sequence was further confirmed by the heat capacity studies.^182^ Other studies have suggested an incommensurate intermediate phase between the tetragonal and orthorhombic structures,^260^ or that only two phase transitions occur, with a phase sequence of *Pm*3̅*m* - *I*4/*mcm* - *Pnma* analogous to MAPbI_3_.^168^ The assignment of the centrosymmetric space groups is supported by the absence of a clear proof of ferroelectricity in this compound.

### 2.3 **MAPbCl_3_**

Hybrid MAPbCl_3_ also displays a diverse range of phase transitions. At room temperature, as iodide and bromide analogues, it adopts a simple cubic *Pm*3̅*m* structure, which transforms into a tetragonally distorted unit cell of *P*4/*mmm* symmetry, which exists within a narrow temperature range between 173 and 179 K. Below 173 K, the orthorhombic phase is stabilized. The *P*222_1_ space group was proposed for this phase, with a doubled *c* lattice parameter,^240^ while a *P*2_1_2_1_2_1_ symmetry was observed in a 2**a**, 2**b**, 2**c** modulated superstructure in ref (261). More recently, the *Pnma* space group was reported based on the synchrotron radiation data indicating heavily distorted PbCl_6_ octahedra.^262^ The low-temperature structure of MAPbCl_3_ appears to adopt the *a*^0^*b*^+^*c*^–^ tilt system.

### 2.4 **FAPbI**_**3**_

An even more complex behavior of the structural phase transitions is observed in the FA-based hybrid perovskites. In contrast to MAPbI_3_, the FAPbI_3_ system crystallizes in the hexagonal photoinactive yellow phase (δ-FAPbI_3_), which can be transformed into the desirable metastable photoactive black phase (α-FAPbI_3_) by thermal annealing above 150^◦^ C.^222,263^ When growing from solution media, the black phase is favored above 60 °C.^93^ Chen et al.^264^ showed that the black to yellow phase transition is a thermally activated process between the two structures separated by an energy barrier. As a result, after quenching from 400 to 200 K over 80 min, FAPbI_3_ was kinetically trapped in the black cubic phase and remained in this phase upon further cooling to low temperatures. Neutron and X-ray powder diffraction experiments^265^ conducted on both N-deuterated and hydrogenous α-FAPbI_3_ revealed that this material exhibits two phase transitions. At 285 K, the systems undergo a phase transition from the cubic  to the intermediate tetragonal centrosymmetric β-phase with a primitive tetragonal **b** – **a**, **a** + **b**, **c** unit cell, characterized by in-phase rotations (*a*^0^*a*^0^*c*^+^) similar to those found in NaTaO_3_ at high temperature.^266^ Below 140 K, a low-temperature tetragonal γ-phase of polar *P*4*bm* symmetry was claimed. SCXRD confirmed the *P*4/*mbm* tetragonal symmetry of the β-phase at 200 K.^267^ The γ-phase was also reported to have the tetragonal *P*4/*mbm* space group similar to the β-phase—on the grounds of the high-resolution synchrotron diffraction data, testifying the isostructural phase transition from the β- to γ-phase.^268^ Recent neutron powder diffraction experiments^222^ have indicated that the γ-phase is locally disordered and that there is no long-range ordering of the FA cations. The disordered γ-phase is characterized by the geometrical frustration of the molecule-cage interaction, which seems to cause a glassy behavior at low temperatures.^209^

### 2.5 **FAPbBr**_**3**_

The bromide analogue FAPbBr_3_ exhibits less complex phase behavior with the high-temperature cubic *Pm*3̅*m* (above 262 K), intermediate tetragonal *P*4/*mbm* and low-temperature orthorhombic *Pnma* structure (below 153 K) determined through combined neutron powder diffraction and synchrotron X-ray diffraction.^269^ These phase transitions are associated with partial and complete ordering of FA cations, which introduce lattice deformations. In addition, three additional isosymmetric phase transitions, which are not resolved crystallographically, were also observed in this system at 182, 162, and 118 K.^165,270^ According to QENS and Raman scattering, the isosymmetric phase transitions relate to changes in the FA cation dynamics, which may affect the optoelectronic properties of this material.^270,271^ Note that isosymmetric transitions are also observed in CsPbBr_3_^191^ and GAPbI_3_,^272^ while in MAPbBr_3_ it can be induced by applied pressure.^273^

### 2.6 **FAPbCl**_**3**_

The FAPbCl_3_ analogue has two first-order phase transitions at about 258 and 271 K. The first transition is associated with the cubic  to tetragonal symmetry lowering, while the low temperature phase was solved in the orthorhombic centrosymmetric *Cmcm* symmetry based on SCXRD data.^274^ The recent neutron diffraction studies show that in the low-temperature orthorhombic phase, FA cations undergo a 2-fold jump reorientation about the C–H axis, which changes to an isotropic rotation in the higher temperature tetragonal and cubic phases.^275^

### 2.7 **CsPbI**_**3**_

The first reports on CsPbI_3_ crystal structure appeared in the late fifties;^276^ however, the complete picture on the temperature phase behavior of CsPbI_3_ has been documented quite recently by synchrotron powder diffraction.^277,278^ The results are consistent with the formation of two low-temperature phases related to the high-temperature cubic aristo phase of α-CsPbI_3_. The first transition to a primitive tetragonal cell of *P*4/*mbm* occurs at about 554 K. On further cooling below 457 K, the orthorhombic CsPbI_3_ phase of *Pnma* symmetry is stabilized. The crystal structure of CsPbI_3_ has been also reported in refs (180, 279−281). It is noteworthy that the photoactive black phase of CsPbI_3_ is formed at high temperature through the reconstructive phase transition from a nonperovskite yellow δ-phase, which takes place slightly above 300 °C.^276^ The reconversion of the black phase to the initial yellow phase is catalyzed by humidity, and therefore, when the black phase is not exposed to humidity, it remains stable at room temperature for a matter of weeks, while in a humid environment, it transforms to the yellow phase within minutes.^107^

Similarly to hybrid lead halide perovskites, multiple studies have found that inorganic CsPbX_3_ perovskites also exhibit short-ranged dynamic fluctuations of the PbI_6_ octahedra showing that this behavior is intrinsic to the general lead-halide perovskite structure and not unique to the dipolar organic cation.^181,257,258,282^ Lanigan-Atkins et al.^181^ found that such correlated dynamics occur over a ps time scale and involve liquid-like damping of low-energy Br-dominated phonons, which correspond to 2D sheets of correlated octahedral rotations. In addition, a recent study^283^ revealed that the transformation from the yellow to the black phase of CsPbI_3_ is driven by the harmonic phonon entropy contribution to the Gibbs free energy, which is substantially higher in the softer black phase of CsPbI_3_ compared to the stiffer yellow phase. The difference in the vibrational entropy arises from the differently stacked configurations of PbI_6_ octahedra in both phases, which permit larger amplitudes of atomic vibrations in the black phase. Note that similar entropy arguments were used to explain the yellow to black phase transformation in the hybrid FAPbI_3_ system.^264^

### 2.8 **CsPbBr**_**3**_

A similar phase transition pathway is present in CsPbBr_3_, where the cubic aristo phase initially distorts to the tetragonal phase at 403 K, followed by a further symmetry decrease to the orthorhombic phase at 361 K.^276,284−288^

### 2.9 **CsPbCl**_**3**_

CsPbCl_3_ also has the cubic perovskite structure above 320 K^289^ and undergoes three successive phase transitions in a narrow, 10 K, temperature range.^290^ The prototypic *Pm*3̅*m* phase I transforms at 320 K to the tetragonal phase II (*P*4/*mbm*) and further at 315 and 311 K to the orthorhombic phases III (*Cmcm*) and IV (*Pnma*).^291−293^ The crystal structure of the *Pnma* phase has been confirmed among others by synchrotron powder diffraction^288,294^ and SCXRD at 200 MPa.^295^ It is worth adding that, contrary to CsPbCl_3_, the corresponding tin analogue CsSnCl_3_ can be trapped in the high-temperature cubic phase at room temperature by heating the sample to 100 °C and avoiding exposure to humidity.^296,297^

### 2.10 **MHyPbX**_**3**_

In contrast to lead halides templated by MA and FA cations, perovskites based on MHy crystallize into well-ordered, low-symmetry structures. MHyPbBr_3_, for instance, adopts the polar monoclinic *P*2_1_ space group, which transforms to a disordered cubic structure  above 418 K.^185^ Similarly, the chloride analogue crystallizes in the same monoclinic *P*2_1_ symmetry, which evolves to the orthorhombic polar structure, *Pb*2_1_*m*, at 342 K.^184^ Notably, the high-temperature phase is still ordered, and the contribution to spontaneous polarization from MHy dipoles is even larger than at room temperature, leading to stronger second-harmonic generation (SHG) in the high-temperature phase. It is worth highlighting that the MHy-based 3D perovskite structures have a tolerance factor greater than 1. This unique feature results from a significant distortion of the PbX_6_ octahedra by MHy, which enters the coordination sphere of lead and forms Pb–N covalent coordinate bonds. Note, however, that despite possessing a similar tolerance factor, no one has succeeded in growing a 3D MHyPbI_3_ analogue to date.

### 2.11 **AZRPbX**_**3**_

Recently, the family of 3D hybrid perovskites expanded, as new members based on cyclic aziridinium cations were synthesized.^298^ Similarly to MA and FA analogues, it was found that AZRPbX_3_ compounds exhibit a cubic *Pm*3̅*m* symmetry at room temperature. A subsequent phase transition study reported by us^237^ revealed that AZRPbI_3_ exhibits a single structural phase transition at 132 K to orthorhombic *Pnma* symmetry similar to the orthorhombic polymorph of MAPbI_3_. A single phase transition was also observed for AZRPbBr_3_ at 145 K, but instead of the orthorhombic symmetry, an unusual trigonal *R*3̅*c* space group was obtained. The chloride analogue was found to exhibit two closely spaced phase transitions at 162 and 154 K, but the low-temperature symmetries were not determined due to complex twinning of the crystal structure.

## 3 Structural Phase Transitions in Lead Halide Perovskites Comprising Two Cations

Here, we briefly discuss the structural phase transitions in pure RP, DJ, and ACI lead halides, which contain only a single cation at the A-, A′-, and A″-sites. In contrast to 3D APbX_3_ compounds, many of these structures exhibit clear ferroelectric or antiferroelectric ordering evident by the appearance of spontaneous electric polarization.

### 3.1 Ruddlesden–Popper Phases (*n* ≥ 2)

**Figure 4:**
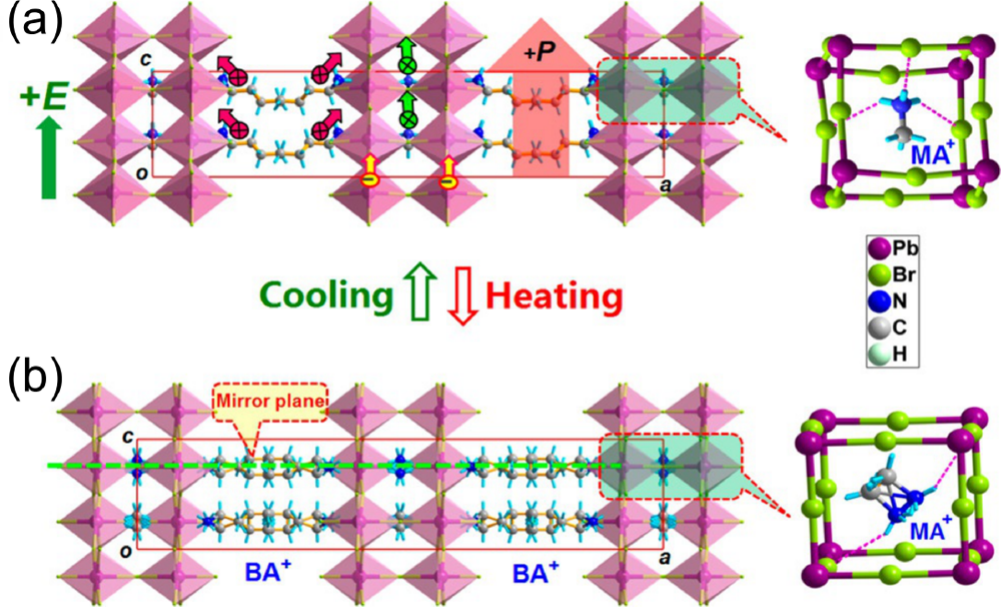
(a) Low-temperature (ferroelectric) structure of BA_2_MAPb_2_Br_7_ viewed along the *b*-axis. Red arrow denotes the direction of electric polarization. (b) High-temperature (paraelectric) structure of BA_2_MAPb_2_Br_7_. Reprinted with permission from ref (300). Copyright 2019 American Chemical Society.

Temperature-dependent structural phase transitions were reported for many RP lead halide phases of the general formula A′_2_A_*n*-1_Pb_*n*_X_3*n*+1_. Here, A′ denotes a spacer cation such benzylammonium (BZA), isobutylammonium (i-BA), EA, amylammonium (AM), isoamylammonium (i-AM), propylammonium (PA), isopropylammonium (i-PA), 3-bromopropylammonium (BPA), 3-chloropropylammonium (CPA), 4-aminomethyl-1-cyclohexanecarboxylate (t-ACH), allylammonium (AA), cyclohexanemethylammonium (MACH), 4-iodobutylammonium (4IBA), and CH_3_(CH_2_)_*n*_NH_3_^+^ (*n* = 6, 7, 8, 11, 13, 15, 17), while A is a “perovskitizer” cation such as MA, Cs, FA, EA, DMA, GA, MHy, and FA (Figure 1b).^47,130,133,134,299−302,302−330^ In contrast to 3D APbX_3_ lead halides,^183,227^ many of the discovered RP compounds, especially bromides, exhibit ferroelectric or antiferroelectric ordering.^130,133,134,300,301,303−316,320−326,328−331^Table 2 lists RP lead halides, for which the ferroelectric or antiferroelectric phase was confirmed by observation of hysteresis loops or a pyrocurrent. The electric dipole order is usually related to ordering of the bulky A′ organic cations^301,313,322,326,329,331^ or to the synergic effect of both A′ and A cation ordering and distortion of the framework.^130,133,300,303−312,314,315,320,321,323−325,328,330^ As an illustration of this process, we briefly discuss the ferroelectric phase transition in BA_2_MAPb_2_Br_7_. Li et al. showed that, above 352 K, this compound crystallizes in the centrosymmetric structure (space group *Cmca*).^300^ In this phase, the organic MA and BA cations are highly disordered, which causes cancelation of the molecular dipole moments (Figure 4a). When the temperature decreases, the organic cations order, and the crystal symmetry changes to *Cmc*2_1_. The electric polarization appears along the *c*-direction due to a specific reorientation of the organic cations (Figure 4b).^300^

**Table 2:** List of Ferroelectric (FE) and Antiferroelectric (AFE) RP Phases

compound	ordering, transition temperature (K)	symmetry change	Reference
BA_2_MAPb_2_Br_7_	FE, 352	*Cmc*2_1_ → *Cmca*	(300)
BA_2_MA_2_Pb_3_Br_10_	FE, 315	*Cmc*2_1_ → *Cmca*	(303)
BA_2_FAPb_2_Br_7_	FE, 322	*Cmc*2_1_ → *Cmcm*	(301)
BA_2_FAPb_2_I_7_	FE, 304	*Fmm*2 → *I*4/*mmm*	(314)
BA_2_CsPb_2_Br_7_	FE, 412	*Cmc*2_1_ → *Cmca*	(331)
BA_2_EA_2_Pb_3_Br_10_	FE, 380	*Cmc*2_1_ → *I*4/*mmm*	(305)
BA_2_EA_2_Pb_3_I_10_	AFE, 363; FE, 322	*Cmc*2_1_ → *Pbca* → *I*4/*mmm*	(133), (134)
i-BA_2_CsPb_2_Br_7_	AFE, 353	*Pmnb* → *Cmca*	(304)
i-BA_2_EAPb_2_Br_7_	FE, 326	*Cc* → *I*4/*mmm*	(313)
i-BA_2_EA_2_Pb_3_Br_10_	FE, 370	*Cmc*2_1_ → *I*4/*mmm*	(313)
i-BA_2_FAPb_2_Br_7_	AFE, 303.2	*Pnma* → *I*4/*mmm*	(315)
i-BA_2_MAPb_2_Cl_7_	FE, 316	*Fmm*2 → *I*4/*mmm*	(320)
EA_4_Pb_3_Cl_10_	FE, 415	*Cmc*2_1_ → *I*4/*mmm*	(310)
EA_4_Pb_3_Br_10_	FE, 384	*C*2*cb* → *I*4/*mmm*	(306)
EA_2_MA_2_Pb_3_Br_10_	FE, 375	*Cmc*2_1_ → *I*4/*mmm*	(311)
EA_2_MA_2_Pb_3_Cl_10_	FE, 390	*Cmc*2_1_ → *I*4/*mmm*	(321)
AM_2_CsPb_2_Br_7_	FE, 313 and 349	*F*222 → *Cmc*2_1_ → *I*4/*mmm*	(322)
AM_2_EA_2_Pb_3_I_10_	FE, 361 and 398	*Pmc*2_1_ → *Cmce* → *I*4/*mmm*	(330)
i-AM_2_CsPb_2_Br_7_	FE, 323 and 349	*Cmc*2_1_ → *Pbcm* → *I*4/*mmm*	(329)
i-AM_2_Cs_3_Pb_4_Br_13_	FE, 351 and 365	*Cmc*2_1_ → *I*4/*m* → *I*4/*mmm*	(326)
i-AM_2_EA_2_Pb_3_Br_10_	FE, 371	*Cmc*2_1_ → *I*4/*mmm*	(307)
i-AM_2_EA_2_Pb_3_Cl_10_	FE, 392	*Fmm*2 → *I*4/*mmm*	(324)
-AM_2_EA_2_Pb_3_I_10_	FE, 313; AFE, 340	*Cmc*2_1_ → *Pmcn* → *I*4/*mmm*	(309)
i-AM_2_MA_2_Pb_3_Br_10_	FE, 305	*Cmc*2_1_ → *I*4/*mmm*	(323)
i-AM_2_MA_2_Pb_3_Cl_10_	FE, 343 K	*Pmc*2_1_ → *I*4/*mmm*	(325)
PA_2_FAPb_2_Br_7_	FE, 263.3	*Cmc*2_1_ → *Cmcm*	(130)
BPA_2_FAPb_2_Br_7_	FE, 348.5	*Cmc*2_1_ → *Cmcm*	(130)
CPA_2_FAPb_2_Br_7_	FE, 335 K	*Cmc*2_1_ → *Fmmm*	(328)
t-ACH_2_EA_2_Pb_3_Br_10_	FE, 355 and 380	*Pm* → *Pmmn* → *Pmmn*	(308)
AA_2_EA_2_Pb_3_Br_10_	FE, 378	*Cmc*2_1_ → *I*4/*mmm*	(312)
C6_2_Pb_2_Br_7_	FE, 248, 306	? → *Cc* → *C*2/*c*	(316)
C7_2_Pb_2_Br_7_	FE, 260	? → *Cc*	(316)
C7_2_Pb_2_Br_7_	FE, 261, 275	? → ? → *Cc*	(316)

### 3.2 Dion-Jacobson Phases (*n* ≥ 2)

In contrast to the RP compounds, temperature-dependent structural phase transitions were reported only for two DJ lead halide perovskites: (AMP)(MA)Pb_2_I_7_, (where AMP = 4-(aminomethyl)piperidinium), which undergoes two order–disorder phase transitions at 367 and 449 K,^131^ and (BDA)(EA)Pb_3_Br_10_ (BDA = 1,4-butyldiammonium), which also shows the presence of two phase transitions at 363 and 397 K.^332^ (AMP)(MA)Pb_2_I_7_ exhibits ferroelectric properties, and the polar order is preserved up to the decomposition temperature,^131^ while (BDA)(EA)Pb_3_Br_10_ undergoes ferroelectric-ferroelectric and ferroelectric-paraelectric phase transitions associated with the *Pmn*2_1_ → *Ama*2 → *Cmcm* symmetry change.^332^ Similarly to the RP phases, the electric order in these compounds appears due to the ordering of organic cations.

### 3.3 ACI Perovskites

In this family of compounds, structural phase transitions were reported for (TR)(GA)PbBr_4_ (*P*2_1_/*c* → *P*2/*c*)^44^ and (IM)(MHy)PbX_4_ (*P*2_1_/*c* → *P*2/*c*, X = Br, Cl).^333^ In both cases, the phase transitions were found to be of the order–disorder type. However, in the case of (TR)(GA)PbBr_4_, the change of the space group from *P*2/*c* to *P*2_1_/*c* occurs due to the ordering of the intralayer TR cations, whereas in (IM)(MHy)PbBr_4_ and (IM)(MHy)PbCl_4_ both organic cations are disordered in the high-temperature phases and well-ordered at low temperature.

## 4 A-Site Mixing in 3D Perovskites

We now will start discussing the mixing effects in 3D lead halide perovskites by considering the most popular mixing recipe, which involves cation substitution at the A-site. We discuss how this mixing affects the structural and dynamic properties of these materials. The cubic 3D perovskite structure is predicted to be most stable for the tolerance factor close to 1, while for values significantly lower than 1 a lower symmetry can be expected at room temperature. For instance, MAPbI_3_ with a tolerance factor of 0.912^334^ crystallizes in the tetragonal *I*4/*mcm* symmetry at room temperature. An effective way to increase the tolerance factor and stabilize the cubic phase is A-site mixing with larger cations. However, the extent of mixing highly depends on the size of the guest cation—a significant mismatch in the cation size results in a solubility limit, beyond which the phase separation typically occurs.

### 4.1 MA_1–*x*_FA_*x*_PbX_3_

Mixing of MA and FA cations is among the most popular recipes to enhance the performance and stability of lead halide perovskites.^115,335^ Similar radii of the cations (217 and 253 pm, Table 1)^15^ and the fact that the end members have perovskite architectures result in the 3D perovskite structures for all mixing concentrations of MA_1–*x*_FA_*x*_PbX_3_ (no solubility limit) providing an excellent system for a complete mapping of the phase diagram.

#### 4.1.1 **MA**_**1–***x*_**FA**_***x***_**PbI**_**3**_

**Figure 5:**
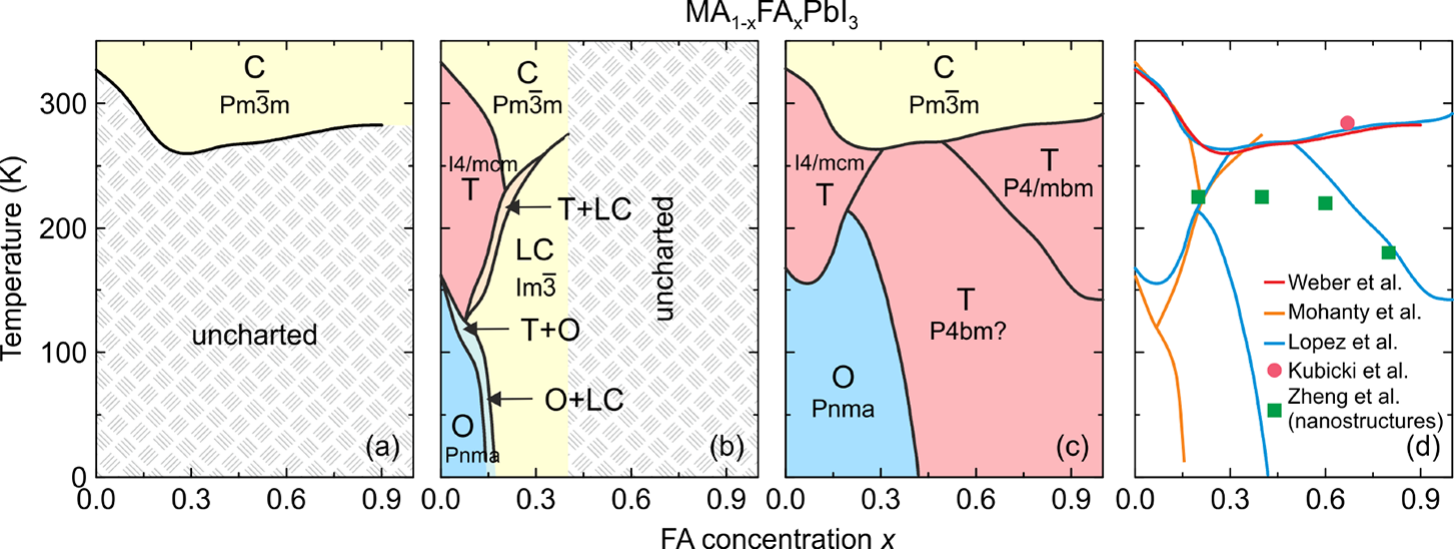
(a–c) Temperature–composition phase diagrams of a mixed MA_1–*x*_FA_*x*_PbI_3_ system obtained in different studies. Symmetry notation: C - cubic, LC - large-cell cubic, T - tetragonal, O - orthorhombic. (a) Adapted with permission from ref (162). Copyright 2016 Royal Society of Chemistry. (b) Adapted with permission from ref (336). Copyright 2019 American Chemical Society. (c) Adapted with permission from ref (88). Copyright 2020 American Chemical Society. (d) Comparison of the phase boundaries presented in (a–c) together with the added data points from refs (102) and (337).

The first work studying mixing effects on the phase behavior in the MA_1–*x*_FA_*x*_PbI_3_ system was reported by Weber et al.,^162^ where the high-temperature phase transition was investigated using SCXRD and PXRD methods. In the low *x* region, a substantial lowering of the phase transition temperature was observed upon mixing, reaching a minimum value of about 260 K for *x* = 0.3 (Figure 5a). For higher values of *x*, the phase transition temperature recovered, as the system evolved from the MA-rich state to the FA-rich composition. The space group of the cubic phase was assigned to *Pm*3̅*m*, which is in agreement with parent MAPbI_3_ and α-FAPbI_3_ compounds (Figure 3), while the XRD data below the phase transition point were indexed using either doubled cubic or tetragonal unit cell.

A following study from the Sarma group^336^ reported a detailed XRD characterization and dielectric properties of the MA_1–*x*_FA_*x*_PbI_3_ system in the MA-rich region of the phase diagram (*x* ≤ 0.4) and a broad temperature range (Figure 5b). For very low values of mixing (*x* < 0.07), the authors observed a typical cubic-tetragonal-orthorhombic symmetry lowering inherited from MAPbI_3_ perovskite. The mixing also caused the decrease of the phase transition temperatures compared to the MAPbI_3_ system in agreement with the aforementioned study by Weber et al.^162^ At the intermediate values of the studied compositions (0.07 < *x* < 0.2), the authors claim an observation of a cubic super cell (*Im*3̅ space group) at low temperature instead of the orthorhombic phase, which demonstrated an unusual reentrant cubic-tetragonal-cubic behavior. For *x* > 0.2, the tetragonal phase also becomes completely suppressed at the expense of the growing boundary of this large-cell cubic phase. Eventually, only the transition between the cubic and large-cell cubic phases is observed. The authors also note that the XRD data indicate narrow phase coexistence regions at the phase boundaries as depicted in Figure 5b.

The whole FA concentration range in the MA_1–*x*_FA_*x*_PbI_3_ system was recently explored by Francisco-López et al.^88^ using the temperature-dependent Raman and PL spectroscopies. The authors relied on the available structural information on MAPbI_3_ and α-FAPbI_3_ perovskites to construct a complete phase diagram of the mixed system (Figure 5c). In the FA-rich region (*x* > 0.5), the authors show the presence of the tetragonal *P*4/*mbm* phase inherited from α-FAPbI_3_. In addition, it is claimed that a tetragonal-tetragonal phase transition exhibits a critical point at *x* = 0.7, as the evolution of the PL peak shows no discontinuity with decreasing temperature. However, this assignment is debatable, as the sharp transition anomaly is expected to become diffused in the highly mixed region. A comparison with the study by Weber et al.^162^ reveals an almost identical phase boundary between the cubic and tetragonal phases in the whole range of mixing (summarized in Figure 5d). However, a comparison with the phase diagram provided by the Sarma group^336^ shows that the phase boundary of the orthorhombic phase is substantially shifted to higher values of *x* (Figure 5d). This may suggest different incorporation yields of FA cations in both works, although agreement of the phase boundaries in the high temperature region is satisfactory. No conclusions regarding the discrepancy between the phase symmetries can be made, as no diffraction experiments were performed.

The tuning of the phase transition temperature by mixing was also observed in the nanostructures of MA_1–*x*_FA_*x*_PbI_3_ using temperature-dependent PL measurements.^337^ A small disagreement with other studies in transition temperatures can be observed (see Figure 5d), which may originate from a small size of the nanostructures and broadening of the anomalies in the highly mixed region.^338^

Ahmadi et al. used piezoresponse and Kelvin probe force microscopies and SHG to study the long- and short-range electric dipole and charge dynamics in MA_0.15_FA_0.85_PbI_3_ composition.^339^ Surprisingly, the authors reported indications of ferroelectric domains in this mixed compound; however, the domain dynamics were found to be suppressed by fast ion motion.

**Figure 6:**
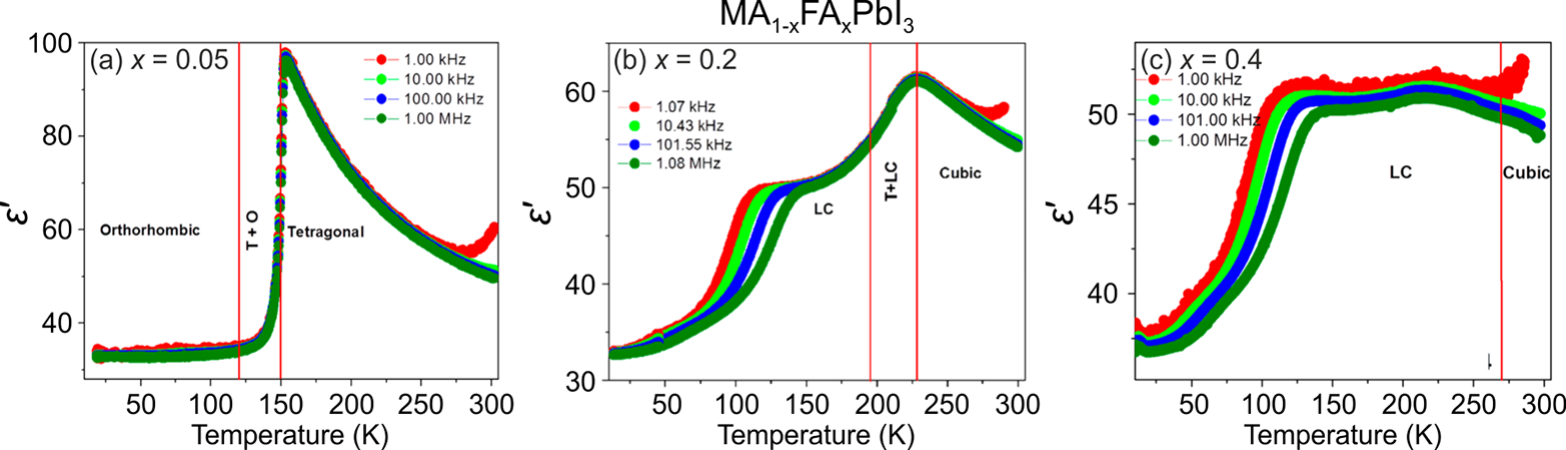
Temperature dependence of the real part of the complex dielectric permittivity ε′ of the mixed MA_1–*x*_FA_*x*_PbI_3_ pellet samples for *x* = (a) 0.05, (b) 0.2, and (c) 0.4. Reprinted with permission from ref (336). Copyright 2019 American Chemical Society.

In addition to the structural data, the aforementioned study from the Sarma group^336^ reported the temperature dependence of the real part of the complex dielectric permittivity of the MA_1–*x*_FA_*x*_PbI_3_ (*x* ≤ 0.4) pellet samples in a narrow frequency range (1 kHz - 1 MHz) (see Figure 6 for selected compositions). As pointed out by the authors, the dielectric response in this mixed system mainly originates from MA cations due to a much lower electric dipole moment of FA (2.3 D vs 0.2 D, Table 1). At a low FA concentration (Figure 6a), the main feature of the dielectric response is a sharp decrease at the tetragonal-orthorhombic phase transition point caused by the MA cation ordering, while the cubic-tetragonal phase transition is poorly visible. This behavior is in a good agreement with a number of previous DS works on pure MAPbX_3_ perovskites.^183,340,341^ Interestingly, the authors claim that the anomaly at higher values of *x* is related to the transition from the cubic to large-cell cubic phase (Figure 6b). The most intriguing feature of the dielectric response is the broad dipolar relaxation at low temperature, which appears for a higher FA content (Figure 6b,c). This relaxation extends from about 120 to 25 K and is assigned by the authors to the dipolar (orientational) glassy state, which indeed has some similarities with the classical dipolar glasses (see Figure 2c), although broadband DS experiments are necessary to make an unambiguous assignment.

A subsequent study from the same group used QENS experiments to investigate the dipolar glass phase and molecular cation dynamics of the mixed MA_0.875_FA_0.125_PbI_3_ compound in more detail.^217^ The temperature-dependent experiments allowed probing the MA and FA cation dynamics in different structural phases including the proposed large-cell cubic phase. The QENS data were best modeled using a 4-fold (*C*_4_) reorientation for the MA cation motion in the tetragonal and cubic phases with the activation energy *E*_a_ of 83 meV in a good agreement with 82 meV observed for pure MAPbI_3_. At a lower temperature, where the 3-fold rotation of MA around the C–N axis dominates, the mixed composition displayed a distribution of activation energies in contrast to pure MAPbI_3_, which showed *E*_a_ = 53 meV. This result further supports the appearance of the dipolar glass phase due to different MA cation environments caused by FA incorporation. Interestingly, the analysis of the elastic incoherent structure factor revealed that FA cations are frozen even up to 340 K, indicating they exhibit a much higher activation energy, and thus the A-site dynamics are governed by MA cations. The fraction of the mobile MA cations was found to be slightly higher in the mixed composition compared to pure MAPbI_3_, which was explained by an increase of the unit cell volume upon introduction of bigger guest cations. A fractional isotropic rotational model was used to estimate the characteristic time scales of the MA rotation at different temperatures, revealing that MA cation motion is faster in the mixed compound (e.g., 84 ps vs 188 ps at 110 K).

Similar observations and signatures of the orientational glass state were presented by Drużbicki et al.,^225^ where higher mixing compositions were investigated (*x* = 0.6 and 0.9) by the inelastic neutron scattering experiments assisted by the harmonic lattice dynamics and *ab initio* MD simulations. In this study, a highly disordered local environment of MA cations was observed, which was assigned to weakening of the H-bonds and framework expansion induced by mixing. On the other hand, an opposite behavior was found for FA cations, as they form stronger H-bonds with the distorted inorganic cage caused by mixing.

Another interesting NMR and state-of-the-art MD study was reported by Grüninger et al.,^206^ where the authors found indications of a clustering of the MA and FA cations within the mixed *x* = 0.25, 0.5, and 0.75 compounds. Such a local heterogeneous distribution would inevitably cause a local variation of the perovskite lattice and is likely related to the observation of the glassy phase in these compounds.

The MA and FA cation dynamics in the mixed deuterated MA_0.33_FA_0.67_PbI_3_ system were investigated by Kubicki et al.^102^ using solid-state ^2^H and ^14^N magic angle spinning (MAS) NMR spectroscopy, which allowed a simultaneous probing of both cations. Temperature-dependent measurements of the quadrupolar coupling constants of ^2^H and ^14^N nuclei demonstrated that the reorientation of the C–N bond in MA cations at 327 K is not fully isotropic in the mixed compound, which is in contrast to pure MAPbI_3_ perovskite. The authors also observed close to isotropic motion of FA cations in the cubic phase. As temperature decreased, this motion slowed down and froze, while a substantial rotation of the N–D_3_ and N–D_2_ groups around the C–N bonds of FA remained even at 100 K. At room temperature, it was found that FA rotates much faster than MA (12 ps vs 133 ps), which was attributed differences in the H-bond strength between the cations and the framework. The authors also correlated much faster FA rotation to increased charge carrier lifetimes observed in FAPbI_3_ and mixed MA_1–*x*_FA_*x*_PbI_3_ systems compared to pure MAPbI_3_.^102^ In addition to the cation dynamics, this study also provided the phase transition temperature to the cubic phase of 285 K in a good agreement with other studies (Figure 5d). It is interesting to compare the reported results with the aforementioned QENS study.^217^ Both works detected that mixing alters the MA cation motion, while the observations regarding the FA cation dynamics are in sharp contrast, as the Sarma group reported that the rotational dynamics of FA cations are entirely suppressed over the entire temperature range in the *x* = 0.125 compound. The observed differences may be explained by different studied compositions, suggesting that level of mixing can highly influence the FA cation dynamics.

A recent NMR quadrupolar relaxation study^204^ from the same group indicated that the FA cation dynamics in the cubic phase of MA_0.3_FA_0.7_PbI_3_ is essentially the same as in FAPbI_3_ compound and occur on about a 1 ps time scale with activation energy close to 100 meV. On the other hand, the authors reported that the MA cation motion is about 2× faster in the mixed compound compared to MAPbI_3_ (1.0 ps vs 1.95 ps). Note that the reported cation correlation times are about 1–2 orders of magnitude shorter compared to their previous work,^102^ which was explained by difficulties in relating the measured ^14^N line widths to the motionally induced transverse relaxation, whereas quadrupolar relaxation measurements are much more reliable. Interestingly, the authors observed that the activation energy of the MA cation motion was not affected by mixing and remained close to 150 meV. Note that Fabini et al.^209^ also found very similar MA and FA cation rotation rates in nonmixed MAPbI_3_ and FAPbI_3_ compounds. The observation of fast FA cation dynamics in the *x* = 0.7 compound is in a sharp contrast to the aforementioned QENS results obtained by the Sarma group,^217^ where frozen FA cations were observed for the *x* = 0.125 composition. This discrepancy may originate from vastly different fractions of FA cations in the studied compounds, which belong to the opposite sides of the phase diagram.

#### 4.1.2 **MA**_**1–*****x***_**FA**_***x***_**PbBr**_**3**_

**Figure 7:**
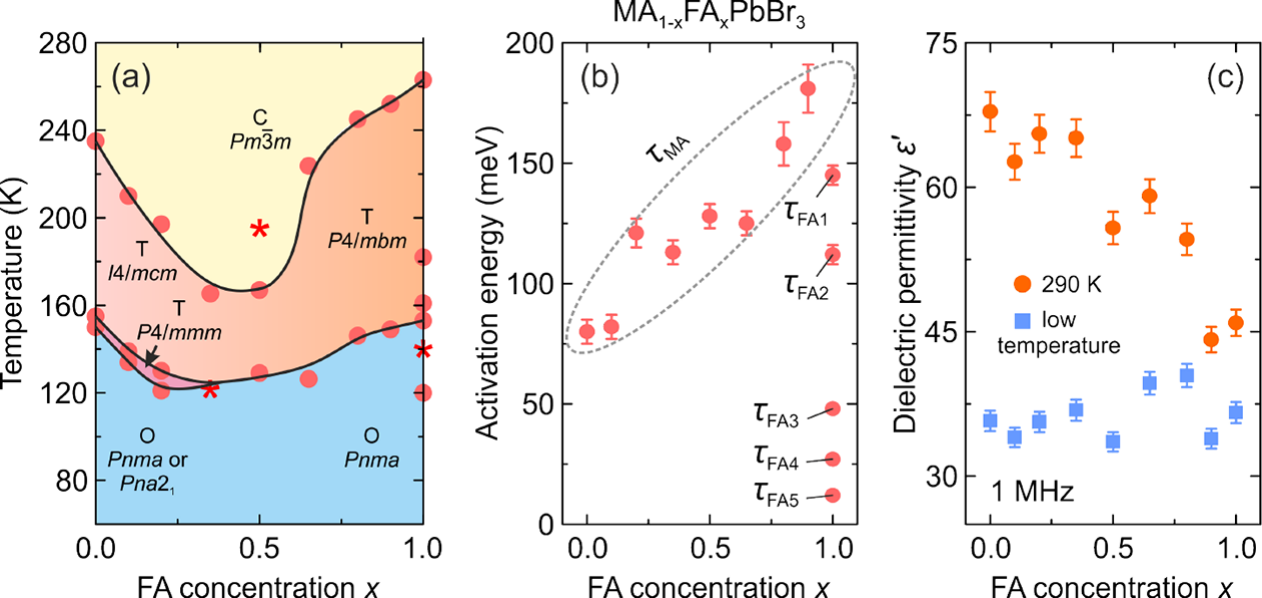
(a) Temperature–composition phase diagram of the mixed MA_1–*x*_FA_*x*_PbBr_3_ system. Symmetry notation: C - cubic, T - tetragonal, O - orthorhombic. The phase boundary between the tetragonal *I*4/*mcm* and *P*4/*mbm* phases is unknown and thus not indicated in the phase diagram. FA concentration dependence of the (b) activation energy of the dipolar relaxations and (c) room-temperature and low-temperature dielectric permittivity (1 MHz). Adapted with permission from ref (165). Copyright 2021 American Chemical Society.

Recently, we used a multitechnique approach (DSC, heat capacity, ultrasonic measurements, SCXRD, broadband DS, Raman spectroscopy) to thoroughly investigate the mixing effects on the structural phase transition, dielectric response, and cation dynamics in the bromide analogue of the mixed MA_1–*x*_FA_*x*_PbBr_3_ (0 ≤ *x* ≤ 1) system.^165^ A suite of different experimental techniques probing structural phase transitions of single crystal samples allowed us to construct the phase diagram for the whole range of FA concentrations (Figure 7a). The obtained phase diagram shows lowering of the phase transition temperatures upon mixing and thus stabilization of the desirable cubic phase.^122^ We also observed disappearance of the intermediate tetragonal phase upon mixing in the MA-rich region of the phase diagram. On the other limit of mixing, the three isosymmetric transitions of FAPbBr_3_^172,270^ were also fully suppressed even at the lowest studied MA content, which may paradoxically be related to a relief of the FA cation frustration by mixing (see ref (172) for details).

A comparison of the phase diagrams of the MA_1–*x*_FA_*x*_PbBr_3_ and MA_1–*x*_FA_*x*_PbI_3_ systems shows a qualitative agreement for low values of *x* (see Figures 5d and Figure 7a). However, the central parts of the diagrams are significantly different, as higher symmetry phases were found at low temperature for the MA_1–*x*_FA_*x*_PbI_3_ perovskites (see Figure 5).^88,336^ We note that such differences between both systems are expected due to different phase behaviors of the iodide and bromide parent compounds.

**Figure 8:**
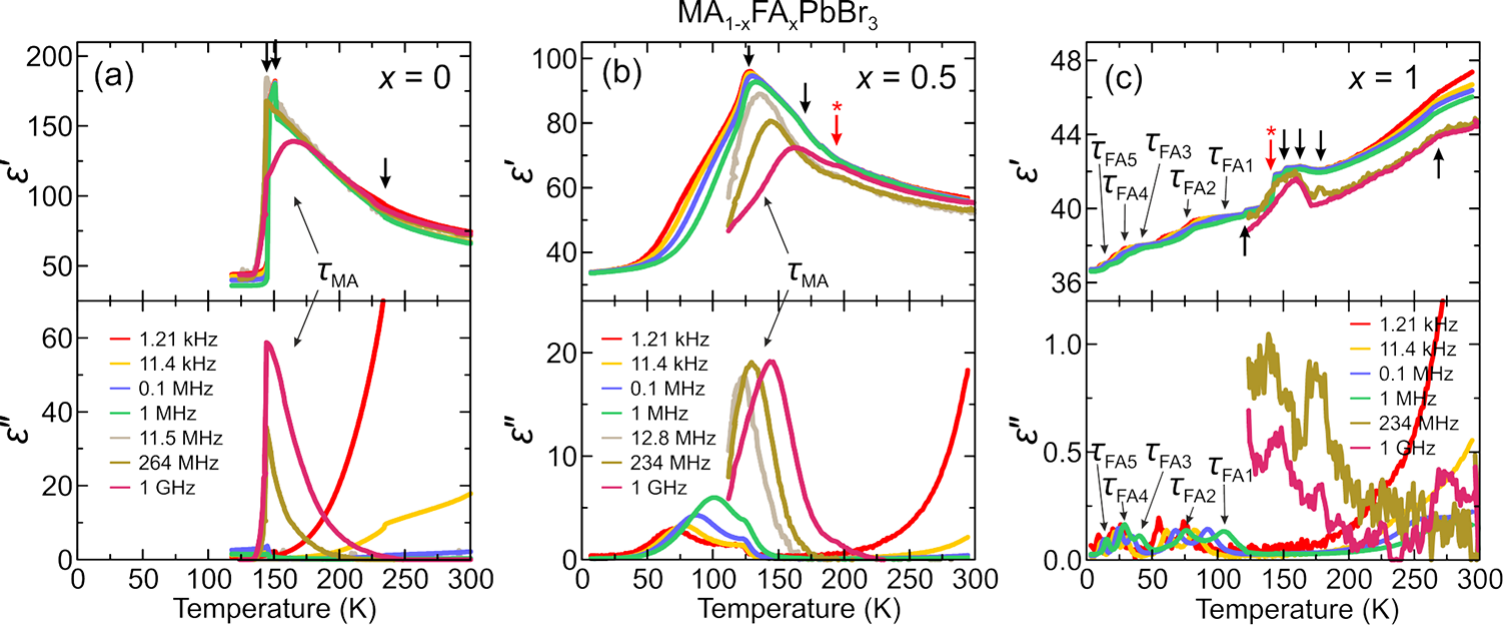
Temperature dependence of the complex dielectric permittivity of (a) MAPbBr_3_, (b) MA_0.5_FA_0.5_PbBr_3_, and (c) FAPbBr_3_ single crystals presented at selected frequencies. Arrows indicate phase transition anomalies and dielectric relaxations. Adapted with permission from ref (165). Copyright 2021 American Chemical Society.

In the same study,^165^ we also reported the temperature-dependent broadband DS experiments (Hz-GHz frequency range) of the MA_1–*x*_FA_*x*_PbBr_3_ single crystal samples. The most important results comparing the dielectric responses of the parent compounds (*x* = 0 and 1) and the highest mixing case (*x* = 0.5) are presented in Figure 8. Similarly to the iodide analogue, the complex dielectric permittivity of MAPbBr_3_ compound exhibits an anomalous decrease at the tetragonal-orthorhombic phase transition related to the establishment of the long-range MA cation order.^183,340,341^ Other phase transitions are visible as small anomalies. On the other end of the composition, the dielectric response of FAPbBr_3_ perovskite shows several weak anomalies originating from the phase transitions (see phase diagram, Figure 7a).^274^ In addition, five distinct and well-resolved dielectric relaxations of unknown origin were identified in the orthorhombic phase (best visible in the ε″ data). Interestingly, these relaxations mimic the behavior of the isosymmetric transitions, as they also disappear upon mixing even for the lowest MA concentration. The most interesting dielectric response was observed for the highly mixed MA_0.5_FA_0.5_PbBr_3_ compound, where both phase transitions (cubic-tetragonal-orthorhombic) were visible as very weak anomalies (Figure 8c). In addition, a broad dielectric dispersion of MA cations reminiscent of a glassy phase was detected at low temperature in agreement with the iodide analogue (compare with Figure 6c). A strong proof of the dipolar glass phase would be observation of the Vogel–Fulcher freezing behavior of the relaxation time associated with the observed dielectric relaxation. However, it was found that the dynamics of this process follows the typical Arrhenius law preventing an unambiguous assignment of the dipolar glass phase.

The broadband DS can directly measure how mixing affects the value of the dielectric permittivity, which is a relevant parameter influencing the performance of photovoltaic devices.^53,55,56,183,190−192^ We observed that the MA and FA cation mixing significantly affects the room-temperature dielectric permittivity of the MA_1–*x*_FA_*x*_PbBr_3_ system (Figure 7c), as ε′ showed a rather strong decrease with increasing *x*, which was assigned to the diminishing number of the MA cations.^165^ Note that the permittivity value at low temperature was not affected by mixing (Figure 7c), indicating that it is originating from the inorganic part of the lattice. As discussed by Fabini et al.,^342^ such a uniform and rather high value of ε′ is related to the lattice polarizability invoked by the 6s^2^ lone-pair electrons and associated off-centering of lead ions.

In the same study, we also proved the dynamic effects in the MA_1–*x*_FA_*x*_PbBr_3_ system using the broadband DS.^165^ The relaxation times extracted from the dipolar relaxations were approximated using the Arrhenius law providing the activation energy *E*_a_ of the MA cation motion. More than a 2-fold increase (from 80 to 180 meV) of the *E*_a_ values with increasing *x* was observed, indicating that FA cations significantly raise the rotation barrier of the MA cations (Figure 7b). The increase of the activation energy with *x* may seem to be in contradiction with the aforementioned NMR study of the related MA_0.22_FA_0.78_PbI_3_ compound,^204^ where practically no change in *E*_a_ was observed upon mixing at room temperature. However, one should note that our study probed activation energies at significantly lower temperatures, where molecular cation motion was substantially hindered. Note that the dipolar dynamics observed by the DS can be extrapolated to room temperature, revealing a 2 ps dipolar relaxation time of MA in MA_0.2_FA_0.8_PbBr_3_, which is in a very good agreement with the MA reorientation time of 1 ps obtained in MA_0.22_FA_0.78_PbI_3_ using NMR.^204^ Interestingly, the activation energies of the five low-temperature processes of FAPbBr_3_ showed a very huge spread ranging from 145 to 12 meV (Figure 7b),^165^ which may be related to the recently proposed quadrupolar geometric frustration of the FA cations.^270^

The temperature-dependent Raman data reported in the same work^165^ provided information on the degree of the molecular cation ordering. For small values of *x*, the MA cation modes exhibited significant narrowing at the transition to the orthorhombic phase, indicating ordering of the cations. In contrast, no substantial narrowing was observed at the phase transitions points in the highly mixed and FA-rich compositions (*x* ≥ 0.5) showing the absence of the MA cation ordering in agreement with the low-temperature dielectric response. For all mixed compounds, the width of the FA band decreased with decreasing temperature, indicating slowing down of the FA cation dynamics.

In a recent study, Kalita et al. investigated structural phase transitions, framework deformation, and dynamic effects in MAPbBr_3_, MA_0.5_FA_0.5_PbBr_3_, and FAPbBr_3_ compositions.^343^ The heat capacity measurements revealed that the mixed composition exhibits two structural phase transitions at 164 K (cubic-tetragonal) and 128 K (tetragonal-orthorhombic) in a very good agreement with the phase diagram reported in ref (165). Based on the synchrotron XRD measurements, the symmetry of the orthorhombic phase for the *x* = 0.5 solid solution was assigned to *P*4/*mbm*. In addition, it was observed that the Pb–Br length increases upon mixing, which was assigned to a larger radius of FA cation. It was also found that the PbBr_6_ octahedral tilting in the tetragonal phase increases with an increase of *x*, while the highest distortion of the octahedra in the orthorhombic phase was observed for the MAPbBr_3_ compound. The temperature-dependent Raman spectroscopy was employed to study the phonon modes in the low-frequency range (50 to 500 cm^–1^). In agreement with the previous study,^165^ it was found that the modes become well resolved in the orhothorhombic phase of MAPbBr_3_ due to the ordering of the system. In the mixed composition, the low-frequency modes were found to be much broader, indicating disorder compared to MAPbBr_3_.

### 4.2 MA-Based Compounds

Here, we review how mixing with less compatible A-site cations affects the phase transitions and dynamic properties of the MAPbX_3_-based lead halide perovskites. Mixing with such cations results in a solubility limit, beyond which the phase separation (mixed phase) typically occurs.

#### 4.2.1 **MA**_**1–*****x***_**Cs**_***x***_**PbBr**_**3**_

**Figure 9:**
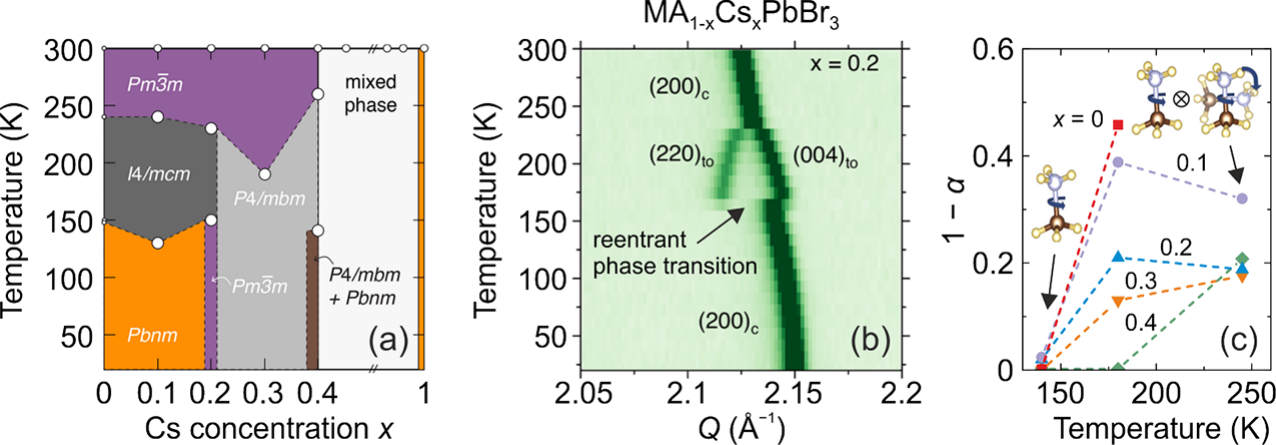
(a) Temperature–composition phase diagram of the mixed MA_1–*x*_Cs_*x*_PbBr_3_ system. Phase separation occurs for *x* ≥ 0.4. (b) Neutron powder diffraction data of the *x* = 0.2 composition showing the reentrant phase transition (arrow). (c) Fraction of the MA cations participating in the reorientation around the *C*_4_ symmetry axis (inset) as extracted from the QENS data for different Cs concentrations. Reprinted (adapted) with permission from ref (163). Copyright 2017 American Chemical Society.

Mozur et al.^163^ reported a detailed multitechnique investigation of the mixing effects in the MA_1–*x*_Cs_*x*_PbBr_3_ system. The employed advanced synchrotron and neutron diffraction methods allowed them to construct a rich phase diagram of this system (Figure 9a). Interestingly, despite both parent compounds existing in a 3D perovskite form (MAPbBr_3_ and CsPbBr_3_), the authors observed a solubility limit of Cs^+^ cations in MAPbBr_3_ (*x* ≈ 0.4). This was explained by different octahedral tilting preferences of smaller Cs^+^ cations (Table 1).

The authors also found that the *x* = 0.1 composition exhibits the same sequence of the phase transitions (cubic-tetragonal-orthorhombic) as in pure MAPbBr_3_. The situation is different for *x* = 0.2, where, instead of the tetragonal-orthorhombic transformation, a reentrant transition back to the same *Pm*3̅*m* cubic phase was observed (Figure 9b). Such a surprising behavior was explained using the modified Blume–Capel model with included strain coupling. At higher Cs fraction (*x* = 0.3), the system transitioned from the cubic symmetry to the tetragonal *P*4/*mbm* phase instead of *I*4/*mcm* observed at lower values of mixing. Note that the *P*4/*mbm* space group is also observed for pure CsPbBr_3_ compound (Figure 3), indicating that Cs^+^ ions already dictate the octahedral tilt pattern. Interestingly, the transition to the orthorhombic phase was completely suppressed for this Cs concentration. This composition also showed the maximum extent of the cubic phase reaching 200 K. The situation changed again for the *x* = 0.4 composition, where the orthorhombic phase recovered and showed coexistence with the tetragonal phase (Figure 9a).

The same study also used a suite of neutron diffraction and neutron scattering techniques to probe the MA cation arrangement and dynamics for different fractions of mixing.^163^ The neutron powder diffraction data demonstrated the absence of the preferred MA cation orientation for all values of *x* even at low temperature. This was also supported by the inelastic neutron scattering data, which provided evidence of the heterogeneous environments of both the inorganic framework and the MA cations. In addition, the authors observed an excess of low energy modes in the heat capacity data due to the increased disorder. All these findings were attributed to the orientational glass behavior present in both organic and inorganic sublattices and caused by the local-strain induced frustration. The dynamics of the MA cations in MA_1–*x*_Cs_*x*_PbBr_3_ compounds were probed using QENS. The mean squared displacement of hydrogen atoms revealed decreasing amplitude with increasing *x* and lowering of temperature. This indicated that the motion of MA cations is more hindered in the highly mixed phases. This was also supported by the measurement of the elastic incoherent structure factor, which revealed a drastic decrease in the overall MA cation reorientation around the *C*_4_ symmetry axis upon mixing (Figure 9c). Interestingly, this observation seems to be different than detected in the MA_1–*x*_FA_*x*_PbX_3_ system, where for high levels of mixing the MA cations exhibited substantial dynamics even in the orthorhombic phase as is evident from the dielectric response (Figures 6 and 8).^165,336^

#### 4.2.2 **MA**_**1–*****x***_**Cs**_***x***_**PbI**_**3**_

In a recent study, Gallop et al. used XRD, Raman, and 2D IR spectroscopies to investigate the structure and MA cation dynamics of the MA_1–*x*_Cs_*x*_PbI_3_ thin film samples.^344^ The authors concentrated on the *x* ≤ 0.3 compositions, as for higher Cs fractions signatures of the yellow phase were observed. Note that a similar solubility limit was obtained in the discussed MA_1–*x*_Cs_*x*_PbBr_3_ system.^163^ The XRD and Raman experiments indicated shrinkage of the unit cell and a continuous tilting of the octahedral framework upon mixing due to the smaller size of the Cs^+^ cation. The 2D IR spectroscopy experiments performed at room temperature revealed that the introduction of Cs^+^ makes the MA cation dynamics faster. The characteristic time of the jumping dynamics increased from about 4 ps (*x* = 0) to 12 ps (*x* = 0.3), while the increase of the wobbling dynamics was less pronounced (0.5 ps vs 0.65 ps). The obtained results were correlated with the increased *c*/*a* distortion upon mixing, which was also confirmed by the MD simulations.

#### 4.2.3 **MA**_**1–*****x***_**DMA**_***x***_**PbX**_**3**_**(X = I, Br)**

The effective radius of DMA is only slightly larger than that of FA (Table 1), making it a highly promising alternative cation for mixing in lead halide perovskites. However, pure DMAPbX_3_ compounds form hexagonal structures,^345,346^ making them only partially compatible with the 3D perovskites.

Shi et al. reported the first study^68^ on the DMA incorporation (*x* = 0.11) in MAPbI_3_. The authors found that mixing with DMA suppresses the tetragonal symmetry by stabilizing the cubic phase at room temperature, which provided a substantial boost in the carrier diffusion length and device stability. A similar work was reported by Shao et al.,^78^ where a series of compositions up to *x* = 0.25 were investigated and also linked to the enhanced photovoltaic performance and device stability. A stabilization of the cubic phase at room temperature for DMA concentrations higher than 6% was observed. The authors claimed that the cubic space group of the mixed compounds is noncentrosymmetric *P*4̅3*m*, which is different from that of pure MAPbI_3_ (centrosymmetric *Pm*3̅*m*). The absence of the centrosymmetry was not discussed or proved by the authors, making this assignment questionable. The same work also reported a rather coarse temperature dependence of the PXRD patterns of the MA_0.91_DMA_0.09_PbI_3_ sample showing that the cubic-tetragonal phase transition occurs at about 273 K. Surprisingly, the authors claimed the orthorhombic symmetry already below 233 K, which is a much higher transition temperature compared to pure MAPbI_3_ (see Figure 3).

Franssen et al.^208^ reported a solid-state NMR and PXRD study of the mixed MA_1–*x*_DMA_*x*_PbI_3_ (*x* ≤ 0.21) system. The authors obtained that the DMA solubility limit in MAPbI_3_ is 21%, above which the mixed phase was formed. In agreement with other works, stabilization of the cubic phase at room temperature was also obtained even for small levels of mixing. In addition, the authors performed ^14^N NMR relaxation experiments of the *x* = 0.21 sample and obtained similar reorientation times for both MA and DMA cations (close to 2 ps) at room temperature. This indicates that despite its bigger size, the DMA cation is still mobile in the mixed structure at room temperature.

**Figure 10:**
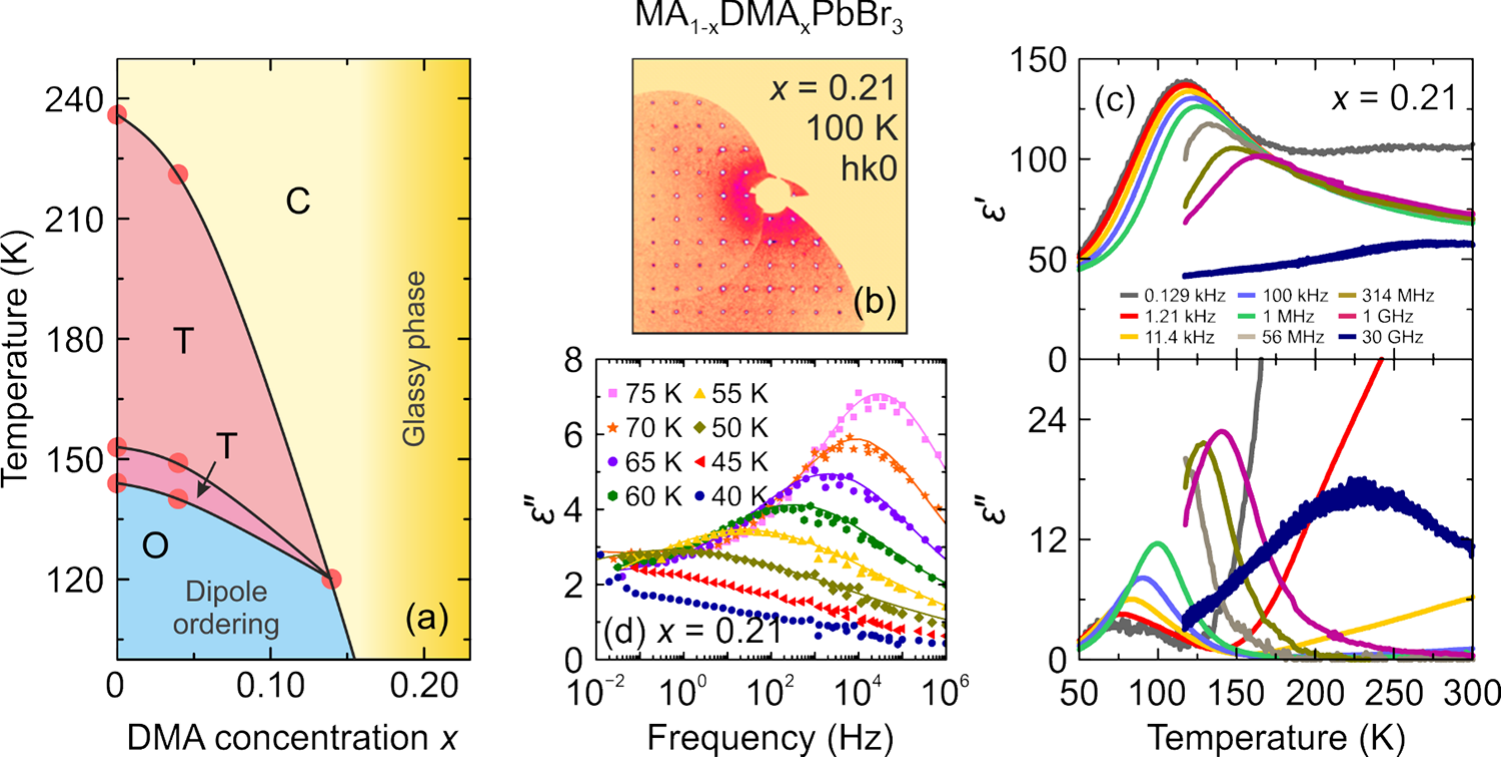
(a) Tentative temperature–composition phase diagram of the mixed MA_1–*x*_DMA_*x*_PbBr_3_ system. Symmetry notation: C - cubic, T - tetragonal, O - orthorhombic. (b) Reciprocal space reconstruction (*hk*0 layer) of the *x* = 0.21 single crystal sample measured at 100 K indicating cubic symmetry. (c) Temperature dependence of the broadband dielectric response of the *x* = 0.21 single crystal sample. (d) Low-frequency dependence of ε″ at selected temperatures of the same sample. Solid curves are the best fits to the Cole–Cole relaxation model. Adapted with permission from ref (164). Copyright 2020 Springer Nature.

The structural phase transitions and cation dynamics in the single crystals of mixed MA_1–*x*_DMA_*x*_PbBr_3_ bromide analogues were investigated by us using a suite of different experimental and theoretical techniques.^164^ The incorporated DMA fractions were determined to be 4, 14, and 21% by measuring the atomic C/N ratio. Crystals with higher mixing levels were not synthesized due to the finite DMA solubility limit in MAPbBr_3_ of about 30% reported by Anelli et al.^86^ The phase transition behavior in the mixed compounds was measured using temperature-dependent DSC, ultrasonic propagation, SCXRD, and broadband DS experiments. Upon introduction of a small amount of DMA, the temperatures of all three phase transitions decreased, indicating increased stability of the cubic phase as indicated in the phase diagram of this system (Figure 10a). Such a behavior is in agreement with the results obtained for the iodide analogues.^68,78,208^ For the *x* = 0.14 composition, no clear transition anomalies were observed in the DSC, ultrasonic, and DS experiments; however, the SCXRD data indicated a transition below 130 K to a phase having a similar symmetry to the orthorhombic phase observed for the *x* = 0 and 0.04 compositions. No symmetry lowering was observed for the highest mixing level (*x* = 0.21) as evident from the reciprocal space reconstruction (Figure 10b), suggesting a complete suppression of the phase transitions for this DMA concentration.

The same study^164^ reported the broadband DS experiments of the MA_1–*x*_DMA_*x*_PbBr_3_ single crystals in a vast frequency range covering more than 11 orders of magnitude in frequency (from mHz to GHz). For small mixing levels (*x* = 0.04), the temperature dependence of the dielectric response was essentially the same as for the MAPbBr_3_ compound with the tetragonal-orthorhombic phase transition anomaly being the dominant feature. However, a drastically different situation was observed for higher mixings (*x* = 0.14 and 0.21), where the transition anomaly was replaced by a very broad and highly frequency-dependent peak spanning from room temperature to 50 K at microwave and low frequencies, respectively (Figure 10c). The additional low-temperature experiments covering the mHz frequency range revealed very slow dynamics extending to even lower frequencies and temperatures (Figure 10d). These results demonstrated a gradual slowing down of the MA cation dynamics by approximately 12 orders of magnitude and implied formation of a glassy disordered phase (compare with Figure 2c). This behavior was also supported by the low-temperature heat capacity measurements, which revealed higher entropy states below 5 K only for the highly mixed compounds. In addition, the activation energy of the MA cation dynamics was determined from the frequency domain data, indicating close to 2-fold increase of *E*_a_ from 80 to 140 meV as the DMA content was increased. Note that a very similar behavior was also observed for the already discussed mixed MA_1–*x*_FA_*x*_PbBr_3_ system.^165^

**Figure 11:**
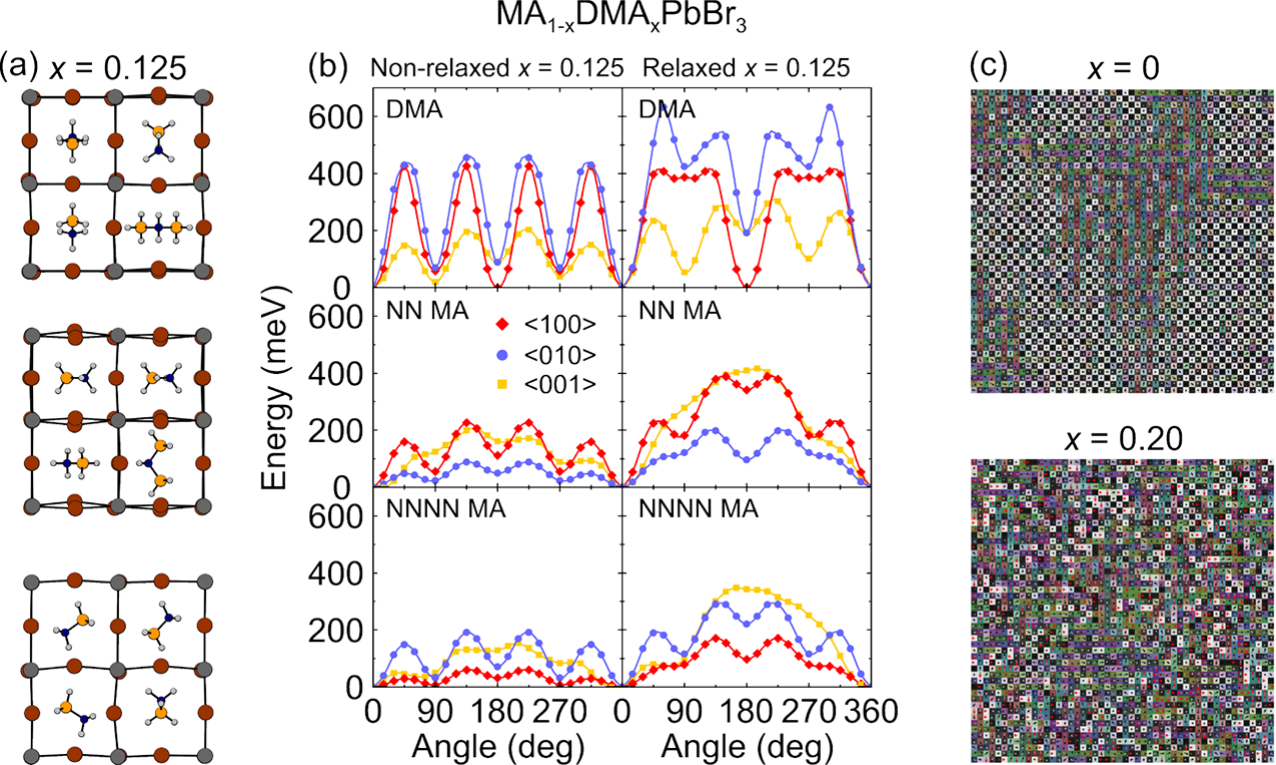
(a) Lowest energy structure of the *x* = 0.125 supercell obtained by DFT calculations, as seen from the <100> (top), <010> (middle), and <001> (bottom) directions. For clarity, only a 2 × 2 × 1 slab containing DMA is presented. (b) DFT calculations of the energy surfaces obtained by rotating DMA and neighboring MA cations around the <100>, < 010>, and <001> directions within the nonrelaxed and relaxed *x* = 0.125 systems. (c) Snapshots of the MC simulations for a two-dimensional slice of a three-dimensional periodic slab representing the *x* = 0 and *x* = 0.2 compositions. The orientations of the MA and DMA dipoles are depicted by gray and red arrowheads, respectively. Reprinted with permission from ref (164). Copyright 2020 Springer Nature.

In the same work, we also reported DFT and MC calculations of the mixed compositions supporting the experimental results.^164^ The DFT calculations revealed that the DMA cation is situated in the center of the lead–bromine cuboid cavity with two amine protons pointing to the halogens and the C–C bond directed along the <001> (or equivalent) direction (Figure 11a). The same calculations also provided rotation barriers around three orthogonal lattice directions of the DMA and two neighboring (nearest neighbor (NN) and next–next-nearest neighbor (NNNN)) MA cations (Figure 11b). A comparison was made between fully relaxed pseudotetragonal and nonrelaxed cubic (based on pure MAPbBr_3_, where a single MA was replaced by DMA) structures in order to clarify the effect of DMA incorporation on the neighboring MA cations. The rotation barrier of the DMA cation was found to be significantly higher than that of the MA cations in both structures, indicating that the DMA motion should be significantly hindered. Second, substantially higher rotation potentials of the MA cations were obtained in the relaxed structure demonstrating that the DMA cation perturbs their rotation via the lattice deformation. Third, the energy barrier was found to decrease with increasing distance to the DMA cation reflecting that the lattice deformation is a local effect. Based on these findings, a microscopic picture of the glassy phase formation was proposed, where a random distribution of DMA cations causes different local framework distortions resulting in a multiwell potential for MA cations. This results in cation frustration followed by the glassy phase formation and suppression of the structural phase transitions. Signatures of the orientational glass phase were also obtained using the MC simulations based on the dipole–dipole interaction model developed by the Walsh group.^244^ Here, the MA cation (dipole) was allowed to rotate, while the orientation of the DMA dipole was randomly fixed toward a <100> (or equivalent) lattice direction, which should be a valid approximation at low temperature. Despite a simplified nature of this mode, a long-range order emerged for low mixing levels, while no ordering was observed for *x* = 0.2 indicating that the MA dipoles experience severe competing interactions (Figure 11c).^164^

A subsequent study of the mixed MA_1–*x*_DMA_*x*_PbBr_3_ system was published by Ray et al.,^74^ where a substantially higher DMA incorporation fraction of 44% (supported by solution ^1^H NMR) was reported using solvent acidolysis crystallization. The temperature-dependent synchrotron radiation PXRD experiments were performed to study the phase symmetry of the *x* = 0.44 composition. A symmetry lowering from cubic to the tetragonal symmetry was observed at about 205 K, and no orthorhombic phase was recorded down to 80 K. This result is in contrast with the study discussed above,^164^ where the cubic phase was maintained at least down to 100 K for the *x* = 0.21 composition (Figure 10a). A likely explanation for this discrepancy is that, in contrast to high-resolution synchrotron PXRD, the conventional SCXRD was not sufficient to resolve small changes associated with this symmetry lowering. Interestingly, the authors claimed that the space group of this tetragonal phase is *P*4/*mbm*, while pure MAPbI_3_ shows the *I*4/*mcm* symmetry. This transformation was explained by the difference in H-bonding between the molecular cations and the inorganic framework. Note that the identical change of the tetragonal unit cell symmetry upon mixing was observed for the MA_1–*x*_Cs_*x*_PbBr_3_ system (Figure 9a).^163^

#### 4.2.4 **MA**_**1–*****x***_**EA**_***x***_**PbI**_**3**_

The ethylammonium cation has an effective radius of 274 pm (Table 1), making it a good candidate for incorporation in lead halide perovskites. Similarly to other big cations, pure EAPbI_3_ compound does not form a 3D perovskite and instead crystallizes into a 1D topology.^166,347−349^

Peng et al. reported the first structural study of the mixed MA_0.83_EA_0.17_PbI_3_ system,^350^ where the concentration of the incorporated EA cations was determined using the C/N analysis and solution NMR spectroscopy. Using PXRD and SCXRD methods, the authors observed cubic *Pm*3̅*m* symmetry at room temperature, indicating that EA cations also stabilize the desirable cubic phase.^122^ The temperature-dependent PXRD experiments revealed that the phase transition to the tetragonal phase occurs at about 243 K, which is almost 90 K lower compared to MAPbI_3_. Stabilization of the cubic symmetry at room temperature was also observed in two other studies^68,351^ reporting very similar levels of mixing (*x* = 0.15 and 0.14). Surprisingly, another study^352^ of MA_1–*x*_EA_*x*_PbI_3_ thin films reported a tetragonal symmetry at room temperature up to *x* = 0.3 EA fraction, above which the phase separation occurred. A DFT study by Liu et al.^84^ considered the whole range of mixed compositions (*x* = 0, 0.25, 0.5, 0.75, and 1) and found almost negligible changes of the Pb–I– Pb bond angles upon mixing with reasonable levels of EA cations (*x* ≤ 0.5).

**Figure 12:**
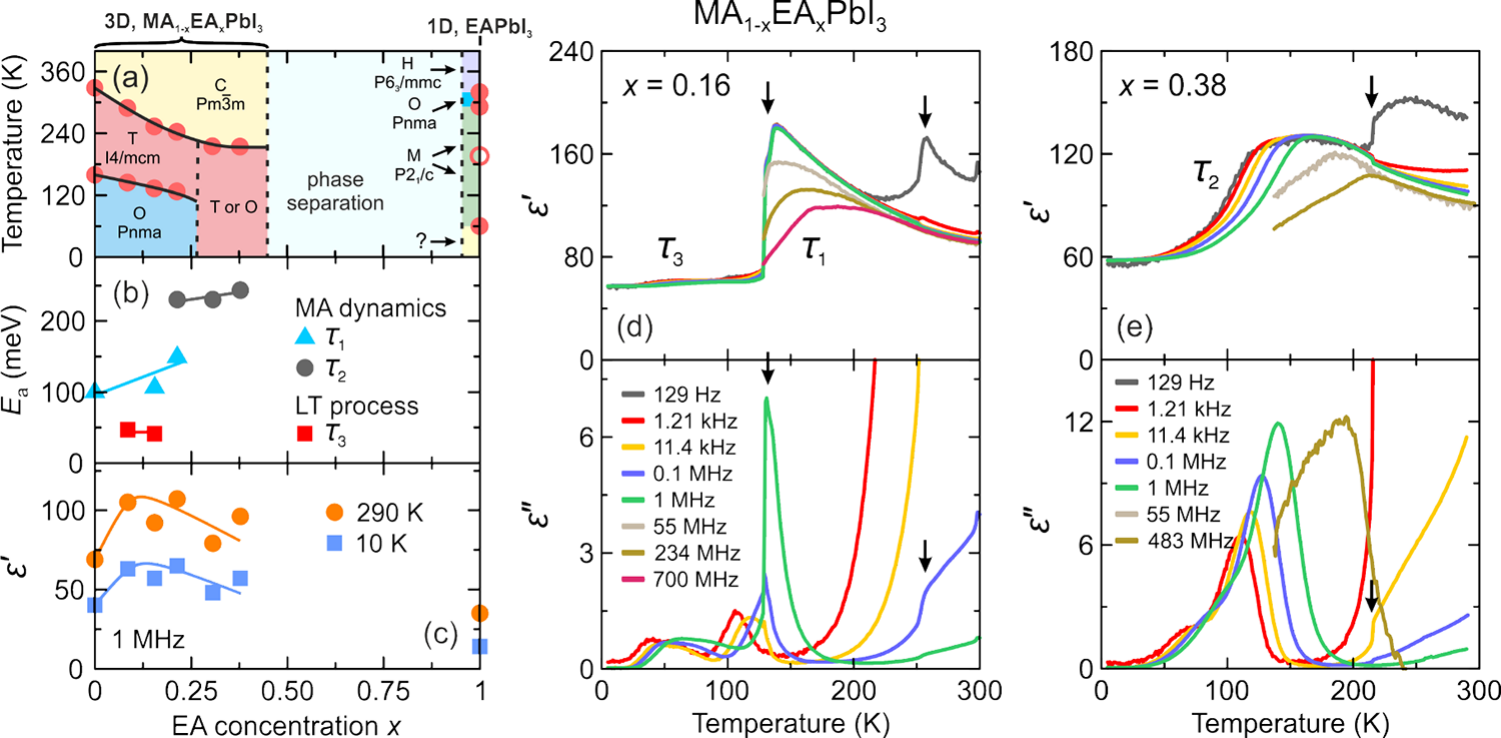
(a) Temperature–composition phase diagram of the mixed MA_1–*x*_EA_*x*_PbI_3_ system. Symmetry notation: C - cubic, T - tetragonal, O - orthorhombic, M - monoclinic, H - hexagonal. EA concentration dependence of the (b) activation energies and (c) ε′ (1 MHz) obtained at 290 and 10 K. (d) Temperature dependence of the complex dielectric permittivity of (d) MA_0.84_EA_0.16_PbI_3_ and (e) MA_0.62_EA_0.38_PbI_3_ single crystals presented at selected frequencies. Arrows indicate phase-transition anomalies, while dielectric relaxations are marked by τ_1_, τ_2_, and τ_3_. Reprinted with permission from ref (166). Copyright 2022 American Chemical Society.

A detailed multitechnique study of the mixing effects on the structural phase transitions, cations dynamics and PL of the MA_1–*x*_EA_*x*_PbI_3_ (*x* = 0, 0.09, 0.16, 0.21, 0.31, and 0.38) compounds was recently reported by us.^166^ The EA concentration was determined using solution ^1^H NMR spectroscopy, and the solubility limit of about 40% was found. A set of different experimental techniques (DSC, *C*_*p*_, ultrasonic, SCXRD, broadband DS, and Raman spectroscopy) was used to construct the temperature–composition phase diagram of this system (Figure 12a). For low and intermediate levels of mixing, the temperature of the cubic-tetragonal phase transition significantly decreased with increasing *x*, indicating stabilization of the cubic phase. For *x* = 0.16, this transition occurred at 253 K in a good agreement with the temperature of 243 K reported by Peng et al. for the *x* = 0.17 composition.^350^ The temperature of the tetragonal-orthorhombic phase transition also exhibited a decrease with increasing EA content, although the effect was slightly less pronounced (Figure 12a). The situation changed substantially for higher mixing levels (*x* > 0.3), where this transition became completely suppressed, and the low-temperature phase was described by a primitive tetragonal or pseudotetragonal (with very weak orthorhombic deformation) unit cell. However, due to the pseudomerohedral twinning, a reliable model of this structure was not obtained. The temperature-dependent Raman experiments of this phase revealed a substantial disorder related to the molecular cations even at low temperatures. The phase transitions of the pure 1D EAPbI_3_ compound were also investigated providing hexagonal-orthorhombic-monoclinic symmetry lowering (also summarized in Figure 12a). Another transition was also observed at an unusually low temperature of 60 K, but the symmetry below this point was not determined.

The broadband DS was employed in the same work^166^ to probe the dipolar dynamics of the mixed MA_1–*x*_EA_*x*_PbI_3_ single crystal compounds. For the intermediate values of *x*, the tetragonal-orthorhombic transition anomaly became broader (Figure 12d) and shifted to a lower temperature. In addition to the expected dipolar relaxation of MA cations in the tetragonal phase (indicated by τ_1_ in Figure 12d), an additional dipolar process τ_3_ of undetermined origin emerged below 100 K. For *x* > 0.3, the transition anomaly transformed into a broad dipolar relaxation of MA cations extending to low temperatures (indicated by τ_2_ in Figure 12e). The activation energies of both processes associated with the MA dynamics increased with increasing level of mixing (Figure 12b). Note that this effect was also observed in the MA_1–*x*_FA_*x*_PbBr_3_ and MA_1–*x*_DMA_*x*_PbBr_3_ systems suggesting universal behavior.^164,165^ Upon mixing, the dielectric permittivity of the MA_1–*x*_EA_*x*_PbI_3_ compounds also exhibited some tuning (Figure 12c), although the effect was rather small likely due to comparable electric dipole moments of both molecular cations (Table 1).

The observed broad dielectric response for the *x* > 0.3 compositions is similar to other highly mixed compounds (Figures 6, 8, and 10), and, as discussed above, resembles the glassy phase (compare with Figure 2c). However, a clear Vogel–Fulcher behavior of the mean relaxation time was not detected despite broadband measurements (Hz-GHz),^166^ indicating that freezing of electric dipoles may occur at very low temperatures. Note that the DFT calculations of the MA_0.875_EA_0.125_PbI_3_ composition revealed about a 20% variation of the rotational barrier for crystallographically different MA cations, indicating that the MA dynamics are affected by the local lattice strains introduced by bigger EA cations.

#### 4.2.5 **MA**_**1–*****x***_**GA**_***x***_**PbI**_**3**_

Guanidinium is another highly promising molecular cation used to significantly enhance the performance of the devices based on lead halide perovskites.^69,73,75,75,77,80^ The effective radius of this cation is 278 pm, which is practically the same as of DMA and EA (Table 1). For this reason, we may expect similar mixing effects on the structural and dynamic properties of lead halide perovskites.

Several studies reported contrasting solubility limits of these cations in MAPbI_3_. Jodlowski et al.^69^ reported a solubility limit of 25% in the perovskite film samples, and a similar value of 20% was claimed in another study.^353^ A significantly higher solubility limit of 40% (based on ^13^C MAS NMR measurements) was reported by Kubicki et al.^75^ for powder samples obtained by mechanosynthesis. Contrary to these studies, Gao et al.^80^ measured the solubility limit using NMR to be only 5% in single crystal samples. This result is also in sharp contrast to the solubility limits obtained for other similar size cations such as DMA and EA, prompting further studies to clarify this discrepancy.

**Figure 13:**
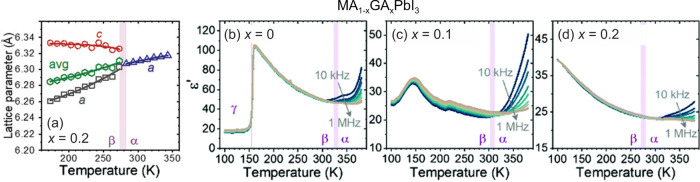
(a) Variation of the lattice parameters with temperature of the MA_0.8_GA_0.2_PbI_3_ system as determined from the XRD data. (b–d) Temperature dependence of the real part of the complex dielectric permittivity ε′ of the MA_1–*x*_GA_*x*_PbI_3_ pellet samples at frequencies between 10 kHz and 1 MHz. Reprinted with permission from ref (355). Copyright 2022 Royal Society of Chemistry.

Regarding structural properties, several studies reported preservation of the tetragonal symmetry at room temperature in the mixed MA_1–*x*_GA_*x*_PbI_3_ system.^69,73,75,80,353,354^ This observation is also in contrast to many other already discussed mixed-cation systems, where stabilization of the cubic phase was observed. Gao et al.^80^ observed almost negligible changes of the octahedral tilting and Pb–I–Pb bond lengths and angles for the mixing compositions of *x* = 0.05 and 0.1 showing that these systems remained tetragonal at room temperature. This discrepancy was also recognized and investigated in more detail in a recent study by Minussi et al.,^355^ where the phase transition and dielectric properties of the mixed MA_1–*x*_GA_*x*_PbI_3_ (*x* = 0, 0.1, 0.2) system were reported. The authors performed temperature-dependent XRD and DSC experiments, which indeed revealed stabilization of the cubic phase for the *x* = 0.2 compound at room temperature (Figure 13a). This removed the GA cation ambiguity and placed it in the same league with other molecular cations of similar size such as DMA, EA, and FA.

The same work also reported a temperature dependence of the real part of the complex dielectric permittivity ε′ of the MA_1–*x*_GA_*x*_PbI_3_ pellet samples in a narrow frequency range (10 kHz - 1 MHz)^355^ (Figure 13b–d). The obtained results for pure MAPbI_3_ compound show an expected anomalous decrease of the permittivity at the tetragonal-orthorhombic phase transition point in agreement with other studies.^183,340,341^ For the *x* = 0.1 composition, this anomaly became significantly broader, and its maximum shifted to a slightly lower temperature, suggesting that the stability of the tetragonal phase is also increased. The peak of the anomaly was not detected for the *x* = 0.2 compound likely due to the limited temperature range of the reported experiment. In addition, the authors observed a significant decrease of the dielectric permittivity upon mixing, which can be explained by a negligible electric dipole moment of GA (Table 1).

The dynamics of the molecular cations in the MA_1–*x*_GA_*x*_PbI_3_ solid solutions was investigated by the Emsley group using MAS NMR spectroscopy. Their first study^75^ reported the reorientation rates of both molecular cations for the *x* = 0.25 composition by fitting the corresponding ^14^N NMR spectra. Such an analysis provided 113 ps and less than 18 ps reorientations rates of MA and GA cations at room temperature, respectively. The observed almost 1 order of magnitude faster GA motion was also correlated to a spectacular increase of the charge carrier lifetimes in this mixed compound. However, a recent study from the same group^204^ reevaluated these findings using more reliable quadrupolar relaxation measurements, which provided much shorter reorientation times of both cations for the same mixed composition (*x* = 0.25). The MA dynamics perpendicular to the *C*_3_ molecular axis were found to occur on the 1.3 ps time scale with the activation energy of 168 meV at room temperature. A substantially faster rate of 0.3 ps was measured around the *C*_3_ axis with a negligible energy barrier. Note that MA cations in pure MAPbI_3_ showed very similar reorientation rates, indicating a weak mixing effect on the MA cation motion at room temperature. The dynamics of the GA cation was also characterized by two distinct principal axes owing to its *C*_3_ symmetry. Here, the motion perpendicular to this axis was obtained to be much faster (1.3 ps, *E*_a_ = 121 meV) compared to the parallel reorientation (14 ps) in line with different moments of inertia.

#### 4.2.6 **MA**_**1–*****x*****–*****y***_**GA**_***x***_**FA**_***y***_**PbI**_**3**_

Recently, the Araújo group reported several studies of triple-cation MA_1–*x*–*y*_GA_*x*_FA_*y*_PbI_3_ solid solutions obtained by mechanosynthesis. In the first work,^356^ the authors used PXRD, DSC, and IR spectroscopy to study the structural phase transitions and cation dynamics for the case, where the GA and FA content was equal (i.e., *x* = *y*) and did not exceed 30% of the total MA substitution. Upon increase of *x* and *y*, it was found that the cubic-tetragonal phase transition temperature is substantially lowered to about 270 K at the maximum mixing level (*x* = *y* = 0.15), indicating increased stability of the cubic phase. The IR spectroscopy measurements revealed a gradual shift of the C–N stretching frequency for all organic cations with mixing, which was assigned to the increase of the bond strength. The intensity of the IR bands also exhibited dependence on the mixing level especially when crossing the cubic-tetragonal phase transition. This behavior was related to the degree of cation freedom within differently deformed inorganic framework in the cubic and tetragonal phases.

**Figure 14:**
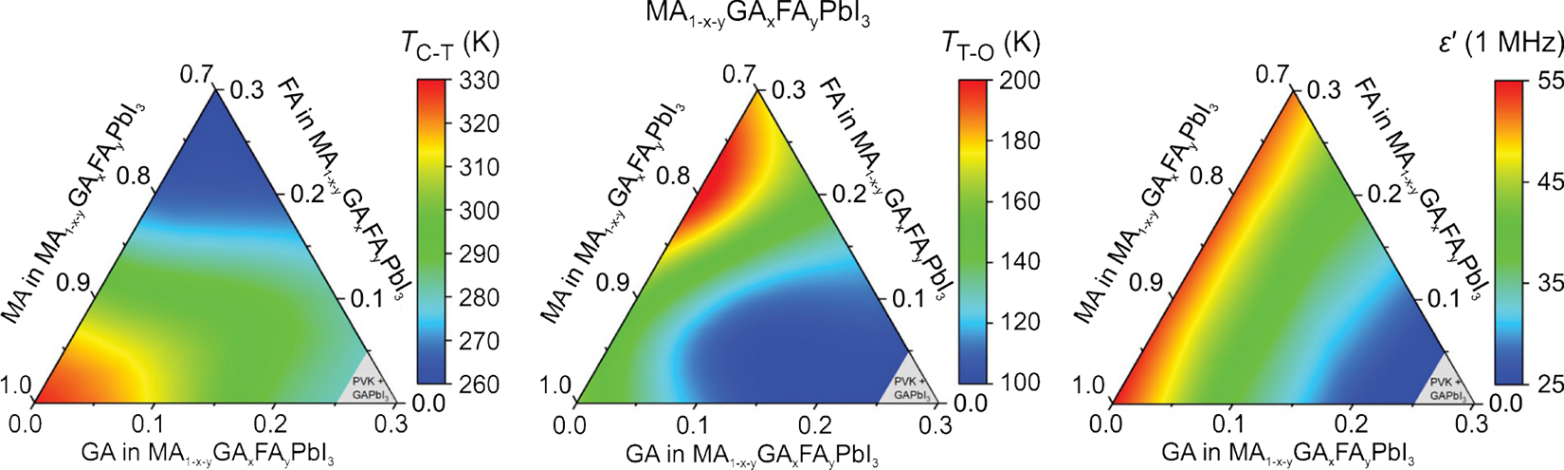
Phase diagrams for the (left) cubic-tetragonal and (middle) tetragonal-orthorhombic phase transitions of the MA_1–*x*–*y*_GA_*x*_FA_*y*_PbI_3_ system. (Right) Composition map for of the real part of the complex dielectric permittivity ε′ obtained at room temperature and 1 MHz probing frequency. Reprinted with permission from ref (357). Copyright 2023 Wiley-VCH.

Subsequent XRD, DSC, and DS studies from the same group^357,358^ reported a thorough investigation of the MA_1–*x*–*y*_GA_*x*_FA_*y*_PbI_3_ solid solutions for 0 ≤ *x* ≤ 0.3 and 0 ≤ *y* ≤ 0.3 with *x* + *y* ≤ 0.3. The reported composition dependence of the cubic-tetragonal phase transition temperature is presented in Figure 14a, revealing that FA cations are more effective in stabilizing the cubic phase compared to GA.^357^ Interestingly, the opposite behavior is observed for the tetragonal-orthorhombic phase transition, where the GA-rich compositions have significantly lower transition temperatures (Figure 14b). Both observations clearly indicate that GA cations are more efficient in stabilization of the tetragonal phase compared to FA cations.

In addition to phase transition studies, the same study reported the DS experiments of the MA_1–*x*–*y*_GA_*x*_FA_*y*_PbI_3_ pellet samples.^357^ The measured values of ε′ obtained at room temperature and 1 MHz probing frequency are summarized in Figure 14c, indicating that GA incorporation results in a significantly more pronounced drop of the dielectric permittivity compared to FA cations. This result is in agreement with the aforementioned DS studies of the MA_1–*x*_FA_*x*_PbBr_3_^165^ and MA_1–*x*_GA_*x*_PbI_3_^355^ solid solutions. Such a contrasting FA and GA cation effect on the dielectric response might be related to the absence of the electric dipole moment of GA, although the dipole moment of FA is also almost negligible compared to MA (see Table 1). As also pointed out by the authors, this behavior may also indicate that both cations exert a different hindering effect on the neighboring MA cations. However, this interpretation seems to contradict the recent NMR study by Mishra et al.,^204^ where the effect of the incorporated GA and FA cations on the MA cation motion was found to be small in MA_1–*x*_FA_*x*_PbI_3_ and MA_1–*x*_GA_*x*_PbI_3_ compounds, suggesting a more intricate mechanism. We also note that the reported DS experiments were performed on the pressed pellet samples, which always introduces a degree of uncertainty in the determination of the dielectric permittivity.

### 4.3 FA-Based Compounds

The current best performing hybrid perovskites for photovoltaic applications are based on the mixed compositions with α-FAPbI_3_.^8,9,11^ Despite this significance, the mixing effects on the structural phase transitions and dynamics in these compounds are substantially less investigated compared to the MA-based perovskites. In this section, we review the available studies, which mostly concentrate on mixing with Cs^+^ and GA cations, as mixing with MA is covered above. Note that many works report stabilization of the photoactive black α-phase of FAPbI_3_ upon mixing (e.g., refs (11, 72, 76, 110, 359).). However, the majority of these studies fall out of scope of this review, as they concentrate on utilization of the stabilized α-FAPbI_3_ phase for device fabrication and provide no or very little additional information about the structural and dynamic effects.

#### 4.3.1 **FA**_**1–*****x***_**Cs**_***x***_**PbX**_**3**_**(X = I, Br)**

**Figure 15:**
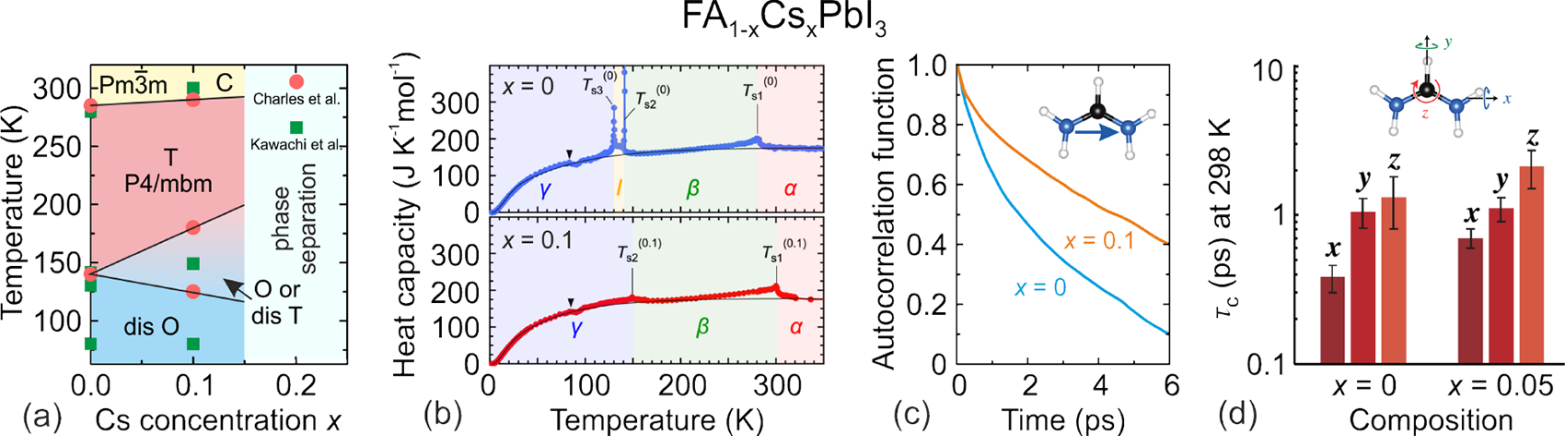
(a) Tentative temperature–composition phase diagram of the mixed FA_1–*x*_Cs_*x*_PbI_3_ system. Symmetry notation: C - cubic, T - tetragonal, O - orthorhombic, disT - disordered tetragonal, disO - disordered orthorhombic. Adapted with permission from ref (171). Copyright 2020 American Chemical Society. (b) Temperature dependence of the heat capacity of the *x* = 0 and *x* = 0.1 compounds. Reprinted with permission from ref (186). Copyright 2019 American Chemical Society. (c) Vector autocorrelation function of FA cation in the *x* = 0 and *x* = 0.1 compositions showing the probability of the cation remaining in its initial orientation over time. Molecular vector is indicated in the inset. Adapted with permission from ref (226). Copyright 2017 American Chemical Society. (d) Anisotropic rotational correlation time (298 K) of FA cations in the *x* = 0 and *x* = 0.05 compounds determined using NMR. Reprinted with permission from ref (204). Copyright 2023 American Chemical Society.

Charles et al.^171^ used a suite of diffraction techniques (neutron powder diffraction, SCXRD, PXRD) to thoroughly probe the phase transition behavior in the mixed FA_1–*x*_Cs_*x*_PbI_3_ system. A solubility limit of about 15% was determined using PXRD in agreement with the magnetic resonance studies,^207,360^ while a substantially higher limit close to 30% was reported for thin films.^95^ To avoid phase separation into black and nonperovskite yellow components observed for higher mixing levels, the authors selected *x* = 0.1 concentration for a more detailed investigation. The room-temperature diffraction measurements revealed that the time averaged structure of this compound has a cubic *Pm*3̅*m* symmetry in agreement with pure FAPbI_3_ perovskite (Figure 3). It is also noted that the local structure may contain pseudotetragonal domains due to tilted octahedra switching rapidly between preferred directions. The determined Pb–I–Pb angle in the mixed composition was found to be 162.8°, which is almost identical to the nonmixed FAPbI_3_ compound (163.7°).^222^ Interestingly, upon introduction of Cs^+^, the phase transition temperature to the tetragonal *P*4/*mbm* phase slightly increased (290 K), which likely occurs due to the significantly higher phase transition temperatures of CsPbI_3_. Below 180 K, the diffraction data were indexed to either crystallographic twins in the tetragonal space group *P*4/*mbm* or orthorhombic *Pnma*. A convergence to the orthorhombic *Pnma* symmetry was obtained below 125 K with additional weak reflections attributed to glass-like disorder, which was also proposed for pure FAPbI_3_.^209^ The transition temperature to this phase was lowered upon mixing. All these results are summarized in a crude phase diagram presented in Figure 15a. Note that signatures of the glassy phase formation at low temperature in the FA_1–*x*_Cs_*x*_PbI_3_ system were also observed in a recent MC study.^228^

Kawachi et al.^186^ reported heat capacity measurements of the high quality FA_1–*x*_Cs_*x*_PbI_3_ (*x* = 0 and 0.1) single crystals. For pure FAPbI_3_ compound, a second-order transition from the cubic to tetragonal symmetry was observed at 280 K (Figure 15b) in agreement with other works (Figure 3). Interestingly, two sequential first-order transitions at 141 and 130 K were observed, as the system evolved to the low-temperature phase. The origin of this intermediate phase was not investigated. An additional weak anomaly was detected at about 80 K. The temperature dependence of the heat capacity of the mixed (*x* = 0.1) crystal revealed that the cubic-tetragonal phase transition shifted to higher temperatures (300 K) (Figure 15b). In addition, mixing resulted in a complete suppression of the intermediate phase, as a single phase transition was detected at 149 K, while the low-temperature anomaly at about 80 K was unaltered. Here we note that mixing also suppressed the intermediate phase of MAPbBr_3_.^165^ The measured transition temperature of 149 K is not in line with the results reported by Charles et al.^171^ for the same mixed composition, where transitions were observed at 125 and 180 K (see Figure 15a for comparison). Further studies are needed to clarify this discrepancy.

Ghosh et al.^226^ used *ab initio* MD simulations to study the microscopic dynamics in the mixed FA_0.9_Cs_0.1_PbI_3_ and FA_0.9_Rb_0.1_PbI_3_ perovskites. The authors reported that the introduction of smaller cations such as Cs^+^ and Rb^+^ (Table 1) results in a greater tilt angle of the octahedra compared to pure FAPbI_3_. In addition, this tilting is locked, and thus the octahedra undergo much more restricted rocking dynamics. This also affects the neighboring molecular cations, which form stronger intermolecular N–H···I H-bonds. As a result, it was observed that the tumbling time scale of the FA cations slowed down more than twice from 2 ps to almost 5 ps (Figure 15c) upon introduction of 10% of Cs. For comparison, the anisotropic rotational correlation time of FA cations in the *x* = 0 and 0.05 compounds was recently measured using quadrupolar relaxation NMR experiments.^204^ Slightly slower dynamics were indeed observed in the mixed system, especially around the molecular *x*-axis (see Figure 15d), although the uncertainties of the determined values were rather high. Note that a later study found that, in contrast to Cs^+^, Rb^+^ ions have no capacity to be incorporated in FAPbI_3_.^207^

Mundt et al.^361^ studied FA_0.83_Cs_0.17_PbI_3_ thin films to demonstrate that nanoscale compositional heterogeneity can serve as initiation sites for more macroscale irreversible phase segregation. The degree of nanoscale heterogeneity is kinetically controlled and the well-mixed system can be reached when the sample is annealed either long enough or at sufficiently high temperature. In particular, the authors showed that the well-mixed sample, which can be achieved by annealing at 140 °C for 30 min, exhibits a sharp cubic-tetragonal phase transition at 50 °C, while the poorly mixed sample, prepared by annealing at 140 °C for 15 min, showed a smeared out phase transition, reaching the cubic phase close to 130 °C. The smeared phase transition was attributed to a higher level of nanoscale heterogeneity, since various local compositions exhibited phase transitions at various temperatures. This paper emphasized the importance of kinetics on formation of perovskite phases and consequently on the properties of the synthesized thin films.

**Figure 16:**
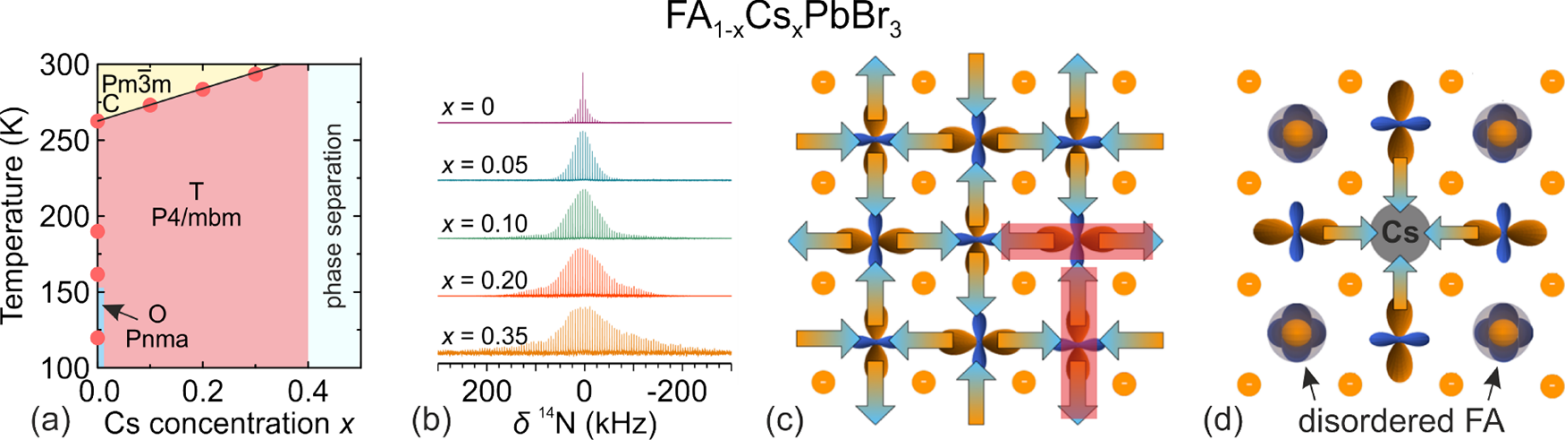
(a) Temperature–composition phase diagram of the mixed FA_1–*x*_Cs_*x*_PbBr_3_ system. Symmetry notation: C - cubic, T - tetragonal, O - orthorhombic. (b) Room-temperature ^14^N MAS NMR spectra of the FA_1–*x*_Cs_*x*_PbBr_3_ compounds. (c) Schematic cartoon illustrating the FA cation arrangement in a pure FAPbBr_3_ system. Such a “T”-type (red) long-range quadrupolar order is only possible in a two-dimensional plane. (d) Incorporated Cs^+^ ion disrupts the “T”-type arrangement, as neighboring FA cations point to the positive charge of Cs^+^ causing disordering of the next-nearest neighbor quadrupoles (black arrows). Reprinted (adapted) with permission from ref (172). Copyright 2020 American Chemical Society.

The mixed FA_1–*x*_Cs_*x*_PbBr_3_ bromide analogues were thoroughly investigated by Mozur et al.^172^ using XRD, neutron scattering, and NMR techniques. The solubility limit of Cs in FAPbBr_3_ was found to be about 40%, beyond which a phase separation occurred. Note that a very similar limit was also observed for the related MA_1–*x*_Cs_*x*_PbBr_3_ and FA_1–*x*_Cs_*x*_PbI_3_ systems.^110,163^ The authors used temperature-dependent high-resolution synchrotron XRD to map the phase diagram of FA_1–*x*_Cs_*x*_PbBr_3_ (Figure 16a). For all studied concentrations, a cubic-tetragonal phase transition was detected with its temperature shifting to higher values upon mixing. This indicates reduced stability of the cubic phase in agreement with the phase diagram of the FA_1–*x*_Cs_*x*_PbI_3_ system (Figure 15a). More importantly, a complete suppression of all other (tetragonal-orthorhombic and three isosymmetric^270^) phase transitions was observed even for the lowest mixing level (*x* = 0.05). However, as stated by the authors, the orthorhombic phase of pure FAPbBr_3_ already exhibits features of low intensity, which may be even more difficult to resolve in the mixed compositions. Note that a complete disappearance of the isosymmetric transformations of FAPbBr_3_ was also observed upon incorporation of a small amount of MA cations, indicating that these transitions are very sensitive to small perturbations.^165^

In the same study, the mechanism behind the suppression of the transitions was investigated in more detail.^172^ The room-temperature ^14^N NMR spectra of different compositions indicated increased disorder, i.e., the presence of a larger distribution of local FA environments upon mixing (Figure 16b). The same behavior was also observed for the inorganic framework using the ^79^Br nuclear quadrupole resonance spectroscopy. However, inelastic neutron scattering data revealed that mixing has only minimal influence on the internal vibrational modes of the FA cations. The ^1^H NMR relaxation time measurements supported this result by showing that the cation reorientation rates and activation energies are also relatively weakly affected by mixing. Based on these observations, the authors concluded that the incorporated Cs^+^ disrupts the FA cation ordering on the longer range, while their local degrees of freedom are retained. In the previous work,^270^ the same group proposed that the crystallographically unresolvable isosymmetric phase transitions of FAPbBr_3_ are caused by the long-range dynamics of the FA cations. This behavior is enabled by the electrostatic quadrupolar interactions between the cations (quadrupole moment |*Q*_11_| = 18.3 DÅ), which highly prefer the “T”-type (perpendicular) arrangement (Figure 16c). However, such long-range dynamics do not lead to a long-range order in the system, as it is not possible to form an ordered “T”-type structure in three dimensions, meaning that FA cations are already geometrically frustrated in pure FAPbBr_3_. Incorporation of Cs^+^ ions causes local alignment of the neighboring quadrupole moments disrupting the local “T”-type ordering (Figure 16d) and interrupting the cooperative dynamics associated with these transitions.

#### 4.3.2 **FA**_**1–*****x***_**GA**_***x***_**PbI**_**3**_

Mixing of GA in the structure of FAPbI_3_ was briefly studied by Kubicki et al.^75^ using room-temperature ^13^C and ^14^N MAS NMR spectroscopy of FA cations. The authors showed that incorporation of GA (*x* = 0.25) causes a significant broadening of the FA spectral envelope compared to pure α-FAPbI_3_. This indicates decreased reorientation dynamics of FA cations upon mixing with GA cations. Interestingly, it was also observed that the mixed FA_1–*x*_GA_*x*_PbI_3_ compounds are thermodynamically unstable and become yellow within hours after annealing, meaning that GA cations do not fully stabilize the desirable black phase.

### 4.4 Summary

Several general observations can be made regarding the A-site mixing effects on the structural and dynamic properties of MAPbX_3_ and FAPbX_3_ systems. As expected from the tolerance factor arguments, the size of the guest cation is related to the solubility limit, which ranges from 100% for compatible cations (MA/FA systems) to 20–40% for substantially bigger or smaller guests (DMA, EA, GA, Cs^+^). The mismatch in the cation size also greatly affects the structural and dynamic properties of the mixed compounds.

The A-site mixing with molecular cations is an effective recipe to suppress the structural phase transitions and thus symmetrize the lattice by stabilizing the desirable cubic phases.^122^ This does not hold for mixing with smaller Cs^+^ ions, which tend to stabilize the tetragonal phase likely inherited from pure CsPbX_3_ perovskites. The extent of transition suppression also depends on the cation size - mixing of relatively well compatible MA and FA cations results in a weak suppression, while introduction of big cations (low solubility limit) can cause a complete disappearance of the transitions. This behavior originates from a significant lattice deformation introduced by more bulky molecular cations, which disrupt the H-bonding and octahedral tilt patterns and thus prevent the cooperative long-range ordering.

**Figure 17:**
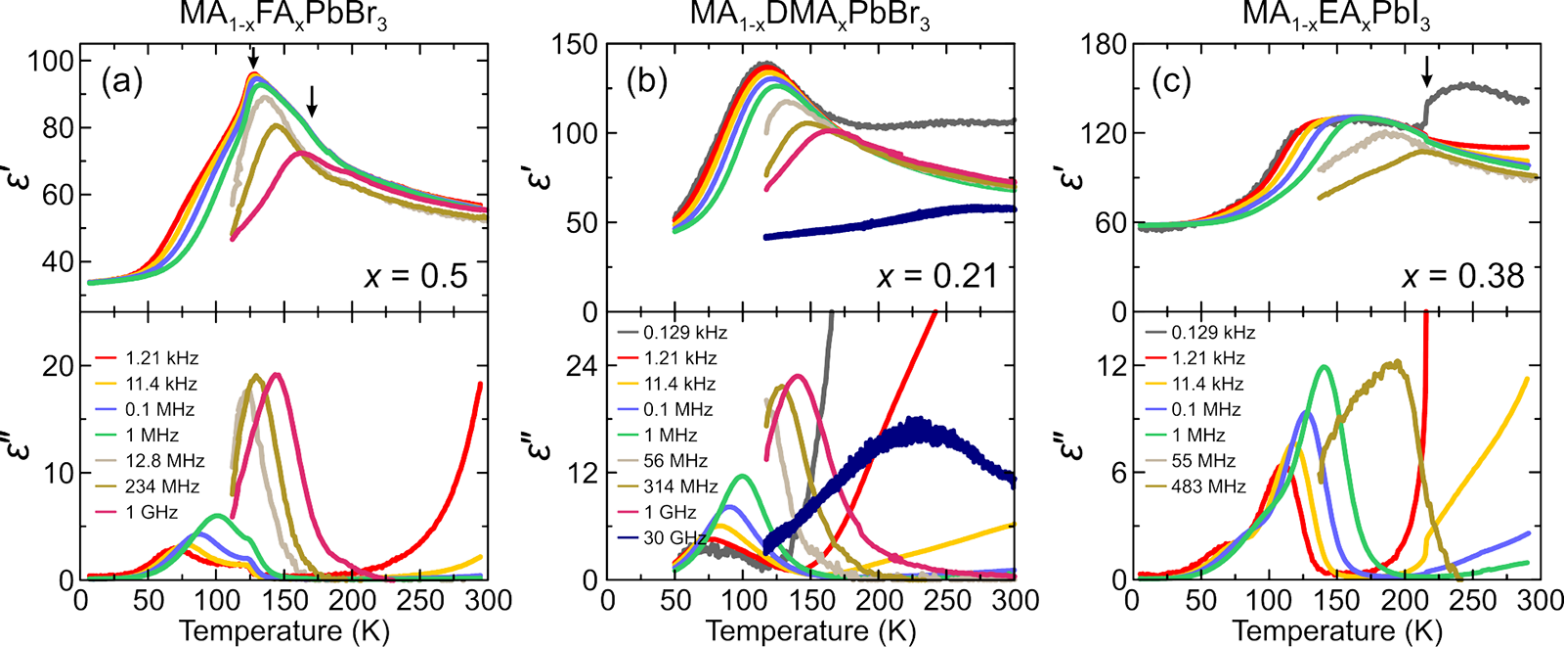
Comparison of the broadband DS of the highly mixed (a) MA_0.5_FA_0.5_PbBr_3_, (b) MA_0.79_DMA_0.21_PbBr_3_, and (c) MA_0.62_EA_0.38_PbI_3_ systems. (a) Reprinted (adapted) with permission from ref (165). Copyright 2021 American Chemical Society. (b) Reprinted (adapted) with permission from ref (164). Copyright 2020 Springer Nature. (c) Reprinted (adapted) with permission from ref (166). Copyright 2022 American Chemical Society.

Similarly to classical inorganic systems, the highly mixed lead halide perovskites also show very broad dielectric responses both in temperature and frequency domains. A comparison of different mixed systems (MA_0.5_FA_0.5_PbBr_3_, MA_0.79_DMA_0.21_PbBr_3_, and MA_0.62_EA_0.38_PbI_3_) is presented in Figure 17 revealing a broad dipolar dispersion of MA cations ranging from almost room temperature at the GHz band to very low temperatures at mHz frequencies (see also Figure 10d). Note that this dispersion is weakly affected by the incompletely suppressed structural phase transitions (e.g., in MA_0.5_FA_0.5_PbBr_3_). The main features of the observed dielectric response resemble the dipolar (orientational) glass (Figure 2c). A likely origin of this phase is frustrated interactions between the molecular cations, which are mediated by the lattice deformations introduced by mixing. It should also be noted that dipolar glass should exhibit freezing dynamics following the Vogel–Fulcher law, but no such behavior was detected for the mixed lead halide perovskites. A possible explanation for this discrepancy is that freezing actually happens, but at low temperatures, where the dipolar relaxation is already shifted to very low frequencies (sub mHz) making the DS experiments infeasible during a reasonable amount of time.

**Figure 18:**
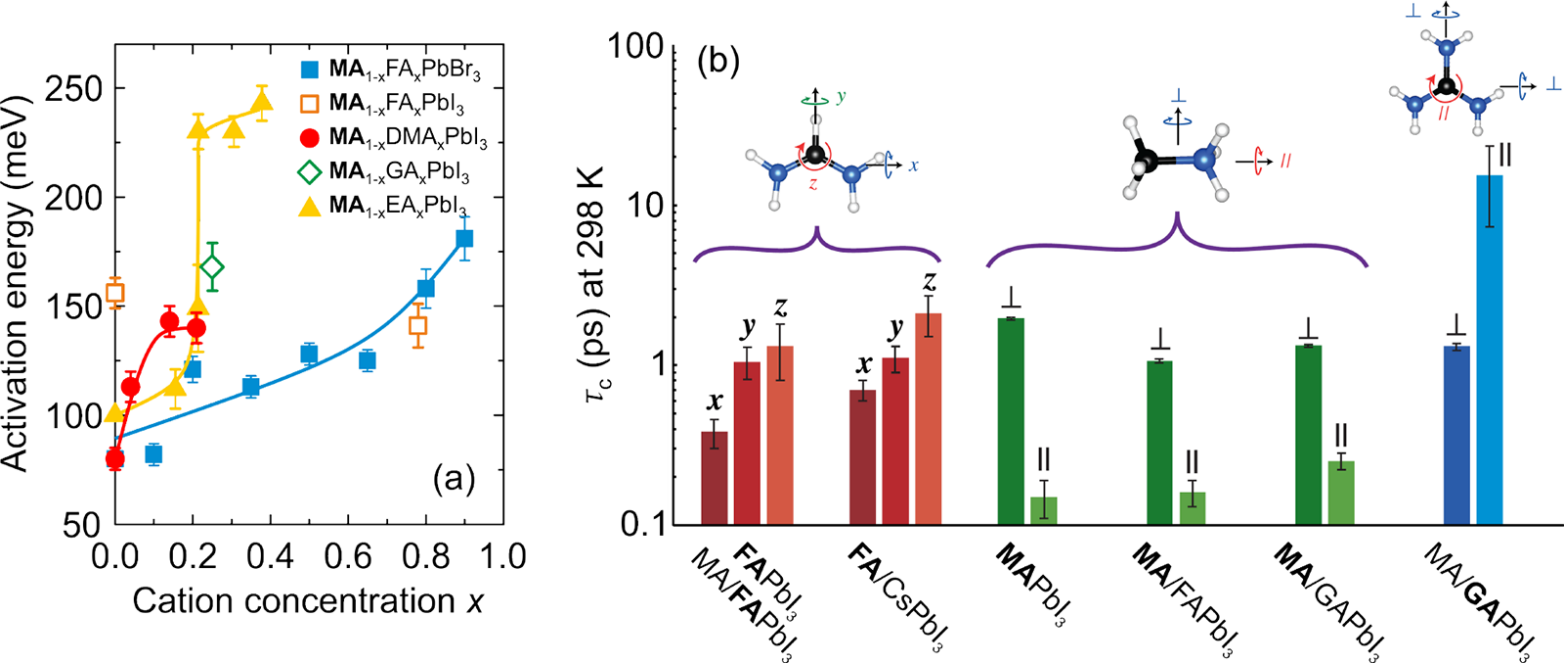
(a) Activation energy of MA cation motion vs guest cation concentration for different MA-based mixed systems determined using the dielectric (solid) and NMR (open points) spectroscopies. Solid curves are a guide for the eye. Data taken from refs (164−166, 204). (b) Rotational correlation times at room temperature of FA, MA, and GA molecular cations in different mixed perovskites obtained by NMR. Reprinted with permission from ref (204). Copyright 2023 American Chemical Society.

The activation energy of the MA motion in different mixed systems determined by the broadband DS is summarized in Figure 18a.^164−166^ An increase of *E*_a_ with increasing *x* is evident, indicating that guest cations raise the rotational barrier for MA cations. In contrast to DS, the activation energy of the MA reorientation at room temperature determined by NMR shows a negligible change upon mixing with FA and GA (Figure 18a), which is likely related to different temperature ranges probed by both techniques.

Here, we also show a summary of the room-temperature rotational correlation times of MA, FA, and GA cations in different mixed compounds determined in a recent NMR study (Figure 18b).^204^ Interestingly, all cations exhibit dynamics on a similar few ps time scale. For example, MA and FA reorientation times seem to be only weakly affected by mixing. Note that a very similar time scale in the range of few ps can be obtained by extrapolating the MA relaxation times obtained from the broadband DS (Figure 17). This shows that both techniques essentially probe the same type of dynamics.

## 5 X-Site Mixing in 3D Perovskites

Here, we survey how halide mixing at the X-site affects the structural, phase transition, and dynamic properties of the 3D lead halide perovskites. The majority of works, which fall within the scope of this review, describe the MA- and Cs-based compounds with an exception of MHy-based compositions.

### 5.1 MA-Based Compounds

#### 5.1.1 **MAPb(I**_**1–***x*_**Br**_***x***_**)**_**3**_

Noh et al. claimed a complete solubility of I^–^ and Br^–^ anions (effective radii 220 pm vs 196 pm^362^) at the X-site in the mixed MAPb(I_1–*x*_Br_*x*_)_3_ system.^363^ The authors also observed that the room-temperature crystal symmetry changes from tetragonal to cubic for bromine concentration *x* ≥ 0.2. A solubility limit was also not considered in more recent works, where the *x* = 1/3 and 2/3 compositions were investigated.^364,365^ However, as demonstrated by the Walsh group using DFT and free energy calculations, the MAPb(I_1–*x*_Br_*x*_)_3_ solid solution should exhibit a very wide miscibility gap (0.19 ≤ *x* ≤ 0.68) at room temperature, where the phase separation occurs.^366^

The latter result was also confirmed experimentally by Lehmann et al.^367^ using synchrotron PXRD, where an even wider miscibility gap of 0.29 ≤ *x* ≤ 0.92 was determined. In the iodine rich phase, a partial solubility of bromine was observed as revealed by a continuous shift of lattice parameters. In contrast, iodine formed cluster-like regions in the bromine-rich phase showing a very poor solubility.

**Figure 19:**
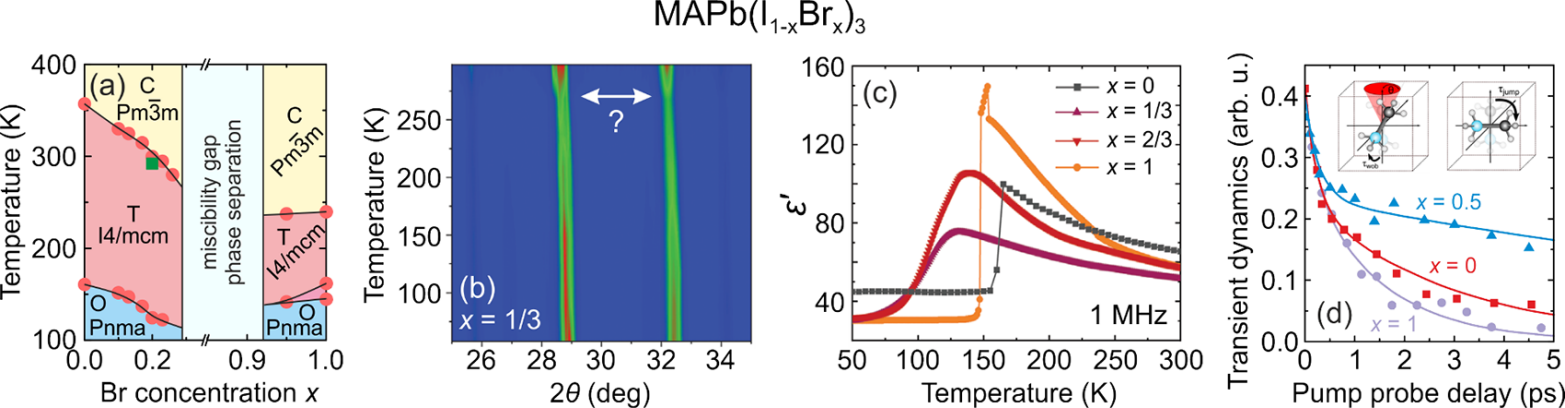
(a) Temperature–composition phase diagram of the MAPb(I_1–*x*_Br_*x*_)_3_ system. Symmetry notation: C - cubic, T - tetragonal, O - orthorhombic. Reprinted (adapted) with permission from ref (367). Copyright 2019 The Royal Society of Chemistry. Green square data point taken from refs (363, 368). (b) PXRD patterns of MAPbI_2_Br at different temperatures. (c) Temperature dependence of the real part of the dielectric permittivity of the *x* = 0, 1/3, 2/3, and 1 compounds measured 1 MHz frequency. Reprinted (adapted) with permission from ref (365). Copyright 2022 Wiley-VCH. (d) Transient anisotropy dynamics of MA cations in the *x* = 0, 0.5, and 1 compounds. Solid curves show the fits using the wobbling-in-a-cone relaxation model (inset). Adapted with permission from ref (369). Copyright 2017 American Chemical Society.

Based on the temperature-dependent PXRD experiments, the authors also mapped the temperature–composition phase diagram of the MAPb(I_1–*x*_Br_*x*_)_3_ system (Figure 19a).^367^ A clear decrease of both phase transition temperatures was observed upon mixing up to the miscibility gap in the iodine-rich region, indicating stabilization of the cubic phase. This behavior is analogous to mixing of molecular cations at the A-site. On the other end of the phase diagram, the cubic-tetragonal phase transition was found to be barely affected by mixing. However, the intermediate tetragonal phase of MAPbBr_3_ was suppressed indicating that, despite low solubility and tendency to cluster, iodine still perturbs the long-range order in the system.

Interestingly, Tang et al. found that the miscibility gap in the MAPb(I_1–*x*_Br_*x*_)_3_ system could be avoided using the mechanochemical synthesis instead of a typical solvent synthesis.^368^ In such a way, the authors synthesized the whole series of compositions and observed no signatures of phase separation. In addition, it was found that the system changes room-temperature symmetry from tetragonal to cubic at *x* ≈ 0.2 in agreement with other works.^363,367^ For comparison, the authors also prepared MAPb(I_1–*x*_Br_*x*_)_3_ films using the wet chemical route and observed a wide miscibility gap (0.1 < *x* < 0.8) in line with other studies.^366,367^ No phase separation was also observed in a recent NMR and MD work by Fykouras et al.,^205^ where mixed compositions with *x* = 1/3, 1/2, and 2/3 were synthesized by mechanochemical synthesis. In this study, the authors also demonstrated that iodine and bromine ions are randomly dispersed and that reorientation of the MA cations within the inorganic cage highly depends on a local halide distribution. Note that Askar et al. also found no miscibility gap and a homogeneous halide distribution in the related mixed FAPb(I_1–*x*_Br_*x*_)_3_ and FAPb(Br_1–*x*_Cl_*x*_)_3_ systems using ^207^Pb NMR.^370^

A recent study by Shahrokhi et al.^365^ reported a more detailed multitechnique investigation of the phase transitions in MAPbI_2_Br (*x* = 1/3) and MAPbIBr_2_ (*x* = 2/3) single crystal compounds obtained using solvent synthesis. As discussed above, while the former compound could be considered to be on the edge of the miscibility gap, the latter composition should result in phase separation and iodide clustering. However, this issue was not addressed, as full solubility was assumed. The authors claimed no anomalies in the DSC and PXRD data for both compounds, indicating a complete suppression of the phase transitions and stabilization of the cubic phase. This result is also not in line with the phase diagram provided by Lehmann et al.,^367^ where transitions at about 270 and 115 K can be expected at the lower edge of the miscibility gap *x* ≈ 0.3 (Figure 19a). Indeed, a closer look at the PXRD data reported by Shahrokhi et al.^365^ seems to show some change of the diffraction patterns at 275 K (Figure 19b), suggesting that the phase transitions may actually be not fully suppressed. Interestingly, a substantial contribution of unusual monoclinic phase was also employed to better fit the PXRD data of both compounds, which may again be related to the unaccounted phase separation at these mixed compositions. Note that López et al.^371^ also did not observe any symmetry lowering in the synchrotron XRD data down to 120 K for the *x* = 1/3 sample, which was obtained using mechanochemical synthesis.

The same work by Shahrokhi et al.^365^ also reported the dielectric properties of the *x* = 0, 1/3, 2/3, and 1 compounds (Figure 19c). As expected, pure MAPbI_3_ and MAPbBr_3_ perovskites demonstrated a sudden decrease of the dielectric permittivity related to the MA cation ordering, as the materials transitioned into the orthorhombic phase.^183,340,341^ In contrast, the temperature dependence of the dielectric permittivity of the mixed compositions revealed a broad featureless peak extending below 100 K (Figure 19c). Note that a very similar dielectric response was also obtained for the A-site mixing (see Figure 17), indicating that both approaches affect the MA cation dynamics in a similar way. In addition, as pointed by the authors, such a dielectric response might be a signature of a dipolar (orientational) glass phase.

The MA cation dynamics in the mixed MAPb(I_0.4_Br_0.6_)_3_ film were investigated by Selig et al.^369^ using 2D IR spectroscopy and classical MD simulations. Note that the studied *x* = 0.6 composition should be deep within the miscibility gap (Figure 19a), which was not discussed by the authors. As found by Lehmann et al.,^367^ such a bromine concentration should result in phase clustering with partial mixing. The 2D IR spectroscopy of pure MAPbI_3_ and MAPbBr_3_ perovskites revealed wobbling and reorientation dynamics of the MA cations occurring on the 0.3 ps and 1.5–3 ps time scale, respectively. In the mixed compound, the wobbling dynamics remained the same, while the time scale of the cation reorientation increased significantly up to 15 ps. This indicates hindering of the MA cation motion in the mixed halide system likely caused by the local distortions of the inorganic framework. The experimental results were also supported by the MD simulations, which also revealed partial suppression of the MA dynamics in the mixed compound. Similar MD simulation results were also obtained for the *x* = 1/3 and 2/3 compositions in the aforementioned study by Shahrokhi et al.,^365^ while Grüninger et al.^206^ observed restriction in the cation mobility due to halide mixing for the MA_0.15_FA_0.85_Pb(I_0.85_Br_0.15_)_3_ composition. Note that the 2D IR spectroscopy of the MA_1–*x*_Cs_*x*_PbI_3_ (*x* ≤ 0.3) mixture revealed a very similar change of the wobbling and jumping dynamics of the MA cations upon introduction of Cs^+^ ions.^344^

#### 5.1.2 **MAPb(I**_**1–***x*_**Cl**_***x***_**)**_**3**_

Due to an even larger difference in the ionic radii of I^–^ and Cl^–^ anions (220 pm vs 181 pm),^362^ an even wider miscibility gap is expected for the mixed MAPb(I_1–*x*_Cl_*x*_)_3_ system. Among the first studies of these compounds, Colella et al.^372^ reported a maximum chlorine content of *x* = 0.04, while somewhat higher incorporation up to *x* = 0.1 was stated by Unger et al.^373^ (both solvent synthesis). Pistor et al. reported the miscibility gap of 0.05 < *x* < 0.5.^374^ In a more recent work published by the Schorr group,^375^ the miscibility gap was refined using high-resolution synchrotron PXRD to be 0.03 < *x* < 0.99. Note that no deviation of symmetry from the parent compounds was observed in these weakly mixed compositions.

The same group also used inelastic and quasielastic neutron scattering to study the MA dynamics in the *x* = 0.02 compound.^376^ Upon introduction of chlorine, a significantly faster *C*_3_ reorientation around the C–N bond of the MA cations in the orthorhombic phase was observed compared to pure MAPbI_3_ (485 ps vs 1635 ps). In addition, mixing also decreased the activation energy of this motion from 41 to 22 meV. As discussed by the authors, these findings indicate that even a minute amount of chlorine is sufficient to substantially weaken the H-bonds between the MA cations and inorganic framework. In a recent study, the same group also reported that the temperature of the tetragonal-orthorhombic phase transition is lowered in the mixed composition (*x* = 0.02) by about 5 K compared to MAPbI_3_, indicating that a small amount of mixing also affects the structural phase transitions.^377^

#### 5.1.3 **MAPb(Br**_**1–*****x***_**Cl**_***x***_**)**_**3**_

In contrast to mixing with iodine, no evidence of the miscibility gap was found for the mixed MAPb(Br_1–*x*_Cl_*x*_)_3_ perovskites due to more similar radii of Br^–^ and Cl^–^ anions (196 pm vs 181 pm).^362^^364,368,369,377−382^ Several studies observed cubic symmetry at room temperature for different compositions covering the whole mixing interval^364,380,381,383^ in agreement with the symmetry of the end members (see Figure 3).

A cubic symmetry of the *x* = 1/3, 1/2, 2/3 mixed compositions at room temperature was also observed by López et al. using neutron powder diffraction.^379^ In addition, the authors also determined the preferred MA cation orientation in the inorganic cage, which was found to change from the [110] to [111] and finally to the [100] direction, as the chlorine content increased, and the size of the unit cell shrunk. The change of preferred directions also indicates the reduction in the degrees of freedom of MA cation, as the delocalization along the [110], [111], and [100] axis involves 6, 4, and 3 possible orientations, respectively.

The phase transitions in the mixed MAPb(Br_1–*x*_Cl_*x*_)_3_ system were first investigated by Alvarez-Galván et al. using synchrotron XRD.^380^ The authors observed a complete suppression of the phase transitions for the studied highly mixed compositions (*x* = 1/2 and 2/3) and stabilization of the cubic *Pm*3̅*m* phase.

**Figure 20:**
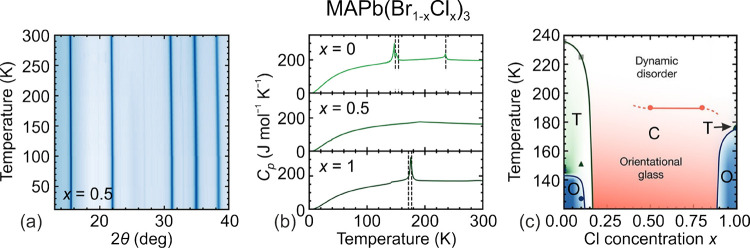
(a) PXRD pattern of the MAPb(Br_0.5_Cl_0.5_)_3_ composition showing a complete suppression of the phase transitions. (b) Temperature dependence of the heat capacity for the *x* = 0, 0.5, and 1 compositions. (c) Temperature–composition phase diagram of the MAPb(Br_1–*x*_Cl_*x*_)_3_ system. Symmetry notation: C - cubic, T - tetragonal, O - orthorhombic. Reprinted with permission from ref (381). Copyright 2021 American Chemical Society.

A similar behavior was also obtained in a recent multitechnique study by van de Goor,^381^ where a series of mixed compounds was investigated covering the whole range of compositions. A complete suppression of the phase transitions was obtained for the 0.2 < *x* < 0.8 compositions as evident from the PXRD and heat capacity measurements (Figure 20a,b). The authors also determined the phase diagram of this mixed system using the temperature dependence of the lattice microstrain extracted from the PXRD results (Figure 20c). Interestingly, for the highly mixed compositions, the microstrain value remained constant throughout the measured temperature range. This was attributed to the formation of the orientational glass phase caused by the halide disorder, which in turn distorts the octahedra and affects the MA cation motion. In a recent study, Naqvi et al. also observed local inhomogeneous environments of the MA cations in the mixed compositions of MAPb(Br_1–*x*_Cl_*x*_)_3_ using Raman spectroscopy.^377^ Note that a similar mechanism of the glassy phase formation was also proposed for the mixed A-site systems such as MA_1–*x*_DMA_*x*_PbBr_3_.^164^

The MA cation dynamics in the mixed MAPb(Br_0.4_Cl_0.6_)_3_ compound was also studied in the aforementioned work by Selig et al.^369^ using the 2D IR spectroscopy and classical MD simulations. Similarly to the mixed MAPb(I_0.4_Br_0.6_)_3_ composition (Figure 19d), the MAPb(Br_0.4_Cl_0.6_)_3_ film sample also showed a substantial increase of the MA cation reorientation time to 5 ps in contrast to much shorter time scales observed for the end members (1.5 ps for *x* = 0, and 1.2 ps for *x* = 1).

### 5.2 Cs-Based Compounds

#### 5.2.1 **CsPb(I**_**1–*****x***_**Br**_***x***_**)**_**3**_

Several studies revealed no miscibility gap in the mixed inorganic CsPb(I_1–*x*_Br_*x*_)_3_ perovskite system,^232,384−386^ although Wang et al. reported the onset of phase segregation under light illumination.^387^ Many works also reported that introduction of bromine suppresses the yellow phase and stabilizes the photoactive black phase of CsPbI_3_.^232,384−386,388,389^

**Figure 21:**
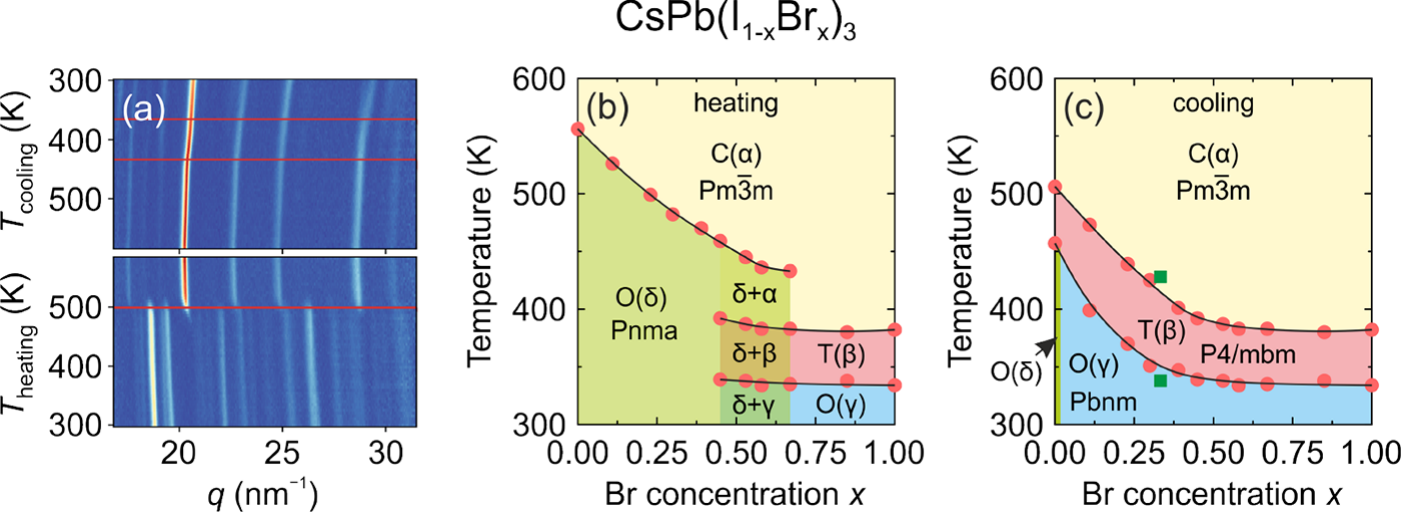
(a) Grazing-incidence wide-angle X-ray scattering pattern of the printed CsPb(I_0.77_Br_0.23_)_3_ thin film sample obtained on heating and then subsequent cooling runs. Red lines indicate the phase transitions. Temperature–composition phase diagram of the CsPb(I_1–*x*_Br_*x*_)_3_ system obtained on (b) heating and (c) subsequent cooling. Symmetry notation: C - cubic, T - tetragonal, O - orthorhombic. Green data points in (c) are taken from ref (388). Reprinted (adapted) with permission from ref (384). Copyright 2020 The Royal Society of Chemistry.

Näsström et al.^384^ used in situ grazing-incidence wide-angle X-ray scattering and X-ray fluorescence experiments to investigate structural phase transitions and phase diagrams for a broad range of compositions of the CsPb(I_1–*x*_Br_*x*_)_3_ thin films obtained by inkjet printing and ordinary coating (for Br-rich compositions). The compositions containing up to moderate amounts of bromine (*x* ≤ 0.45) were found to form the orthorhombic (*Pnma*) yellow δ-phase at room temperature. This phase was converted to the cubic  black α-phase upon heating to high temperatures (Figure 21a), and the temperature of this transition decreased significantly with increasing *x*, indicating stabilization of the cubic phase (Figure 21b). A subsequent cooling of the mixed compounds showed a contrasting behavior compared to heating, as, instead of the yellow phase formation, a phase transition sequence (cubic-tetragonal-orthorhombic) of the black polymorph was observed (Figure 21c). Note that pure CsPbI_3_ perovskite was an exception exhibiting an immediate conversion back to the yellow phase (Figure 21c). On the opposite side of the phase diagram, Br-rich compositions (*x* ≥ 0.85) already crystallized into the orthorhombic (*Pbnm*) black γ-phase (Figure 21b) resulting in the same behavior during heating and cooling. Interestingly, for the intermediate values of mixing, the coexistence of the yellow and black phases was observed at room temperature prior the heating run (Figure 21b). Note that similar phase transition temperatures of the black polymorph were also observed for the CsPb(I_2/3_Br_1/3_)_3_ thin film by Breniaux et al.^388^ (Figure 21c). A gradual evolution of the phase transitions with varying composition is in a sharp contrast to the MA-based systems, where phase transitions are typically suppressed.^164−166,336,381^

#### 5.2.2 **CsPb(Br**_**1–***x*_**Cl**_***x***_**)**_**3**_

This mixed system was investigated in the already discussed study by van de Goor et al.^381^ using PXRD and heat capacity measurements assisted by DFT calculations. In contrast to the hybrid MAPb(Br_1–*x*_Cl_*x*_)_3_ system (Figure 20), the authors did not observe lattice stabilization and formation of the orientational glass phase even for the highest mixing level (*x* = 0.5), as the system remained in the orthorhombic (*Pnma*) symmetry inherited from the end member compositions. The DFT calculations also revealed orthorhombic distortion and a narrow distribution of the Pb–halide–Pb angle, indicating preservation of the long-range order.

This may be related to the fact that in both CsPbBr_3_ and CsPbCl_3_, end members’ discernible octahedral distortions are evident within the orthorhombic *Pnma* structures, as indicated by the Pb–X–Pb angles distributed in the range of 157–165° and 160–169°, respectively.^287,288^ This observation stands in contrast to MAPb(Br_1–*x*_Cl_*x*_)_3_, wherein the energetically favored structural configuration is an average cubic one, characterized by a broad Pb–halide–Pb angle distribution that is also shifted toward the theoretically ideal cubic value of 180°. A contributing factor to the absence of long-range order may be attributed to the disparate degrees of distortion observed in nonmixed compositions. In MAPbBr_3_, for instance, all Pb–Br–Pb angles demonstrate a near-equivalence, fluctuating between 166.8° and 167.3°,^269^ while Pb–Cl–Pb angles in MAPbCl_3_ exhibit a widespread distribution in the range of 155–170°.^390^

### 5.3 MHy-Based Compounds

Unlike archetypal MAPbX_3_ and FAPbX_3_ compounds, MHyPbX_3_ (X = Br, Cl) 3D perovskites show exceptionally distorted and noncentrosymmetric framework consisting of alternating weakly and strongly distorted PbX_6_ octahedra.^184,185^ The MHyPbBr_3_ compound exhibits a single structural phase transition from the cubic to monoclinic phase at 418 K, while MHyPbCl_3_ also shows a single transition from the orthorhombic to monoclinic symmetry at 342 K (Figure 3). Note that the iodide analogue does not form a 3D perovskite and instead crystallizes into a 2D layered structure.^391^

#### 5.3.1 **MHyPb(Br**_**1–***x*_**Cl**_***x***_**)**_**3**_

**Figure 22:**
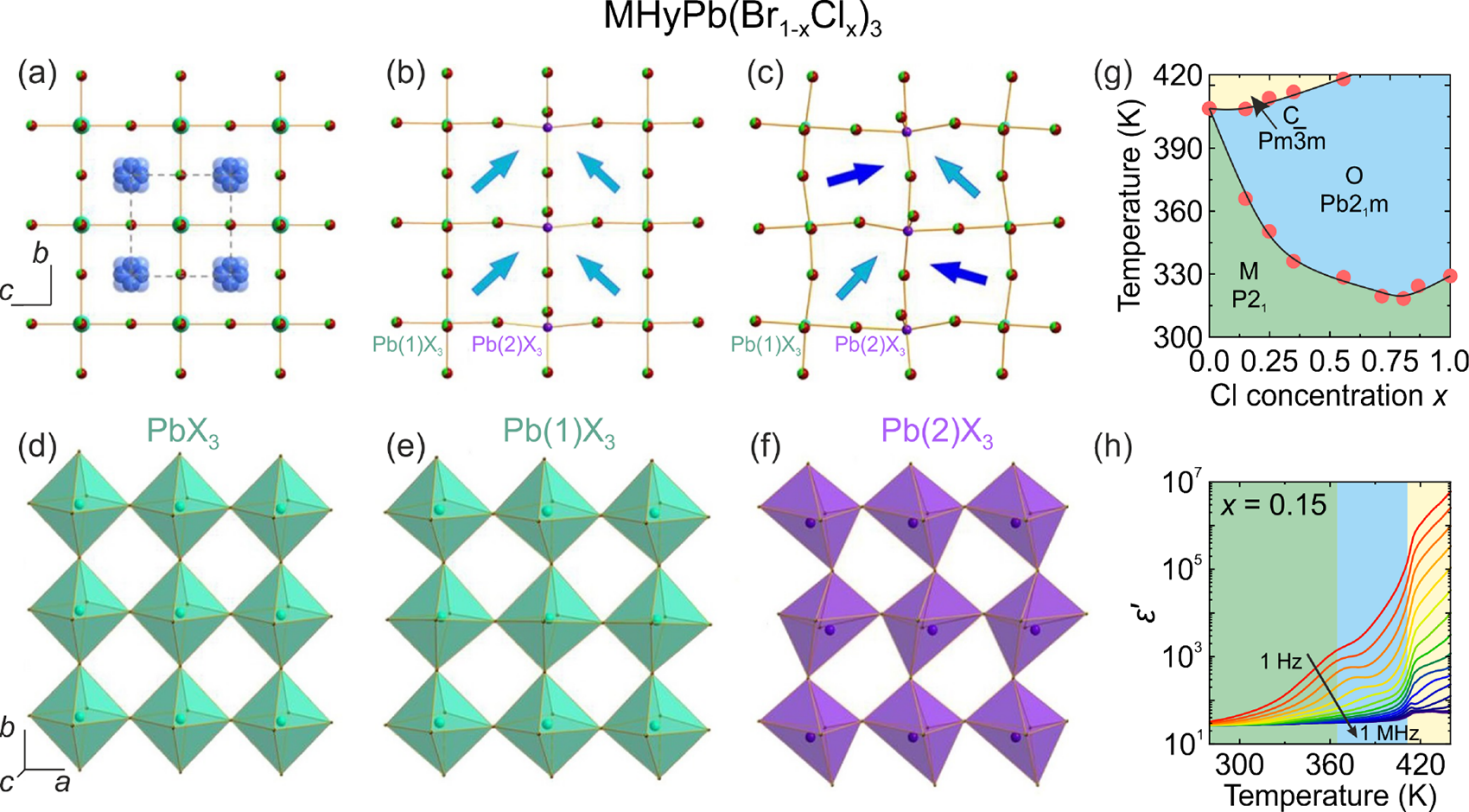
Crystal structure of the mixed MHyPb(Br_1–*x*_Cl_*x*_)_3_ system in the (a) cubic, (b) orthorhombic, and (c) monoclinic phases. Arrows represent electric dipole moments of MHy cations. (d) Single [100] layer of the PbX_6_ octahedra in the cubic phase. [100] layers of (e) less-distorted Pb(1)X_6_ and (f) highly distorted Pb(2)X_6_ octahedra in both polar orthorhombic and monoclinic phases. (g) Temperature–composition phase diagram of the MHyPb(Br_1–*x*_Cl_*x*_)_3_ system. Symmetry notation: C - cubic, O - orthorhombic, M - monoclinic. (h) Temperature dependence of the real part ε′ of the complex dielectric permittivity of the MHyPb(Br_0.85_Cl_0.15_)_3_ pellet sample. The dielectric response is dominated by the conductivity effects. Reprinted (adapted) with permission from ref (118). Copyright 2022 American Chemical Society.

Halide mixing effects in the MHy-based hybrid perovskites were recently studied by some of us using a suite of different experimental techniques including SCXRD, DSC, SHG, and DS.^118^ No evidence of the miscibility gap was observed in the whole range of mixing in agreement with the related MAPb(Br_1–*x*_Cl_*x*_)_3_ system.^381^ The SCXRD experiments of MHyPb(Br_1–*x*_Cl_*x*_)_3_ revealed that mixing does not change the polar symmetry, MHy cation arrangement, and framework distortions of the corresponding structural phases (see Figure 22a–f). The DSC experiments were used to follow the phase transition temperatures with mixing as summarized in the phase diagram presented in Figure 22g. Upon an increase of *x*, the stability region of the orthorhombic *Pb*2_1_*m* phase gradually increased at the expense of the cubic *Pm*3̅*m* and monoclinic *P*2_1_ phases.

Note that no lattice symmetrization (phase transition suppression) was observed in the mixed compositions of MHyPb(Br_1–*x*_Cl_*x*_)_3_ in contrast to the MA-based system (Figure 20c), where signatures of the glassy behavior were detected. In addition, no substantial broadening of the transition anomalies was observed in MHyPb(Br_1–*x*_Cl_*x*_)_3_ upon mixing. These results indicate that introduction of Cl^–^ anions did not destroy the long-range order in this system, and thus lattice symmetrization and formation of the dipolar glass phase cannot be expected. This was also evident in the dielectric responses of the mixed compounds, which were dominated by the conductivity processes (Figure 22h). The absence of the lattice symmetrization may be explained by the already significantly deformed and rigid inorganic framework at room temperature. Note that a similar behavior was also observed for the related CsPb(Br_1–*x*_Cl_*x*_)_3_ system.^381^

#### 5.3.2 **MHyPb(Br**_**1–***x*_**I**_***x***_**)**_**3**_

In the same study,^118^ an attempt was made to incorporate iodine in the structure of MHyPbBr_3_ perovskite using solvent synthesis. A MHyPb(Br_0.93_l_0.07_)_3_ composition of 3D perovskite was obtained as revealed by energy-dispersive X-ray spectroscopy analysis. SCXRD and DSC experiments showed that this compound undergoes a structural phase transition at 390 K from the cubic *Pm*3̅*m* to monoclinic *P*2_1_ phase, both inherited from pure MHyPbBr_3_.

### 5.4 Summary

Several important observations can be made from the reviewed results of mixed-halide lead halide perovskites. The mixed compositions with iodine, which has the largest ionic radius, may result in a miscibility gap, which is especially pronounced for the MA-based compounds obtained using the solvent synthesis. Interestingly, it seems that this gap can be reduced or fully suppressed using mechanochemical synthesis. There is no miscibility gap for the mixed Br/Cl compounds allowing one to fully chart the phase diagrams.

**Figure 23:**
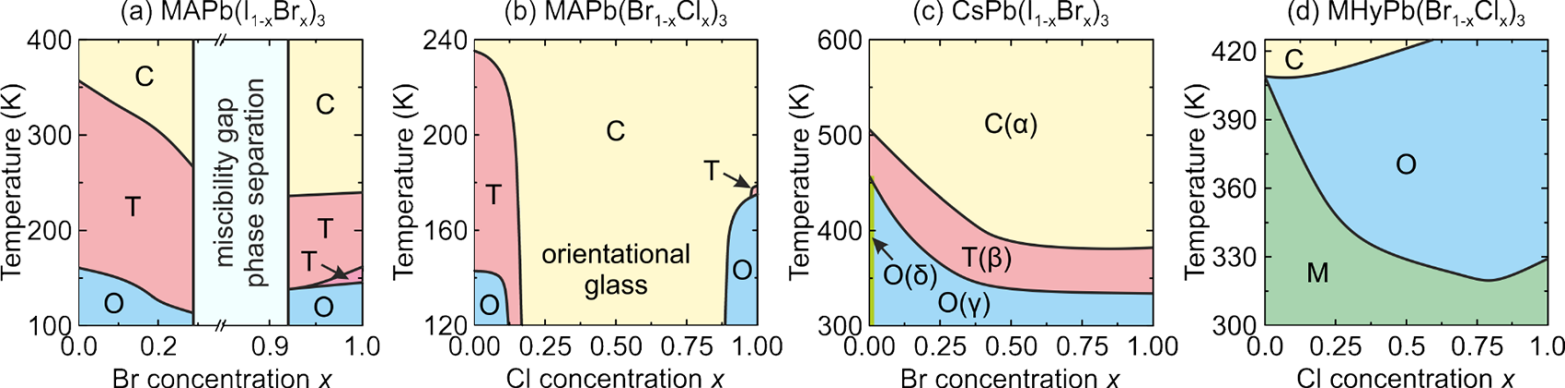
Comparison of the temperature–composition phase diagrams of the mixed-halide (a) MAPb(I_1–*x*_Br_*x*_)_3_, (b) MAPb(Br_1–*x*_Cl_*x*_)_3_, (c) CsPb(I_1–*x*_Br_*x*_)_3_ (black phase except for *x* = 0), and (d) MHyPb(Br_1–*x*_Cl_*x*_)_3_ systems. Symmetry notation: C - cubic, T - tetragonal, O - orthorhombic, M - monoclinic. (a) Adapted with permission from ref (367). Copyright 2019 The Royal Society of Chemistry. (b) Adapted with permission from ref (381). Copyright 2021 American Chemical Society. (c) Adapted with permission from ref (384). Copyright 2020 The Royal Society of Chemistry. (d) Adapted with permission from ref (118). Copyright 2022 American Chemical Society.

The temperature–composition phase diagrams of the mixed MAPb(I_1–*x*_Br_*x*_)_3_, MAPb(Br_1–*x*_Cl_*x*_)_3_, CsPb(I_1–*x*_Br_*x*_)_3_, and MHyPb(Br_1–*x*_Cl_*x*_)_3_ systems are summarized in Figure 23 revealing vastly different behaviors. MAPb(I_1–*x*_Br_*x*_)_3_ compounds obtained using solvent synthesis exhibited a wide region of phase separation, while well-mixed compositions outside this gap showed a significant lowering of the transition temperatures upon mixing^367^ (Figure 23a). This indicates increased stability of the cubic phase due to the disorder induced by random halide distribution, while the dielectric response obtained for the high levels of mixing showed signatures of the glassy phase formation.^365^ The phase diagram of the MAPb(Br_1–*x*_Cl_*x*_)_3_ system, which has no miscibility gap, revealed a complete suppression of the phase transitions and symmetrization of the lattice for the highly mixed compounds (Figure 23b).^381^ For these compositions, the formation of the orientational glass phase was also observed.

The phase diagrams and effects of mixing are qualitatively different when mixed compositions are obtained from the end members, which exhibit distorted lower symmetry structures already at room temperature such as Cs-based (orthorhombic) and MHy-based (monoclinic) compounds. In both cases, no suppression of the phase transitions and lattice symmetrization were observed, as systems gradually evolved with halide mixing maintaining sharp phase transition anomalies (Figure 23c,d). This indicates preservation of the long-range order and absence of the glassy phase. Such a behavior may be explained by a much higher stability of the distorted phases (much higher transition temperatures) compared to the MA-based perovskites.

## 6 Simultaneous A- and X-Site Mixing in 3D Perovskites

Here, we discuss how simultaneous mixing at the A- and X-sites affects the structural phase transitions and cations dynamics in 3D lead halide perovskites. The reported studies concentrate on the highly promising FA_1–*x*_MA_*x*_Pb(I_1–*y*_Br_*y*_)_3_ and FA_1–*x*_Cs_*x*_Pb(I_1–*y*_Br_*y*_)_3_ systems, where FA is the dominant molecular cation.

### 6.1 **FA**_**1–***x*_**MA**_***x***_**Pb(I**_**1–***y*_**Br**_***y***_**)**_**3**_

**Figure 24:**
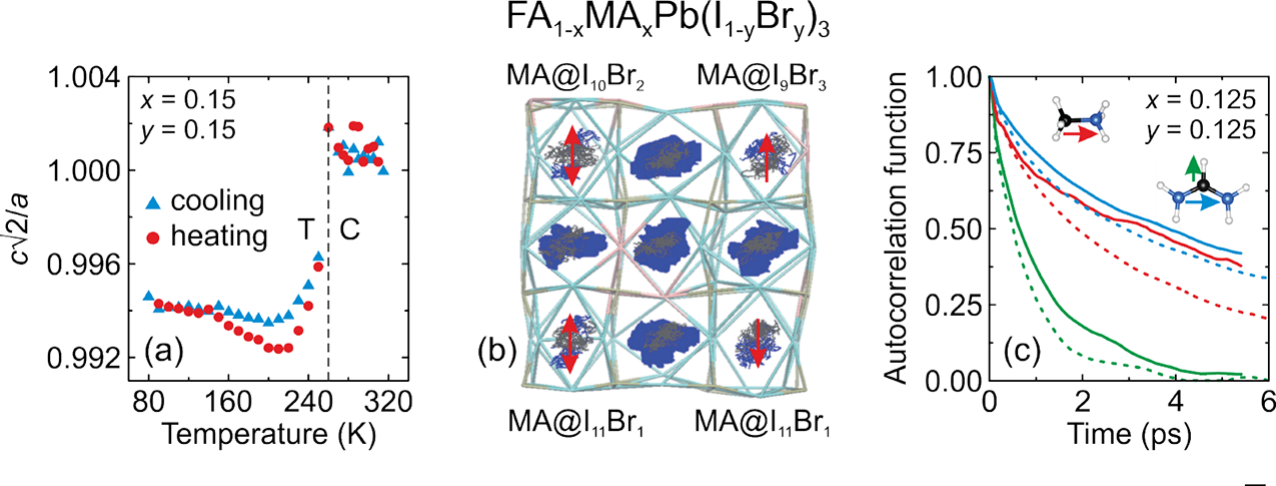
(a) Temperature dependence of the reduced lattice parameter ratio  of the mixed FA_0.85_MA_0.15_Pb(I_0.85_Br_0.15_)_3_ system obtained on cooling and heating. (b) Trajectories of the MA and FA cations in the mixed FA_0.875_MA_0.125_Pb(I_0.875_Br_0.125_)_3_ perovskite obtained by the *ab initio* MD. Arrows indicate average direction of the MA cations. (c) Simulated vector autocorrelation functions of the MA and FA cations (insets) in the same solid solution (solid curves) compared with simulations obtained in the parent MAPbBr_3_ and FAPbI_3_ compounds (dashed curves). (a) Adapted with permission from ref (392). Copyright 2020 Wiley-VCH. (b, c) Reprinted (adapted) with permission from ref (393). Copyright 2022 The Royal Society of Chemistry.

Greenland et al. used temperature-dependent PXRD to study the structural phase transitions in the FA_0.85_MA_0.15_Pb(I_0.85_Br_0.15_)_3_ thin film sample.^392^ The phase transition from the cubic  to tetragonal (*P*4/*mbm*) symmetry was observed at 260 K (Figure 24a), which is about 15 K lower compared to pure FAPbI_3_ (Figure 3). In addition, the lowering of the phase transition temperature seems to be more pronounced compared to the FA_0.85_MA_0.15_PbI_3_ composition, where mixing was done only at the A-site (see phase diagram in Figure 5). The authors also claim observation of the phase transition to the γ-phase, which occurred at 90 K instead of about 150 K observed for pure FAPbI_3_. Interestingly, upon heating, the PXRD peaks of this low-temperature phase persisted up to 220 K, indicating a very broad hysteresis (also visible in Figure 24a).

Menéndez-Proupin et al. employed *ab initio* MD to study molecular cation dynamics in the FA_0.875_MA_0.125_Pb(I_0.875_Br_0.125_)_3_ perovskite.^393^ The density maps of the C and N atoms revealed that the FA cations rotate freely in the lattice, while the MA cations have a unique orientation during the calculated trajectory (Figure 24b), which seems to be independent of the number of neighboring Br^–^ anions. The authors also calculated the vector autocorrelation function for both molecular cations showing that in the highly mixed composition the dynamics of both cations are slower (Figure 24c). Note that the same behavior was obtained in the mixed FA_0.9_Cs_0.1_PbI_3_ (Figure 15c)^226^ and MAPb(I_0.5_Br_0.5_)_3_ systems (Figure 19d).^369^

Johnston et al. used QENS to study molecular cation dynamics in the highly mixed FA_0.8_MA_0.15_Cs_0.05_Pb(I_1–*y*_Br_*y*_)_3_ (*y* = 0, 0.1, 0.15, and 0.2) system with an addition of a small amount of Cs^+^ cations at the A-site.^394^ The temperature-dependent mean-squared displacement data revealed a phase transition to the low-temperature phase at about 100 K independent of the halide mixing. This result does not agree with the phase diagram of the mixed MA_1–*x*_FA_*x*_PbI_3_ system (*y* = 0) (Figure 5), suggesting that Cs^+^ cations may significantly affect the phase transitions. The authors also observed the strongest suppression of the molecular cation dynamics (particularly FA) for the *y* = 0.15 composition.

Xie et al.^395^ grew high-quality single crystals of (FAPbI_3_)_1–*x*_(MAPbBr_3_)_*x*_. They observed that the best composition for a black phase without segregation is *x* = 0.1–0.15 and that the incorporation of MA is essential for stabilization of the black phase from the thermodynamics point of view. Namely, incorporation of MA tunes the tolerance factor toward the ideal value of 1 and lowers the Gibbs free energy via unit cell contraction and cation disorder. On the other hand, Br incorporation also stabilizes the black phase but from the kinetics point of view, because this anion controls the crystallization kinetics and reduces defect density.

### 6.2 **FA**_**1–*****x***_**Cs**_***x***_**Pb(I**_**1–***y*_**Br**_***y***_**)**_**3**_

Barrier et al.^396^ used synchrotron X-ray diffraction to probe the evolution of crystal structure across the tetragonal-cubic phase transition for FA_1–*x*_Cs_*x*_Pb(I_1–*y*_Br_*y*_)_3_ thin films with *x* = 0.17–0.4 and *y* = 0.05–0.3. By analysis of the octahedral tilt angle, they observed that the cubic-tetragonal phase transition occurred over temperature range on the order of 40 °C. This behavior proved that the samples were poorly mixed and contained coexisting cubic and tetragonal regions. It has been discussed that the compositional heterogeneity can have two origins. The first one is due to the thermodynamic separation of the mixed perovskite into two or more distinct phases driven by minimization of the free energy, whereas the second one likely results from kinetics due to the solution-processing methods used to fabricate thin films. The authors have made no attempt to distinguish between these components but emphasized that it is important to take into account the presence of heterogeneity, since it may lead to chemical, structural, and electronic property heterogeneity on multiple length scales.

## 7 B-Site Mixing in 3D Perovskites

The number of works reporting on how the B-site mixing affects the structural and dynamic properties of hybrid perovskites is highly limited. Here, we discuss two MAPbI_3_ and FAPbI_3_ perovskite systems, where lead is partially replaced by tin.

### 7.1 **MAPb**_**1–*****x***_**Sn**_***x***_**I**_**3**_

The room-temperature structural properties of the mixed MAPb_1–*x*_Sn_*x*_I_3_ system were investigated by Kanatzidis group in two subsequent studies.^93,94^ The authors observed a full compatibility of both parent compounds, as no solubility limit in the mixed perovskites was detected. For all compositions, the room-temperature crystal structure adopted tetragonal symmetry. For the Sn-rich compositions (*x* ≥ 0.5), the *P*4*mm* space group was obtained, while the *x* < 0.5 compounds including MAPbI_3_ were indexed using the noncentrosymmetric *I*4*cm* space group instead of the expected centrosymmetric *I*4/*mcm*. The absence of the centrosymmetry was not discussed or proved by the authors making this assignment questionable.

### 7.2 **FAPb**_**1–*****x***_**Sn**_***x***_**I**_**3**_

**Figure 25:**
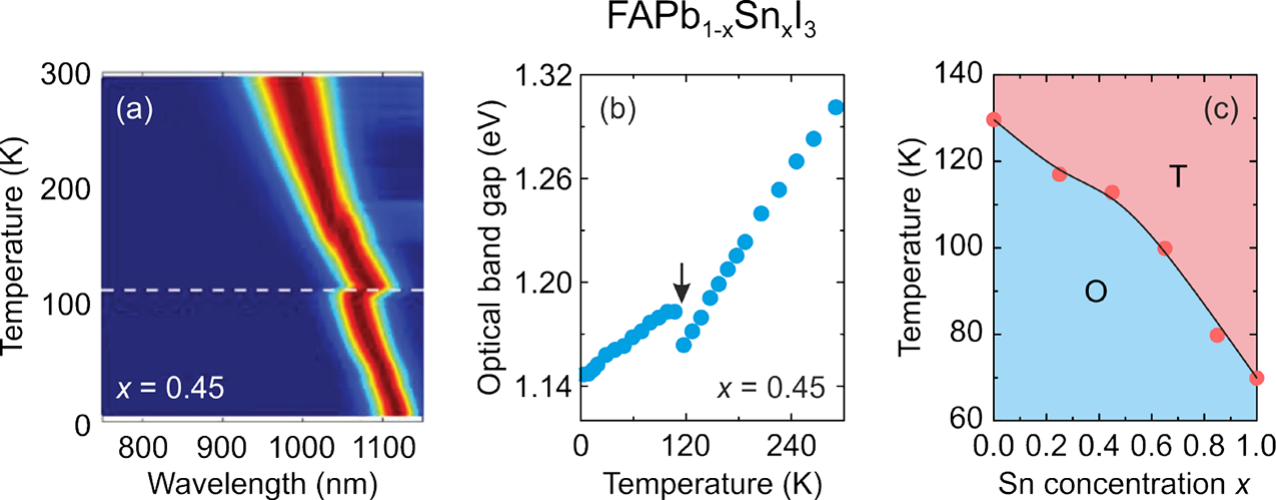
(a) Temperature dependence of the PL signal of FAPb_0.55_Sn_0.45_I_3_ perovskite. (b) Temperature dependence of the optical band gap determined from the PL data of the same sample. Arrow marks the phase transition point. (c) Temperature–composition phase diagram of the tetragonal–orthorhombic phase transition in FAPb_1–*x*_Sn_*x*_I_3_ system. Reprinted (adapted) with permission from ref (89). Copyright 2018 Wiley-VCH.

Parrott et al.^89^ reported a more detailed investigation of the mixing effects in the related FAPb_1–*x*_Sn_*x*_I_3_ perovskites, where no solubility limit was also observed. The authors used temperature-dependent PL and light absorption experiments to probe how introduction of tin affects the phase transitions (Figure 25a) and optical properties such as optical band gap (Figure 25b). Clear anomalies were observed in the measured data at the low-temperature phase transition point allowing construction of the phase diagram (Figure 25c). The observed transition likely corresponds to the tetragonal-orthorhombic symmetry change based on the symmetry of the parent compounds at such temperatures (Figure 3).^170^

In the aforementioned piezoresponse force microscopy study by Ahmadi et al.,^339^ the authors observed that, similarly to MA_0.15_FA_0.85_PbI_3_, the FAPb_0.85_Sn_0.15_I_3_ compound also exhibits ferroelectric domain-like structures, however, much weaker than expected for classical ferroelectric domains.

Further studies with the emphasis on the structural phase transitions and dynamics are necessary to provide a more detailed picture of the B-site mixing effects.

## 8 Halide, Metal, and Cation (A-, A′-, A″-Site) Mixing in Lower-Dimensional Perovskites and Related Compounds

Here, we discuss how mixing affects the structural and dynamic properties of low dimensional lead halide perovskites. Note that the number of works reporting on these effects is significantly lower compared to the 3D APbX_3_ compounds.

### 8.1 **CHA**_**2**_**Pb(Br**_**1–*****x***_**I**_***x***_**)**_**4**_

**Figure 26:**
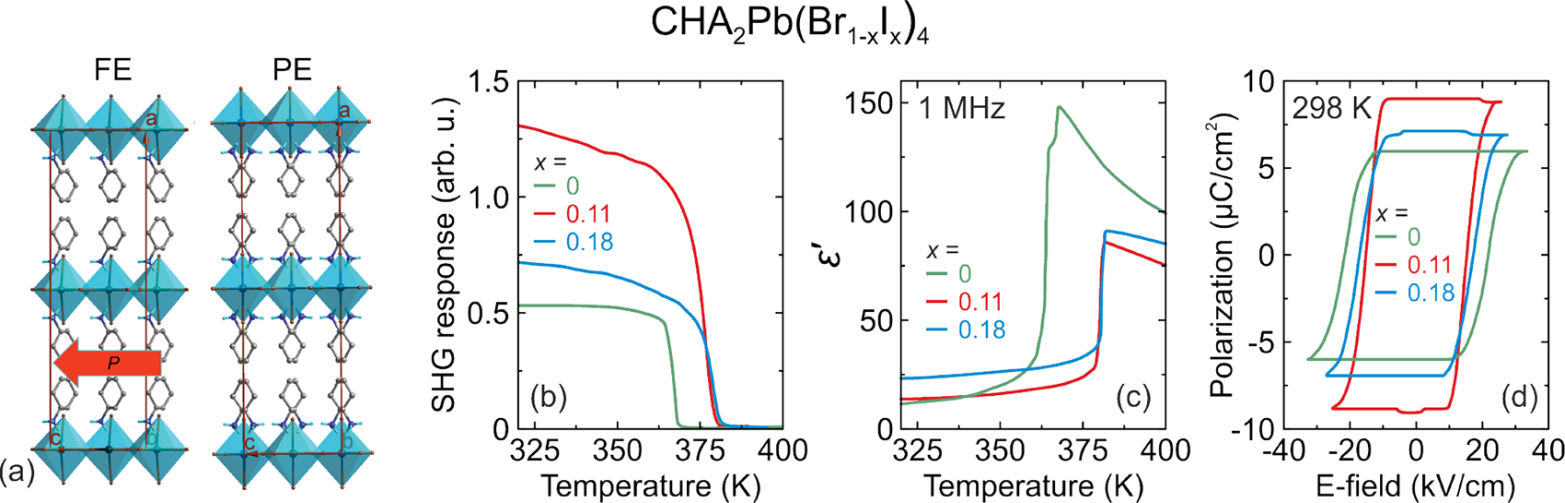
(a) Crystal structure of pure CHA_2_PbBr_4_ compound in the ferroelectric (FE) and paraelectric (PE) phases. Cation disorder is present in the PE phase. (b) Temperature dependence of the (b) SHG response and (c) real part ε′ of the complex dielectric permittivity (1 MHz) of the mixed CHA_2_Pb(Br_1–*x*_I_*x*_)_4_ system (*x* = 0, 0.11, and 0.18). (d) Ferroelectric hysteresis loops of the same compounds obtained at room temperature. Reprinted (adapted) with permission from ref (397). Copyright 2016 Wiley-VCH.

Ye et al.^397^ used DSC, SHG, light absorption, dielectric and hysteresis loop measurements to probe how introduction of iodine up to *x* = 0.18 affects the phase transitions, band gap, ferroelectric and NLO properties of 2D layered perovskite comprising cyclohexylammonium (CHA) cations (Figure 26a). On cooling, pure CHA_2_PbBr_4_ undergoes a ferroelectric phase transition from the *Cmca* to the *Cmc*2_1_ phase at 363 K. The authors showed that mixing increased the phase transition temperature to 378 and 380 K for the *x* = 0.11 and 0.18 compositions, respectively. In addition, mixing also increased intensity of the SHG signal (Figure 26b) and narrowed the band gap from 3.05 eV for CHA_2_PbBr_4_ to 2.74 eV for CHA_2_Pb(Br_0.82_I_0.18_)_4_ while maintaining the phase transition. Interestingly, the dielectric anomaly associated with the phase transitions showed no broadening upon mixing (Figure 26c) in contrast to the majority of the 3D APbX_3_ perovskites (see e.g. Figure 19c). In addition, the ferroelectric properties also remained in the mixed compositions (Figure 26d) showing that the CHA cation ordering is decoupled from the halide mixing.

A full range of compositions (0 ≤ *x* ≤ 1) of the same system was studied by Yangui et al.^37^ It was shown that a composition-induced phase transition from the *Cmc*2_1_ to *Pbca* phase occurs for 0.5 < *x* < 0.6 at room temperature. The change in composition also led to the appearance of white-light emission for *x* < 0.5.

### 8.2 **(C**_**9**_**H**_**19**_**NH**_**3**_**)**_**2**_**PbI**_**2**_**Br**_**2**_

Abid et al.^398^ studied phase transition in (C_9_H_19_NH_3_)_2_PbI_2_Br_2_ solid solution, which crystallizes in a 2D perovskite structure. Using DSC, PXRD, and IR spectroscopy, they showed that the phase transition occurs around 230 K. The crystal structure below 230 K was not solved, but spectroscopic studies revealed that the phase transition is related to a decreased conformational disorder of the methylene units in the alkyl chains.

### 8.3 **Br-PEA**_**2**_**Pb(Br**_***x***_**Cl**_**1–***x*_**)**_**4**_

Pareja-Rivera et al.^40^ studied the effect of mixing at the X-site on the PL properties of 2D perovskite comprising 4-bromophenethylammonium (Br-PEA) cation. No solubility limit was observed, and the crystal structure of this system was found to be orthorhombic *Fmm*2 in the whole range of mixing. Analysis of the structural data revealed a much larger distortion of the inorganic layers for *x* = 0 compared to *x* = 1. Lattice parameters *b* and *c* increased with increasing Br content, whereas an opposite behavior was observed for the *a* parameter. Mixing at the X-site had a pronounced effect on the band gap and PL, as the band gap narrowed with increasing *x*, while PL for the *x* = 1 composition was dominated by a sharp violet emission with the peak maximum near 420 nm. This emission shifted to the UV range with decreasing *x*. When *x* decreased to about 0.6, a new broadband emission appeared near 500 nm, and its intensity increased on further lowering of *x*.

### 8.4 **PEA**_**2**_**Pb(Br**_***x***_**Cl**_**1–*****x***_**)**_**4**_**and PEA**_**2**_**Pb(Br**_***x***_**I**_**1–*****x***_**)**_**4**_

Mixing effects at the X-sites were also reported for 2D perovskites comprising the phenethylammoium (PEA) cation.^39,399,400^ Similarly to the Br-PEA analogue discussed above, Cai et al. claimed no solubility limit in the PEA_2_Pb(Br_*x*_Cl_1–*x*_)_4_ system, but the crystal symmetry was lower, triclinic *P*1̅.^39^

The XRD and DFT calculations revealed phase separation for the *x* = 0.25 composition of the mixed PEA_2_Pb(Br_*x*_I_1–*x*_)_4_ analogue, while no phase separation was observed for *x* ≥ 0.5.^399^ For *x* = 0.75, two PL bands were observed and only one for *x* = 0.25 and 0.5, which was attributed to different crystal configurations simultaneously present in the prepared film. Another study of PEA_2_Pb(Br_*x*_I_1–*x*_)_4_ system showed the presence of two (002) diffraction peaks for the *x* = 0.79 composition, suggesting an immiscible system with two discrete halide phases.^400^

### 8.5 **HA**_**2**_**Pb(Br**_***x***_**I**_**1–*****x***_**)**_**4**_**and HA**_**2**_**FAPb**_**2**_**(Br**_***x***_**I**_**1–*****x***_**)**_**7**_

A mixing effect at the X-sites was also reported for HA_2_Pb(Br_*x*_I_1–*x*_)_4_ and HA_2_FAPb_2_(Br_*x*_I_1–*x*_)_7_ compounds (HA = *n*-hexylammonium).^400^ The situation for these systems is very similar to that observed for PEA_2_Pb(Br_*x*_I_1–*x*_)_4_,^400^ as these halides do not show full solubility, and there is a clear crossover from near-pure-I to near-pure-Br phases for *x* = 0.84–0.88.^400^ The authors also showed that these systems may show photoinduced phase separation over hundreds of ms for focused laser beam and over minutes under the diffuse UV light.

### 8.6 **Et**_**3**_**PrNPb(Br**_**1–*****x***_**I**_***x***_**)**_**3**_

Shao et al.^124^ reported investigation of the mixing effects at the X-site of 1D hybrid lead halide comprising globular-shaped triethylpropylammonium (Et_3_PrN) cations. The authors found no solubility limit for this mixed halide system.

The DSC experiments showed that the phase transition is weakly affected by mixing as its temperature gradually decreased from 480 K for *x* = 0 to 445 K for *x* = 1. The entropy associated with the phase transition was the largest for pure Et_3_PrNPbBr_3_ (43.54 J mol^–1^ K^–1^) and decreased to 32.36 J mol^–1^ K^–1^ for Et_3_PrNPbI_3_ indicating a lower degree of ordering. The mixing with iodine also results in the shift of the band gap from 3.50 eV for *x* = 0 to 2.93 eV for *x* = 1.

### 8.7 **t-BA**_**2**_**Pb(Br**_**1–*****x***_**I**_***x***_**)**_**4**_

The mixed halide system containing *tert*-butylammonium (t-BA) branched spacer cation was recently studied by Ovčar et al.^36^ The authors showed that the end members crystallize into the polar *P*2_1_ structures: 1D for the iodide (t-BAPbI_3_), and layered 2D nonperovskite for the bromide (t-BAPb_2_Br_5_). Interestingly, the perovskite architecture was found to be stabilized in the mixed halide t-BA_2_PbBr_2_I_2_ system (2D layered perovskite structure, monoclinic *P*2_1_/*c* space group), however, at the expense of the polar symmetry.

### 8.8 **(3AMP)**_***x***_**(4AMP)**_**1–***x*_**(FA)**_***y***_**(MA)**_**1–***y*_**Pb**_**2**_**Br**_**7**_

Mixing of organic cations in a DJ type structure of (3AMP)_*x*_(4AMP)_1–*x*_(FA)_*y*_(MA)_1–*y*_Pb_2_Br_7_ (3AMP = 3-(aminomethyl)piperidinium, 4AMP = 4-(aminomethyl)piperidinium) was studied by Mao et al.^138^ This system showed a composition-induced phase transition from the monoclinic *Cm* symmetry for *x* = 1 and *y* = 1 to the monoclinic *Pc* phase for the (3AMP)_0.5_(4AMP)_0.5_FAPb_2_Br_7_,(3AMP)_0.5_(4AMP)_0.5_FA_0.5_MA_0.5_Pb_2_Br_7_, (4AMP)FA_0.5_MA_0.5_Pb_2_Br_7_, (4AMP)FAPb_2_Br_7_, and (3AMP)MAPb_2_Br_7_ compositions. The monoclinic *Cc* symmetry was reported for *x* = 0 and *y* = 0 as well as for *x* = 0.5 and *y* = 0. It was found that these structural changes have a significant impact on the Pb–Br–Pb bond angle and the band gap.

### 8.9 **(BA**_**0.5**_**PEA**_**0.5**_**)**_**2**_**MAPb**_**2**_**Br**_**7**_

Mixing of organic spacer cations was also studied in an RP type structure of (BA_0.5_PEA_0.5_)_2_MAPb_2_Br_7_.^136^ The structure of BA_2_MAPb_2_Br_7_ was refined in the orthorhombic *Ama*2 space group, and PEA_2_MAPb_2_Br_7_ was reported to be isostructural to PEA_2_MAPb_2_I_7_^136^ with the triclinic space group *P*1̅.^401^ The XRD results showed that (BA_0.5_PEA_0.5_)_2_MAPb_2_Br_7_ has a distinct structure from its parent compounds (triclinic *P*1).

### 8.10 **[AA**_***x***_**IdPA**_**1–*****x***_**]**_**2**_**MA**_***n*-1**_**Pb**_***n***_**I**_**3*n*+1**_ (*n* = 2–4)

Another reported RP system with mixing at the A′-sites is [AA_*x*_IdPA_1–*x*_]_2_MA_*n*-1_PbnI_3*n*+1_ (*n* = 2–4) family of compounds (AA = allylammonium, IdPA = iodopropylammonium).^402^ The most important conclusion from studies of this system is that, whereas the end members crystallize in the centrosymmetric structures, the mixed A′ phases are acentric. For instance, AA_2_MA_2_Pb_3_I_10_ and IdPA_2_MA_2_Pb_3_I_10_ crystallize in the *P*2_1_/*c* and *P*2/*c* structure, respectively, while [AA_*x*_IdPA_1–*x*_]_2_MA_2_Pb_3_I_10_ phases are described by the noncentrosymmetric *Pc* space group.^402^

### 8.11 **(BA)**_**2**_**(MA**_**1–*****x***_**EA**_***x***_**)**_**2**_**Pb**_**3**_**I**_**10**_

Mixing of organic cations in RP phases can also be realized at the “perovskitizer” sites, as demonstrated by Fu et al. in a study of (BA)_2_(MA_1–*x*_EA_*x*_)_2_Pb_3_I_10_.^140^ The end members BA_2_EA_2_Pb_3_I_10_ and BA_2_MA_2_Pb_3_I_10_ crystallize in the *Cmc*2_1_ and *C*2*cb* space groups, respectively, and, due to larger ionic radius of EA, BA_2_EA_2_Pb_3_I_10_ feature a higher degree of structural deformation. The authors showed that the symmetry of the mixed cation phases decreased to monoclinic *Cc*, and the average equatorial Pb–I–Pb bond angle of the inner layers strongly decreased with increasing EA fraction indicating higher structural distortion.

### 8.12 **(PEA)**_**2**_**(MA**_**1–*****x***_**GA**_***x***_**)**_**2**_**Pb**_**3**_**I**_**10**_

Another example of the RP phase with mixed cations at the “perovskitizer” sites is the (PEA)_2_(MA_1–*x*_GA_*x*_)_2_Pb_3_I_10_ system studied by Ramos-Terrón et al.^141^ In this work, thin films up to *x* = 0.5 were prepared, which is a much higher incorporation fraction compared to the 3D analogues (e.g., 25% in MA_1–*x*_GA_*x*_PbI_3_^69^). The authors observed shifts of diffraction peaks with increasing *x* due to enlargement of the unit cell. When the GA content approached 30%, a clear change in the width of the diffraction peaks was observed. This behavior was attributed to a sudden change in the arrangement of the mixed cations. To obtain information on the degree of lattice distortion induced by incorporation of GA, the DFT calculations were performed for the *x* = 0.25 and *x* = 0.5 compositions. The obtained results revealed low distortion of the inorganic layers on GA incorporation, even for a high GA content.

### 8.13 **(BA)**_**2**_**(MA**_**1–*****x***_**Cs**_***x***_**)Pb**_**2**_**Br**_**7**_

Mixing at the “perovskitizer” sites was also reported by Ma et al. for (BA)_2_(MA_1–*x*_Cs_*x*_)Pb_2_Br_7_.^403^ The authors grew single crystals for *x* = 1, 0.93, 0.79, and 0.66 and showed that incorporation of MA led to a decrease of the phase transition temperature from 411 K for *x* = 1 to 399, 383, and 379 K for *x* = 0.93, 0.79, and 0.66, respectively. For all solid solutions, the ferroelectricity of the low-temperature phase was confirmed by observation of the polarization hysteresis loops. The incorporation of MA led to narrowing of the band gap from 2.7 eV for *x* = 1 to 2.43 eV for *x* = 0.66. Most interestingly, the introduction of MA also resulted in a very large, at least 10-fold, enhancement of the photoactivity of this layered perovskite. This effect was attributed to the reduction of the exciton binding energy and enhanced ferroelectric polarization. As a result, the photoelectric activity of the mixed-cation crystals (*x* = 0.66) exceeded that of previously reported self-driven detectors based on hybrid lead halide perovskites.

### 8.14 **PEA**_**2**_**Pb**_**1–*****x***_**Sn**_***x***_**Br**_**4**_

Sui et al.^127^ reported investigation of the mixing effects at the B-site of 2D hybrid lead halide comprising aromatic PEA cations. This system shows a full solubility, while maintaining the same triclinic *P*1 crystal structure with decreased lattice parameters due to smaller ionic radius of Sn^2+^ (93 pm) compared to Pb^2+^ (120 pm). The smaller ionic radius of Sn^2+^ was also shown to be responsible for a shift of the band gap and PL peak to lower energies with increasing *x*.

In summary, literature data show that ion mixing in low-dimensional perovskites may significantly tune optoelectronic properties and lead to new symmetry of the mixed phases and loss of the inversion center.

## 9 Conclusions and Outlook

In this review, we provided a systematic and comprehensive picture of the mixing effects on the structural and dynamic properties of 3D and low-dimensional lead halide perovskites. The following important generalizations can be drawn from the reviewed studies:

(i) Frequently, the extent of mixing is limited by phase separation, which is especially pronounced, if the parent compounds have structures of different topologies. The phase separation directly affects the phase diagrams and material properties and thus must be carefully considered and determined. In general, for lead halide perovskites, kinetics often have a strong impact on the phases that form. This problem is especially important in the fabrication of thin films, since the spin-coating process often leads to pronounced heterogeneity.^361,395,396^ The presence of nanoscale local compositional variations may lead to structural and optoelectronic property heterogeneity on multiple length scales.^361,396^ It also complicates the phase transition behavior since slight variations in chemical composition can result in a different crystallographic phase being thermodynamically favorable and shift the transition temperature, leading to smearing of the transition anomaly.^361,396^ It has been shown that the A-site segregation can be often mitigated by annealing the samples either long enough or at a sufficiently high temperature.^361,396^ Another way is to perform rapid crystallization by antisolvent dripping.^404,405^ Trapping of the metastable phases can also be done by fast quenching.^264^

(ii) The mixing tends to partially or completely suppress the structural phase transitions resulting in the stabilization of the cubic phase and thus symmetrization of the crystal lattice, which is related to the improved stability, performance, and functionality of the mixed compounds.

(iii) Symmetrization of the lattice is frequently accompanied by signatures of the orientational (dipolar) glass phase, which occurs due to the multivalley potentials and frustrated interactions introduced by mixing and mediated by the local lattice deformations.

(iv) The symmetrization effect seems to be less pronounced or absent, if parent compounds exhibit highly stable low-symmetry phases at room temperature (e.g., Cs- and MHy-based perovskites). In this case, the evolution of the phase transitions with mixing is continuous, and signatures of the glassy phase are absent.

(v) The dynamics of molecular cations typically become slower upon mixing. This is especially pronounced at low temperature, where more bulky guest cations tend to freeze (e.g., DMA) and cause local deformation of the crystal lattice, which in turn increases the rotation barrier and hinders the dynamics of the smaller cations (e.g., MA).

(vi) Mixing in low-dimensional lead halide perovskites shows interesting behavior related to the occurrence of new crystal symmetries and loss of the inversion center. However, mixing in these compounds is significantly less studied compared to the 3D perovskites and therefore requires more attention. This is especially important in the context of ferroelectric low-dimensional perovskites, which upon mixing may form a ferroelectric relaxor phase instead of the dipolar glass.

Lastly, we identify other new promising systems and directions for further studies of the mixing effects on the structural and dynamic properties of lead halide and related perovskites. Such systems include hollow perovskites,^72^ deficient perovskites,^406^ and quasi 3D perovskites,^407^ all of which exhibit a substantial degree of disruption of the inorganic framework. It can be expected that in such compounds the molecular cation frustration would be even higher, as both organic and inorganic sublattices would significantly contribute to disorder.

Mixing in the newly discovered aziridinium-based 3D perovskites^237,298,408,409^ is also of high interest, especially from the stability point of view. As these perovskites have a 3D topology and high stability of the cubic phase, their solid solutions with high fractions of MA and FA should be feasible.

The B-site mixing in 3D perovskites is also significantly understudied compared to the A- and X-site mixing despite seemingly interesting effects on the structural phase transitions. In addition, mixing at the B-site should also significantly affect the off-centering dynamics of the inorganic framework,^342,410^ which should be directly visible in the low-temperature dielectric response of these materials. This is also related to lattice fluctuations resulting in low-symmetry correlated octahedral distortions observed in lead halide perovskites. It is very likely that mixing (especially at the B-site) would significantly affect such octahedral rotational instabilities. However, due to the requirement of advanced structural characterization techniques, such studies of the mixed systems are very rare.

As discussed, a major area of research in lead halide perovskites is related to the stabilization of the black photoactive phases of FAPbI_3_ and CsPbI_3_ compounds by utilizing several strategies including ion mixing—a topic which is out of scope for this review. However, the stabilization by mixing should inevitably affect the structural phase transitions and dynamics in the stabilized black phases of FAPbI_3_ and CsPbI_3_. One could expect a connection between the induced phase stability and suppression of the structural phase transitions in the black phases of these compounds. For example, Marshall et al. demonstrated a successful stabilization of the black phase of CsPbI_3_ by DMA cations reaching incorporation fractions up to 25%,^411^ while similar values of DMA proved to be sufficient to fully stabilize the cubic phase in the MA_1–*x*_DMA_*x*_PbBr_3_ system.^164^ Interestingly, a recent NMR study found that the mixed Cs_1–*x*_DMA_*x*_PbI_3_ perovskite phases only form when they are kinetically trapped by rapid antisolvent-induced crystallization.^404^ Of potential interest is also doping with Rb, which can be incorporated into the CsPbBr_3_, the black phase of CsPbI_3_, and CsPb(I/Br)_3_ solid solutions.^412^

As interesting example of the black phase stabilization was also recently reported by Duijnstee et al., where a small amount (*x* = 0.005) of tetrahydrotriazinium (THTZ-H) molecular cation was incorporated in the structure of FAPbI_3_.^413^ The samples were obtained from solutions containing methylenediammonium dichloride or hexamethylenetetramine, which then formed THTZ-H during the subsequent reactions with FA. Remarkably, the authors reported that such a mixing leads to an impressive enhancement of the black phase stability of FAPbI_3_ lasting for more than one year under ambient conditions. It can be expected that such a pronounced effect of THTZ-H on the black phase stabilization could also significantly alter the structural phase transitions and FA cation dynamics in α-FAPbI_3_.

The mixing effects and the dielectric response are also significantly understudied in the related lead-free families of hybrid materials such as tin halides,^297,414,415^ antimony halides,^416,417^ bismuth halides,^417−421^ tellurium halides,^422^ and double (e.g., Ag/Bi) perovskites.^423−427^ Many of these compounds undergo structural phase transitions related to the ordering-disordering phenomena and ferroelectric properties. Thus, one can expect a formation of similar frustrated phases in these materials upon mixing.

We note that, apart from dipolar glass, mixing-induced frustration may also lead to other exotic disordered phases. From the applications point of view, of particular interest is the relaxor phase,^58^ which is rather common in the mixed classical inorganic compounds. However, the formation of the relaxor phase requires mixing of ferroelectrics, while the ferroelectricity in 3D lead halide perovskites is questionable.^183^ On the other hand, numerous lower-dimensional perovskites are ferroelectric and antiferroelectric (see Table 2), and thus their mixing holds a significant potential for formation of relaxor and other complex frustrated phases.

There is presently a lot of interest in chiral layered lead halides, in which employment of a chiral organic cation leads to transfer of the chirality into the achiral inorganic framework.^428−435^ Due to the intrinsic noncentrosymmetry of chiral cations, chiral lead halides show unique and highly attractive features such as optical rotation, circular dichroism, circularly polarized PL, polarization-dependent SHG, ferroelectricity, bulk photovoltaic effect, chirality-induced spin selectivity, and topological quantum properties.^428−434^ Furthermore, the strong spin–orbit coupling of metal and halide ions imparts significant Rashba-Dresselhaus spin-splitting of otherwise 2-fold spin-degenerate electronic bands.^430,431,436^ We are not aware of any report on the mixing effect on phase transitions and lattice dynamics in chiral lead halides. However, there are some examples of mixed chiral hybrid perovskites reporting a significant tuning of optical properties upon mixing at the B- and X-sites.^433,437,438^ Thus, a substantial effect on the structural phase transitions and dynamics can be also expected.

Another intriguing feature of certain mixed inorganic ferroelectrics is the presence of a morphotropic phase boundary, where the crystal structure changes abruptly upon mixing, and the electromechanical (piezoelectric) properties become maximized.^439^ The observation of such a phase boundary in mixed lead halide perovskites would undoubtedly open new research and application frontiers of these materials.

## Abbreviations

AA: allylammonium
i-AM: isoamylammonium
ACE: acetamidinium cation
i-BA: isobutylammonium
AM: amylammonium
IM: imidazolium cation
AZR: aziridinium
i-PA: isopropylammonium
BA: butylammonium
MA: methylammonium cation
BPA: 3-bromopropylammonium
MACH: cyclohexanemethylammonium
Br-PEA: 4-bromophenethylammonium
MHy: methylhydrazinium cation
BZA: benzylammonium
PA: propylammonium
CHA: cyclohexylammonium
PEA: phenethylammoium
CPA: 3-chloropropylammonium
t-ACH: 4-aminomethyl-1-cyclohexanecarboxylate
DMA: dimethylammonium cation
t-BA: *tert*-butylammonium
EA: ethylammonium cation
TEA: thioethylammonium cation
Et_3_PrN: triethylpropylammonium
THTZ-H: tetrahydrotriazinium cation
FA: formamidinium cation
TZ: 1,2,4-triazolium
GA: guanidinium cation
3AMP: 3-(aminomethyl)piperidinium
HA: *n*-hexylammonium
4AMP: 4-(aminomethyl)piperidinium
HY: hydrazinium cation
4IBA: 4-iodobutylammonium
HEA: hydroxyethylammonium cation
ACI: alternating cations in the interlayer space
NMR: nuclear magnetic resonance
DFT: density functional theory
PL: photoluminescence
DJ: Dion–Jacobson
PXRD: powder X-ray diffraction
DS: dielectric spectroscopy
QENS: quasi elastic neutron scattering
DSC: differential scanning calorimetry
RP: Ruddlesden–Popper
IR: infrared
SCXRD: single crystal X-ray diffraction
MD: molecular dynamics
SHG: second-harmonic generation
MC: Monte Carlo
XRD: X-ray diffraction
NLO: nonlinear optical
